# Cuproptosis-related genes and agents: implications in tumor drug resistance and future perspectives

**DOI:** 10.3389/fphar.2025.1559236

**Published:** 2025-05-08

**Authors:** Lingwen Xu, Xiaolan Cao, Yuxiao Deng, Bin Zhang, Xinzhi Li, Wentao Liu, Wenjie Ren, Xuan Tang, Xiangyu Kong, Daizhou Zhang

**Affiliations:** ^1^ Institute of Chemical Drugs, Shandong Academy of Pharmaceutical Sciences, Jinan, China; ^2^ Shandong Provincial Key Laboratory of Carbohydrate and Glycoconjugate Drugs, Shandong Academy of Pharmaceutical Sciences, Jinan, China; ^3^ Department of Radiotherapy, Shandong Second Provincial General Hospital, Jinan, Shandong, China

**Keywords:** tumor drug resistance, cuproptosis, copper homeostasis, copper ionophores, chelators, nanodelivery

## Abstract

In the field of tumor treatment, drug resistance remains a significant challenge requiring urgent intervention. Recent developments in cell death research have highlighted cuproptosis, a mechanism of cell death induced by copper, as a promising avenue for understanding tumor biology and addressing drug resistance. Cuproptosis is initiated by the dysregulation of copper homeostasis, which in turn triggers mitochondrial metabolic disruptions and induces proteotoxic stress. This process specifically entails the accumulation of lipoylated proteins and the depletion of iron-sulfur cluster proteins within the context of the tricarboxylic acid cycle. Simultaneously, it is accompanied by the activation of distinct signaling pathways that collectively lead to cell death. Emerging evidence highlights the critical role of cuproptosis in addressing tumor drug resistance. However, the core molecular mechanisms of cuproptosis, regulation of the tumor microenvironment, and clinical translation pathways still require further exploration. This review examines the intersection of cuproptosis and tumor drug resistance, detailing the essential roles of cuproptosis-related genes and exploring the therapeutic potential of copper ionophores, chelators, and nanodelivery systems. These mechanisms offer promise for overcoming resistance and advancing tumor precision medicine. By elucidating the molecular mechanisms underlying cuproptosis, this study aims to identify novel therapeutic strategies and targets, thereby paving the way for the development of innovative anti-cancer drugs.

## 1 Introduction

**FIGURE 1:**
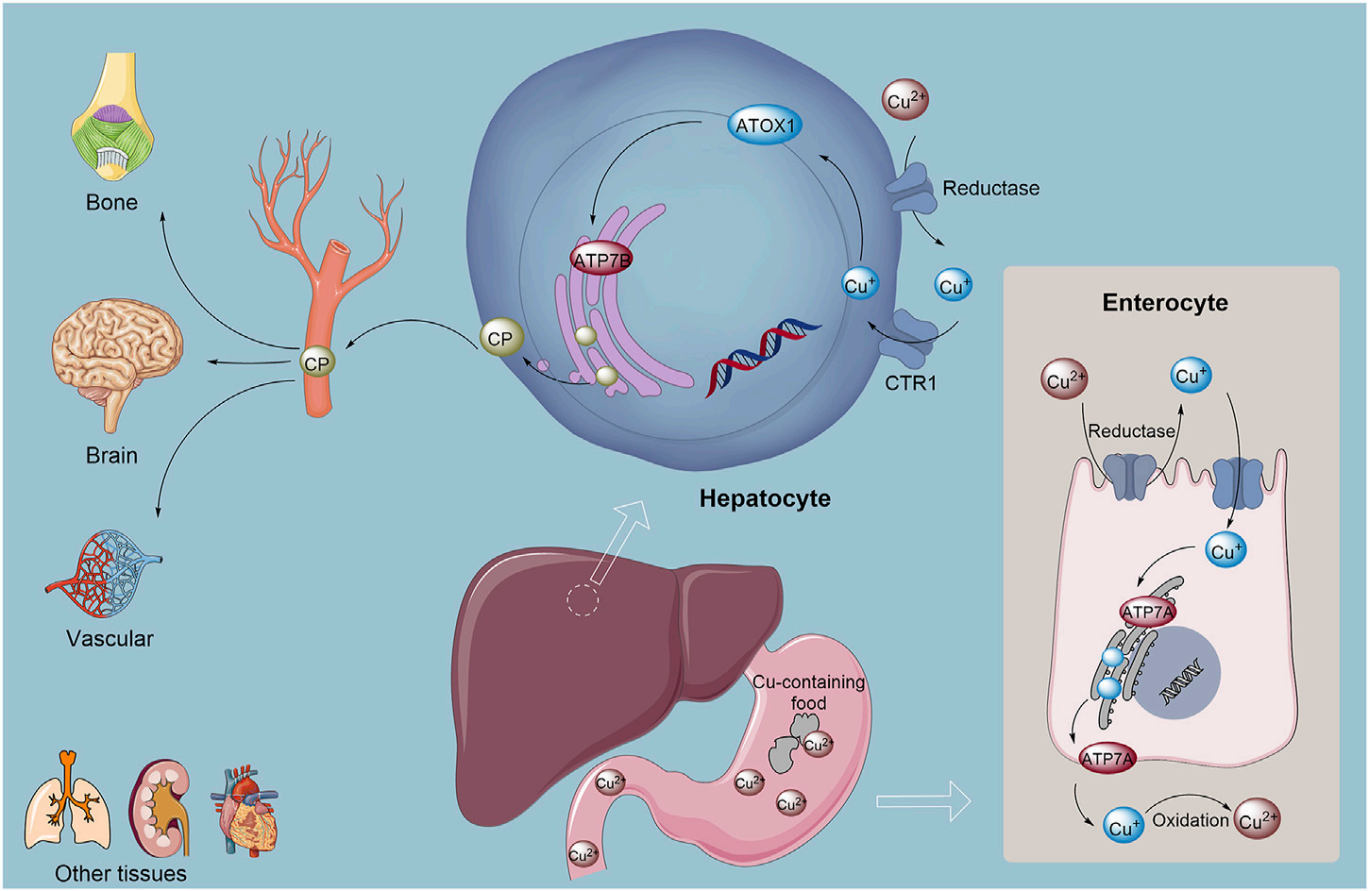
Copper absorption, metabolism, and homeostasis in the human body involve complex mechanisms. Cu^2+^ is initially absorbed from food in the stomach and small intestine. Subsequently, Cu^+^ is reduced to copper(I) by reductases, after which the monovalent copper binds to chaperone proteins, primarily ATOX1, and is transported to the liver. In the liver, ATP7B plays a crucial role in incorporating copper into ceruloplasmin, which serves as the copper carrier in the bloodstream, facilitating copper distribution to various tissues and mediating the excretion of excess copper into bile for elimination. Additionally, the transport protein ATP7A is responsible for exporting copper from intestinal epithelial cells into the bloodstream, where it aids in the oxidation of Cu^2+^ back to Cu^+^, promoting its systemic release and distribution. Abbreviation: ATOX1, Antioxidant 1 Copper Chaperon.

Copper is crucial in biological systems, acting as an essential cofactor for numerous enzymes and participating in key processes such as redox reactions, iron absorption and transport, neurotransmitter synthesis and degradation, and connective tissue formation (Chen et al., 2022; Pérez-Henarejos et al., 2015; Krupanidhi et al., 2008). As a vital constituent of several key enzymes, copper facilitates redox reactions and electron transfer, contributing to energy metabolism and protein synthesis (Chen et al., 2022; Rihel, 2018). It is also indispensable to the immune system, influencing immune responses and serving as a catalytic agent in various biochemical reactions that support immune cell function (Percival, 1998; Branch et al., 2022). In the nervous system, copper impacts neuronal excitability and synaptic transmission, participating in the metabolism of signaling molecules such as neuropeptides and hormones, which are essential for maintaining normal neural function (Gale and Aizenman, 2024). Furthermore, copper facilitates the synthesis and integrity of bones and connective tissues by aiding collagen stabilization, thus influencing the strength and stability of these structures (Han et al., 2024; Liu et al., 2024) (Figure 1). However, while copper ions are essential for various life processes, their accumulation can lead to cytotoxicity and contribute to diseases, including neurodegenerative disorders (Teschke and Eickhoff, 2024; Ejaz et al., 2020). Tumor cells exhibit a heightened demand for copper ion (Wu et al., 2018; Da Silva et al., 2022; Han et al., 2017). The critical roles of CTR1 in copper uptake and subsequently promoting tumorigenesis (Jiang et al., 2023). Higher copper levels have been observed in either serum or tumor tissues of breast cancer patients, and copper also plays a critical role in tumor cells and their microenvironments (Zhang X. et al., 2023). Elevated CTR1 expression is associated with heightened malignancy and unfavorable prognoses in cancers such as breast, liver, and lung. In addition, hyperactive CTR1 was investigated in pancreatic cancer tissues and proven to as the key point of cancer copper homeostasis (Geng et al., 2023). Targeting copper homeostasis, such as through the combined use of copper ion carriers and chelating agents, has emerged as a potential therapeutic strategy. Copper further promotes malignancy by activating proteins and signaling pathways involved in tumor progression. For instance, it facilitates metastasis by activating lysyl oxidase (LOX) and LOX-like proteins in conjunction with a mediator of cell motility 1 (MEMO1) (Shanbhag et al., 2019; Boufraqech et al., 2019; Tsai et al., 2012; Xiao and Ge, 2012). Additionally. Copper ions in tumor cells play dual roles in cancer progression. On one hand, they promote tumor growth and metastasis by activating oncogenic signals (such as MAPK/ERK, HIF-1α) and inducing EMT (Jiang Y. C. et al., 2022; Ruiz et al., 2021; Feng et al., 2024a). On the other hand, excessive copper ions can trigger copper-dependent cell death (Cuproptosis), selectively killing tumor cells by disrupting mitochondrial metabolism (Tsvetkov et al., 2022; Xie et al., 2023a). Their role depends on concentration, spatiotemporal distribution, and microenvironmental characteristics.

**FIGURE 2:**
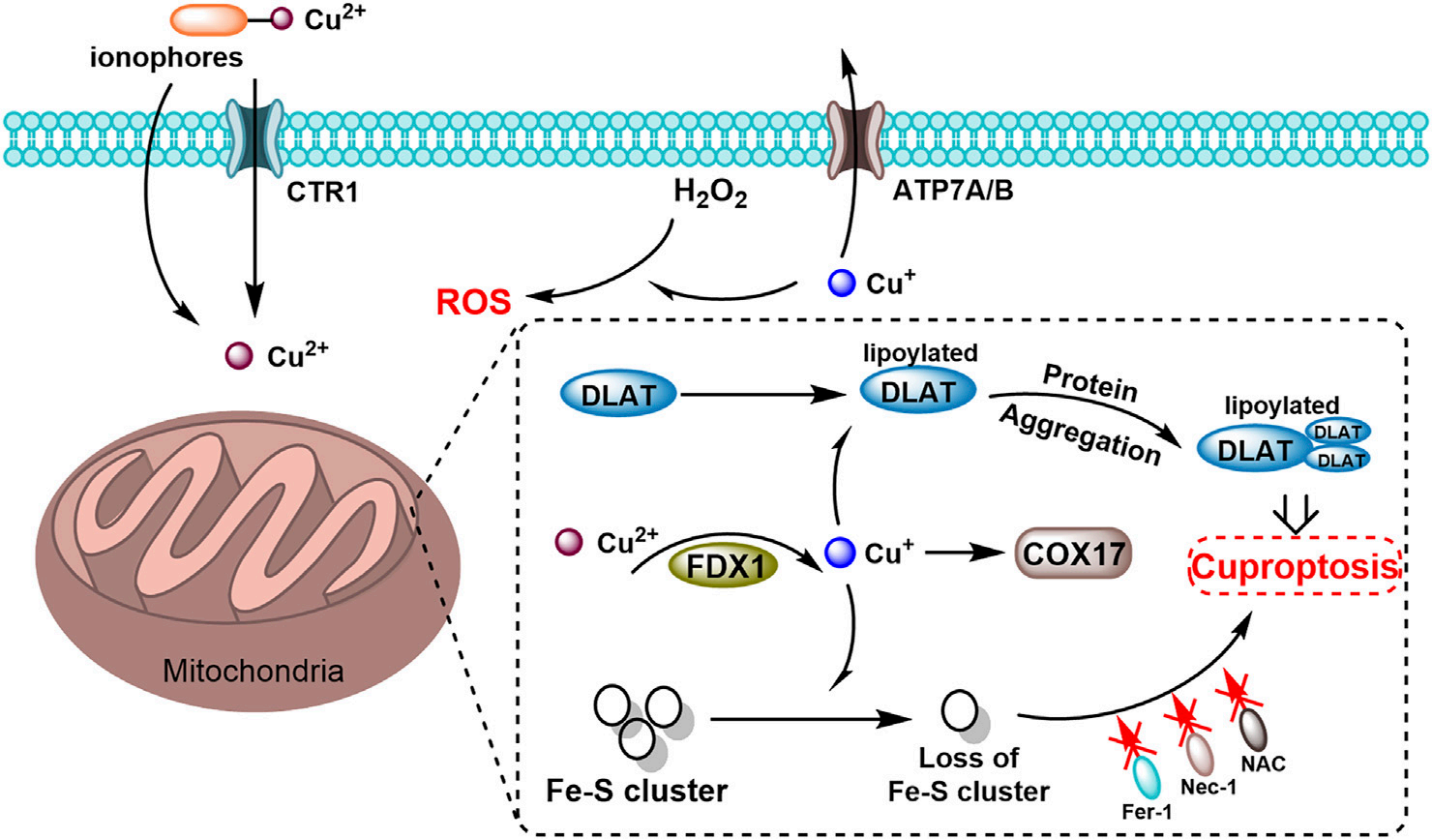
Schematic diagram of cuproptosis mechanism mediated by the copper ionophores. Copper ionophores facilitate the binding of extracellular copper and its transport into cells. Once internalized, copper interacts with lipoylated mitochondrial enzymes in the TCA cycle, including DLAT, promoting the aggregation of these enzymes. FDX1 (ferredoxin-1) key regulators of protein lipoylation, enhance the clustering of mitochondrial proteins and the depletion of iron-sulfur (Fe–S) clusters. These disruptions collectively induce proteotoxic stress, ultimately resulting in cell death.

Cuproptosis constitutes a unique form of copper-induced cell death, characterized by the aggregation of mitochondrial lipidated proteins and the instability of iron-sulfur cluster proteins. This process leads to a distinct form of regulated cell death (RCD) that differs from oxidative stress-related mechanisms such as apoptosis, ferroptosis, necrosis, and disulfidptosis (Tsvetkov et al., 2022; Xie et al., 2023a; Kahlson and Dixon, 2022). Apoptosis, a programmed cell death process actively executed under physiological or pathological stimuli, contrasts with passive necrosis, while ferroptosis is driven by iron-dependent phospholipid peroxidation (Gao et al., 2023a). Similarly, cuproptosis originates from mitochondrial copper overload-induced aggregation of lipoylated dihydrolipoamide S-acetyltransferase (DLAT), disrupting tricarboxylic acid (TCA) cycle homeostasis and triggering proteotoxic stress, whereas disulfidptosis rapidly ensues from disulfide stress due to aberrant cystine accumulation (Liu and Tang, 2023). The mechanism of cuproptosis involves several key steps. The excessive accumulation of copper within cells serves as the primary trigger for cuproptosis, which may arise from exogenous copper supplementation or the disruption of intracellular copper metabolism (Huang M. et al., 2024). Copper ionophores, exemplified by Elesclomol, feature specialized chemical moieties that enable high-affinity coordination with copper ions under physiological conditions, forming stable complexes with distinct physicochemical properties compared to free copper ions. These complexes exhibit enhanced membrane permeability, facilitating targeted copper transport into intracellular compartments with basal copper levels, thereby elevating localized copper concentrations (Huang M. et al., 2024; Tian et al., 2023; Huo et al., 2023; Zulkifli et al., 2023; Schulz et al., 2023; Yang Y. et al., 2022; Zheng et al., 2022). Once inside the cell, copper interacts with acetylated mitochondrial enzymes in the TCA cycle, such as DLAT, resulting in the aggregation of these enzymes. This interaction alters the structure and function of mitochondrial proteins (Huang M. et al., 2024; Tian et al., 2023). Additionally, ferredoxin 1 and lipoic acid synthetase (LIAS), which act as upstream regulators of protein acetylation, play a crucial role. By modulating acetylation, they facilitate the aberrant aggregation of mitochondrial proteins and the loss of iron-sulfur (Fe-S) clusters (Wang M. et al., 2023; Garza et al., 2023) (Figure 2). The depletion of Fe-S clusters disrupts the function of several key enzymes, particularly those involved in energy metabolism and oxidative stress response. The emergence of the mechanism of cuproptosis may play a crucial role in the treatment of diseases such as malignancies, neurodegenerative diseases, and Wilson disease (Kahlson and Dixon, 2022; Tong et al., 2022; Qin et al., 2023; Zhang Y. et al., 2023; Huang, 2023). In cancer, cuproptosis has demonstrated significant therapeutic potential. Studies have shown that the accumulation of copper and the application of copper ionophores can selectively induce cell death in various cancer types, including breast cancer, prostate cancer, and melanoma, which are particularly sensitive to copper-induced cell death (Kahlson and Dixon, 2022; Tong et al., 2022; Qin et al., 2023). Pan-cancer analysis has revealed that cuproptosis related genes, such as ATP7B, ATP7A, and CDKN2A, harbor high-frequency mutations or abnormal expression patterns in various types of cancer (Liu, 2023; Liu and Tang, 2022a; Liu and Tang, 2022b). The copy number variations (CNV), methylation status, and miRNA regulatory networks of these genes are significantly correlated with patient survival, tumor microenvironment, and chemotherapy drug sensitivity (Liu, 2023; Liu and Tang, 2022a). These findings suggest their potential as cross-cancer diagnostic and prognostic markers. In kidney renal clear cell carcinoma (KIRC), low expression of copper death-promoting genes, such as FDX1 and DLAT, is associated with better survival, whereas high expression of copper death-resistant genes, including CDKN2A and ATP7B, indicates a poor prognosis (Liu and Tang, 2022b).

According to the data of “cancer statistics, 2024”, the overall 5-year relative survival rate for all cancers combined was 68% between 2013 and 2019 (Siegel et al., 2024). However, significant disparities persisted among cancer types: pancreatic (13%), liver (22%), and lung (25%) cancers exhibited markedly lower survival rates compared to thyroid (99%) and prostate (97%) cancers (Sonkin et al., 2024). Growing recognition of tumor complexity and heterogeneity underscores the urgent need for reliable prognostic biomarkers to optimize personalized therapeutic strategies. Current cornerstone interventions include surgical resection for localized tumors, systemic cytotoxic chemotherapy, localized radiotherapy, molecularly targeted therapies against driver mutations, and immune checkpoint inhibitors (Sonkin et al., 2024). Emerging modalities such as CRISPR-based gene editing, nanoparticle-based drug delivery systems, and photodynamic therapy continue to expand the oncological armamentarium, offering novel avenues for precision oncology (Sonkin et al., 2024). Tumor drug resistance is a complex phenomenon that results from multiple factors. One major factor is the overexpression of ATP-binding cassette (ABC) transporters. For example, the ABCB1 transporter, also known as P-glycoprotein, has been widely studied. Gupta et al. (2019) demonstrated that ABCB1 actively pumps out various chemotherapeutic drugs, such as doxorubicin and paclitaxel, from cancer cells, thereby reducing intracellular drug concentration and leading to drug resistance. Another factor involves the activation of DNA repair pathways. The DNA damage response pathway, including proteins such as BRCA1 and BRCA2, repairs DNA damage caused by chemotherapeutic agents (Panda et al., 2018; Mehta et al., 2021; Mirman et al., 2022). As reported by Lin et al. (2020), breast cancer cells with mutated BRCA1 exhibited enhanced resistance to platinum-based chemotherapy drugs due to the activation of alternative DNA repair mechanisms. In addition, activation of the PI3K/Akt/mTOR signaling pathway promotes cell proliferation and growth, leading to resistance to apoptosis-inducing drugs (Gou et al., 2022; Nath et al., 2022). Wang et al. (Fujimoto et al., 2018) demonstrated that constitutive activation of the PI3K/Akt/mTOR pathway in colorectal cancer cells resulted in resistance to 5-fluorouracil treatment. The MAPK/ERK pathway also plays a crucial role by regulating cell proliferation and differentiation, and its abnormal activation is associated with drug resistance. Wang et al. (2024a) found that overactivation of the MAPK/ERK pathway contributed to resistance to tyrosine kinase inhibitors in lung cancer cells. In addition, the ovarian cancer xenograft model confirms that the crosstalk between PI3K/AKT/mTOR and MAPK/ERK pathways can mediate chemoresistance (Wang L. et al., 2024). Targeting the MAPK/ERK pathway combined with PARP inhibitors significantly reduces tumor burden and extends survival (Li R. et al., 2018). Recent studies suggest that alterations in the cuproptosis pathway can influence tumor drug resistance (Wang Y. et al., 2024; Liu et al., 2023; Zhang et al., 2025). Some cuproptosis-related genes, such as FDX1, are upregulated in drug-resistant cancer cells. As shown by Wang et al. (Huang et al., 2025), restoring FDX1 expression in drug-resistant ovarian cancer cells sensitized them to cisplatin treatment by promoting cuproptosis. Moreover, copper ionophores, which induce cuproptosis, may have the potential to overcome drug resistance. Wang et al. (2024b) demonstrated that a novel ES-Cu nanoparticles copper ionophore reversed resistance in paclitaxel-resistant cell lines by inducing cuproptosis, suggesting that targeting the cuproptosis mechanism may offer a promising strategy to combat tumor drug resistance.

This review provides a comprehensive overview of recent advancements in cuproptosis and its potential therapeutic applications in overcoming tumor resistance. We discuss the critical role of copper in cellular processes and its dysregulation, which selectively induces the death of cancer cells. Additionally, we summarize the mechanisms through which copper ionophores, chelators, and nanodelivery systems facilitate cuproptosis, and assess their therapeutic efficacy in preclinical cancer models. Importantly, we highlight the emerging role of cuproptosis-related genes and agents in counteracting tumor drug resistance, with a focus on mechanisms such as the regulation of mitochondrial metabolism and stress responses, which contribute to therapy evasion in resistant cancer cells. This review integrates recent findings to demonstrate how cuproptosis-based strategies can circumvent resistance, thereby laying the groundwork for the development of novel therapeutic approaches. By combining knowledge of cuproptosis mechanisms with their implications for drug resistance, this review provides valuable perspectives on innovative directions for advancing cancer therapies and addressing the persistent challenge of tumor resistance.

## 2 Key cuproptosis genes related to tumor drug resistance

Cuproptosis, in the context of antitumor resistance research, is intricately linked to several critical genes, including copper transporter 1 (CTR1/SLC31A1), antioxidant 1 copper chaperone (ATOX1), ATP7A/B, cytochrome c oxidase (COX), and Ferredoxin 1 (FDX1) (Table 1). These genes play vital roles in copper uptake, intracellular transport, and excretion. For instance, reduced CTR1 expression in specific tumor cells limits copper ion influx, diminishing the efficacy of chemotherapy drugs—particularly platinum-based agents that depend on copper metabolism—thereby contributing to drug resistance. Conversely, overexpression of ATP7A and ATP7B enhances copper ion efflux, leading to reduced intracellular copper concentrations. As a result, tumor cells exhibit reduced susceptibility to copper-induced cell death, which may contribute to resistance against drugs such as docetaxel. Furthermore, elevated nuclear FOXO3 levels may suppress the transcription of critical cuproptosis-related genes, such as FDX1, thereby impairing copper-induced cell death and fostering chemotherapy resistance in tumor cells. A thorough analysis of these key genes can deepen our understanding of cuproptosis mechanisms and support the development of novel therapeutic strategies to counteract tumor resistance.

**TABLE 1:** Functions of cuproptosis-related genes and their roles in the tumor drug resistance mechanism.

Gene	Full name	Subcellular locations	Functions	The role in the mechanism of tumor drug resistance	Ref
CTR1 /SLC31A1	Copper Transporter 1/Solute Carrier Family 31 Member 1	Plasma membrane	Regulating cellular copper uptake, copper metabolism, and vital physiological functions	Downregulation of CTR1 promotes cuproptosis resistance by influencing the expression of ATP7A/7B	Jandial et al. (2009), Ishida et al. (2013), Zhang et al. (2022a)
ATP7A	ATPase Copper Transporting Alpha	Golgi apparatus	Transmembrane Cu^+^ transport regulates intracellular levels, influencing oxidative stress and metabolic balance	Low expression of ATP7A could enhance the sensitivity of tumor cells to cuproptosis-inducing platinum drugs	Samimi et al. (2004), Shao et al. (2023)
ATP7B	ATPase Copper Transporting Beta	Golgi apparatus	ATP-driven Cu^+^ pump that plays an important role in intracellular copper homeostasis	Upregulation of ATP7B protein leads to a decrease in intracellular copper levels, subsequently resulting in docetaxel resistance	Petruzzelli et al. (2022), Li et al. (2024a)
ATOX1	Antioxidant 1 Copper Chaperone	Nucleoplasm, Plasma membrane	A crucial copper transport protein that maintains intracellular copper homeostasis	Copper-induced chemoresistance and DNA damage repair rely on ATOX1	Salguero et al. (2019), Itoh et al. (2008), Jin et al. (2022)
FDX1	Ferredoxin 1	Mitochondrion	Encodes a small iron-sulfur protein that transfers electrons from NADPH through ferredoxin reductase to mitochondrial cytochrome P450, involved in steroid, vitamin D, and bile acid metabolism	Upregulation of FDX1 expression leads to acquiring cisplatin resistance	Takahashi et al. (2023)
DLAT	Dihydrolipoamide	Mitochondrion	Catalyzes the overall conversion of pyruvate to acetyl-CoA and CO_2_	Upregulation of DLAT inhibited autophagy, promoted cell retention in the G2/M phase, and enhanced docetaxel chemotherapy sensitivity both *in vitro* and *in vivo* through the mTOR signaling pathway	Wen et al. (2023), Xia et al. (2024a)
CDKN2A	Cyclin Dependent Kinase Inhibitor 2A	Nucleoplasm	Capable of inducing cell cycle arrest in G1 and G2 phases	CDKN2A orchestrates cuproptosis resistance via modulating glycolysis and copper homeostasis	Wan et al. (2023), Cheng et al. (2024)
CEBPB	CCAAT/enhancer-binding protein beta	Nucleoplasm	Regulating immune and inflammatory responses	Activates the PI3K/AKT/mTOR signaling pathway to dampen cuproptosis sensitivity	Huang et al. (2024c), Barakat et al. (2016), Wang et al. (2019)
NUDT21	Nudix hydrolase 21	Nuclear, Centriolar satellite	An activator of the pre-mRNA 3′-end cleavage and polyadenylation processing	NUDT21-mediated unmethylated 3′UTR shortening underlies the emergence of enzalutamide resistance and insensitivity to cuproptosis in prostate cancer cells	Zhou et al. (2024), Xiao et al. (2023), Lin et al. (2024)
YTHDC2	YTH N6-methyladenosine RNA binding protein 2	Cytosol, Cytoplasmic bodies	A regulator for neural development via facilitating m6A-dependent degradation of mRNA targets related to neural development	The downregulation of YTHDC2 potentially induces resistance to cuproptosis in colorectal cancer	Shen et al. (2023), Zhao et al. (2024)
FOXO3	Forkhead box O3	Nucleoplasm	A trigger for apoptosis through expression of genes necessary for cell death	The elevated level of FOXO3 in the nucleus suppressed FDX1 transcription and cellular cuproptosis, consequently promoting chemoresistance	Wang et al. (2023c)

### 2.1 Copper transporter 1, CTR1

CTR1, or Solute Carrier Family 31 Member 1 (SLC31A1), is a high-affinity copper transporter essential for copper import and metabolism (Das et al., 2022; Chen et al., 2024a; Batzios et al., 2022). Primarily localized in the plasma membrane, CTR1 facilitates the uptake of copper from the extracellular environment and is additionally found in intracellular compartments such as endosomes and the Golgi apparatus, where it contributes to copper distribution and regulation (Öhrvik et al., 2016). CTR1’s localization and function are stringently regulated to maintain copper homeostasis, balancing sufficient copper availability for essential processes with preventing toxic accumulation. Dysfunction or altered expression of CTR1 can cause marked changes in intracellular copper levels, potentially initiating copper-induced cell death (Das et al., 2022; Levy et al., 2017). CTR1 primarily imports copper into the cytosol and distributes it to organelles like mitochondria and the Golgi apparatus for enzyme activation (Öhrvik and Thiele, 2015; Chen et al., 2020).

CTR1 could amplify copper uptake, inducing mitochondrial copper accumulation, dysfunction, and reactive oxygen species (ROS) production, ultimately leading to cell death (Wee et al., 2013; Guo et al., 2021; Safi et al., 2014; Parmar et al., 2018). Additionally, the C-terminal HCH sequence of CTR1 regulates the cellular uptake of copper ions and platinum-based chemotherapeutics (Jandial et al., 2009). Both its extracellular N-terminus and intracellular C-terminus contain metal-binding residues that facilitate platinum drug transport (Ishida et al., 2013; Zhang P. et al., 2022). Evidence suggests that reduced CTR1 expression in cancer cells contributes to resistance against platinum-based drugs, whereas enhanced expression sensitizes cells to these therapies (Tsimberidou et al., 2015; Skowron et al., 2018; Kim et al., 2014; Kuo et al., 2021). In platinum-resistant colorectal cancer cells, the expression of copper/platinum transporter CTR1 is significantly reduced, leading to decreased intracellular uptake of platinum drugs such as oxaliplatin, thereby diminishing the therapeutic effect. Azacitidine, a DNA methylation inhibitor, can restore CTR1 expression levels by reversing the hypermethylation status of the CTR1 promoter. This epigenetic regulation may enhance the cytotoxicity of oxaliplatin and overcome platinum resistance (Tsimberidou et al., 2015). In urothelial cancer cell lines treated with cisplatin for a long time, both mRNA and protein expression levels of CTR1 (Copper Transporter 1) are significantly reduced, which is directly correlated with decreased cisplatin uptake. Compared to non-resistant parental cells, the expression of CTR1 in resistant cell lines decreases by 50%–70% (verified by Western blot and qRT-PCR), suggesting that low CTR1 expression is one of the key mechanisms of acquired resistance (Skowron et al., 2018). In tumor tissues of patients with non-small cell lung cancer (NSCLC), there is a significant positive correlation between the protein expression level of CTR1 and the local tissue concentration of platinum-based chemotherapy drugs such as cisplatin and carboplatin (r = 0.58, p < 0.001). The accumulation of platinum drugs in tumors with high CTR1 expression is 2–3 times higher than that in the low expression group (Kim et al., 2014). Cells with a lower basal level of hCtr1 show a greater upregulation of hCtr1 under the action of Cu chelators compared to cells with a higher basal level of hCtr1 (Kuo et al., 2021). Recent research highlights CTR1’s role in mediating intracellular copper transport critical for copper-dependent cytotoxicity. However, in another study, elevated CTR1 levels were observed in two cisplatin-resistant cell lines, cervical adenocarcinoma KB-CP20 and hepatoma BEL-7404-CP20. These 2 cell lines originate from different cell types, and the precise mechanism behind the higher CTR1 levels in highly cisplatin-resistant cells remains unclear. It could potentially be a result of mislocalization or defects in post-translational modifications (Fung et al., 2014). These findings position CTR1 as a potential biomarker for evaluating the efficacy of platinum-based chemotherapeutics, including cisplatin, carboplatin, and oxaliplatin.

### 2.2 ATP7A and ATP7B

ATP7A and ATP7B are key members of the copper-transporting ATPase family, encoding proteins that play a crucial role in regulating intracellular copper homeostasis (Singla et al., 2021; Yu et al., 2017). ATP7A is broadly expressed across various tissues, whereas ATP7B is primarily localized in the liver (Yu et al., 2017). Both proteins regulate cellular copper homeostasis through copper transport, and their dysfunction is strongly associated with a variety of human diseases, including cancer (Li Y. Q. et al., 2018). ATP7A is essential for transmembrane copper transport, influencing cellular oxidative stress and metabolic processes by modulating intracellular copper levels. Elevated ATP7A expression has been observed in multiple cancer types, correlating with tumor growth and invasiveness (Samimi et al., 2004; Shao et al., 2023). Research has shown that ATP7B overexpression reduces copper accumulation and accelerates copper efflux, thereby diminishing the efficacy of disulfiram (DSF) in cancer cells. Furthermore, the HIF-1 signaling pathway is suppressed in ATP7B knockout HepG2 cells (Mi et al., 2020).

The copper transporters ATP7A and ATP7B primarily mediate drug resistance in cancer cells via two distinct mechanisms. First, these transporters, which participate in copper chelation and excretion, can expel platinum-based drugs similarly to copper efflux (Arnesano and Natile, 2021). Upregulation of ATP7B expression in a human endometrial cancer cell line has been reported to significantly reduce the intracellular concentration of platinum-based drugs (Aida et al., 2005). Second, ATP7A and ATP7B can inactivate platinum-based drugs via chelation. Studies have shown that both ATP7A and ATP7B proteins contain six repeated metal-binding domains (MBDs) at their N-termini, with each binding domain consisting of approximately 70 amino acid residues. The highly conserved CXXC sequence within the MBDs of ATP7A and ATP7B serves as the platinum-binding site, leading to the inactivation of platinum-based drugs via chelation (Dolgova et al., 2009). Overexpression of miR-148a-3p downregulates ATP7A, reversing cisplatin resistance by blocking drug efflux *in vitro* and in xenograft models (Yu et al., 2020). High ATP7B expression in ovarian tumor biopsies correlates with poor platinum response (reduced progression-free survival) in retrospective cohort studies (Lukanović et al., 2020). Tetrathiomolybdate, a copper chelator, inhibits ATP7B activity in ovarian cancer models, restoring cisplatin sensitivity by blocking drug export (Ryumon et al., 2019; Fang et al., 2019). Studies have shown that elevated MBD expression in cells can enhance resistance to platinum-based drugs (Dolgova et al., 2009). Additionally, DSF and copper interact with ATP7B to decrease the protein levels of COMM domain-containing protein 1 (COMMD1), S-phase kinase-associated protein 2 (Skp2), and clusterin, while significantly increasing the expression of cyclin-dependent kinase inhibitor 1 (p21/WAF1) (Song et al., 2024). Research indicates that docetaxel-resistant prostate cancer cells exhibit copper-dependent nutritional vulnerability via the ATP7B efflux transporter, thereby improving docetaxel treatment efficacy (Song et al., 2024).

**FIGURE 3:**
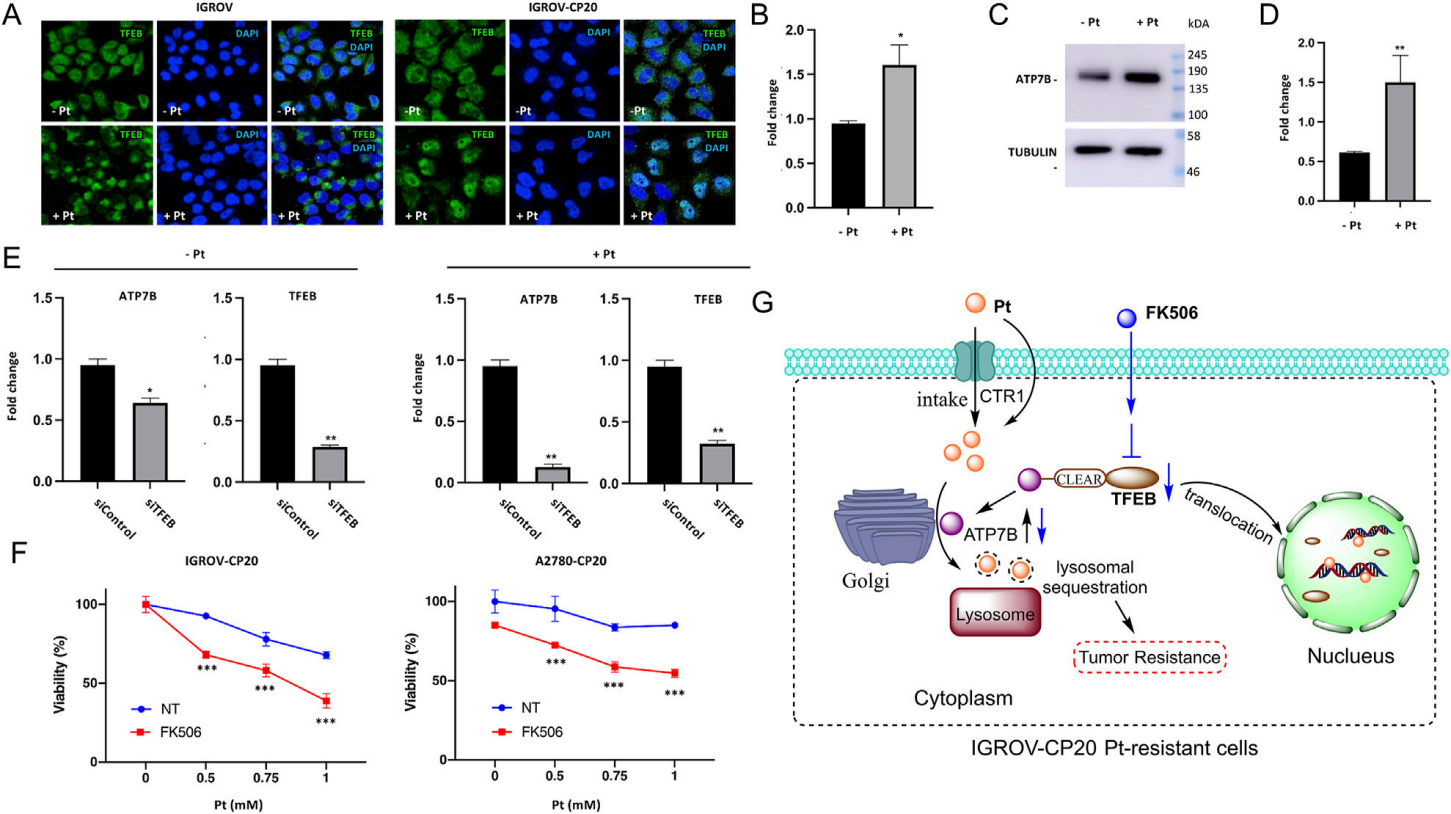
TFEB regulates the expression levels of ATP7B to control the proliferation of platinum-resistant ovarian cancer cells. **(A)** IGROV and IGROV-CP20 cells were subjected to fixation for confocal microscopy either directly (Pt-) or after a 24-h incubation with 50 μM cisplatin (Pt+). Representative confocal images demonstrated that cisplatin treatment induced the translocation of TFEB to the nucleus in resistant IGROV-CP20 cells, but not in the parental IGROV cells. ATP7B mRNA **(B)** and protein levels [**(C)** and quantification in **(D)**] were increased in IGROV-CP20 cells treated with 50 uM Pt compared to the untreated condition. **(E)** The expression levels of ATP7B were significantly reduced upon silencing of TFEB (siTFEB) compared to the scramble control (siControl), as determined by RT-qPCR analysis. **(F)** IGROV-CP20 and A2780-CP20 resistant cells were treated with 10 μM FK506 and then exposed to increasing concentrations of cisplatin (Pt). The results of the MTT assay indicated that FK506 enhanced the cytotoxicity of Pt in both cell lines. (Reprinted with permission from Ref. (Petruzzelli et al., 2022). Copyright 2022 by the authors). **(G)** TFEB modulates ATP7B expression to facilitate platinum chemoresistance in human ovarian cancer cells.

Altered ATP7B expression in cancer is also associated with tumor progression and drug resistance. Research indicates that ATP7B can influence the resistance to copper-based anticancer agents, as some tumor cells may upregulate ATP7B to expel intracellular copper, thereby reducing the cytotoxicity of these drugs (Petruzzelli et al., 2022; Li P. et al., 2024). Elevated ATP7B expression and its post-Golgi trafficking have been linked to tumor cell resistance to Pt-based chemotherapy (Petruzzelli et al., 2022). In this context, researchers have identified a novel transcriptional mechanism that regulates ATP7B expression in drug-resistant tumor cells through Pt-dependent transcription factor EB (TFEB) activation (Petruzzelli et al., 2022). ATP7B contributes to platinum resistance by actively exporting cisplatin and carboplatin out of ovarian cancer cells (Arnesano and Natile, 2021). Meanwhile, ATP7B redirects platinum drugs into subcellular compartments (e.g., lysosomes or Golgi apparatus), preventing their interaction with nuclear DNA and reducing DNA damage-induced apoptosis (Tadini-Buoninsegni et al., 2014). In IGROV-CP20 (cisplatin-resistant) cells, Pt induces the translocation of TFEB from the cytoplasm to the nucleus, while in parental IGROV cells, TFEB remains in the cytoplasm regardless of cisplatin exposure (Figure 3A). To assess this, mRNA and protein expression levels of ATP7B were analyzed using qRT-PCR and Western blot, respectively. As shown in Figures 3A,B, both mRNA and protein levels of ATP7B were elevated in platinum-treated cells (Figures 3B,C). Additionally, researchers silenced TFEB in both control and Pt-treated cells to assess the impact of silencing on ATP7B expression (Figure 3D) (Petruzzelli et al., 2022). qRT-PCR data revealed that TFEB depletion in control IGROV-CP20 cells moderately reduced ATP7B expression (Figure 3E). Conversely, IGROV cells treated with Pt exhibited a significantly greater degree of ATP7B downregulation following TFEB knockdown (Figure 3E). Furthermore, treatment of drug-resistant cells with the TFEB inhibitor FK506 showed that FK506 significantly increased Pt toxicity in resistant IGROV-CP20 cells, indicating that TFEB nuclear translocation is essential for drug resistance (Figures 3F,G). Researchers have also reported that combining the apoptosis inducer disulfiram with anti-PD-L1 effectively overcomes drug resistance in non-small cell lung cancer (NSCLC) by regulating the ATP7B-mediated HIF-1 signaling pathway (Ryumon et al., 2019).

### 2.3 Cytochrome c oxidase, COX

COX is a crucial enzyme in the mitochondrial respiratory chain that catalyzes the reaction between cytochrome c and oxygen to produce water, an essential component for ATP synthesis (Brischigliaro and Zeviani, 2021; Ishigami et al., 2023). Recent studies have highlighted the integral role of COX in the mechanism of cuproptosis, particularly concerning oxidative stress and mitochondrial dysfunction induced by copper (Swaminathan and Gohil, 2022; Garza et al., 2021). Copper-induced cell death involves the excessive accumulation of copper within cells, leading to increased production of ROS, which causes oxidative damage. Given COX’s central role in the respiratory chain, its activity is significantly influenced by ROS levels. Elevated intracellular copper concentrations can impair COX functionality, leading to decreased efficiency of the mitochondrial respiratory chain. This dysfunction not only impairs ATP generation but may also promote apoptosis or necrosis, establishing a vicious cycle that exacerbates cellular oxidative stress (Rebollo-Hernanz et al., 2022; Xue et al., 2020). The cytochrome c oxidase copper chaperone COX17 is essential in copper metabolism and mitochondrial function, significantly contributing to the process of cuproptosis (Wang et al., 2023b). COX17 facilitates the transport of copper ions into the mitochondria, which is critical for the assembly and activity of cytochrome c oxidase, essential for cellular respiration (Huang D. et al., 2024; Zhu et al., 2023). In the context of cancer, COX17’s role in promoting copper accumulation can lead to mitochondrial dysfunction and increased ROS production (Vanišová et al., 2019). This oxidative stress can damage cellular components, activating pathways that culminate in cell death. COX17 regulates apoptotic signaling by modulating the balance between pro-apoptotic and anti-apoptotic signals, potentially activating caspases that execute the apoptotic process (Sotgia et al., 2017). Moreover, COX17 interacts with a network of copper transporters, such as CTR1, ATP7A, and ATP7B, maintaining copper homeostasis within the cell (Wang et al., 2023b; Cheng et al., 2017). Dysregulation of this network can disrupt COX activity and overall cellular metabolism. The oxidative stress resulting from COX17-mediated copper accumulation can also activate critical signaling pathways, including the p53 and MAPK pathways, thereby linking copper metabolism to cell growth and survival (Xue et al., 2023). Furthermore, there may be crosstalk between the cuproptosis and ferroptosis pathways, as both involve iron-sulfur cluster proteins and oxidative stress. Regulation of COX17 expression may improve the efficacy of copper-based therapies, particularly in tumors sensitive to copper-induced cytotoxicity.

### 2.4 Antioxidant 1 copper chaperone, ATOX1

ATOX1 is an essential copper transport protein that maintains intracellular copper homeostasis (Cai et al., 2014; Yang et al., 2023). Predominantly located in the cytoplasm, ATOX1 binds copper, maintaining it in a non-toxic form and facilitating its transport to ATP7A and ATP7B, copper-transporting ATPases located in the trans-Golgi network (Hatori et al., 2017). These ATPases subsequently incorporate copper into various enzymes and proteins involved in the secretory pathways. Furthermore, ATOX1 may have nuclear functions, as it can translocate to the nucleus and participate in gene regulation, particularly under conditions of oxidative stress or elevated copper levels (Sudhahar et al., 2022; Weishaupt et al., 2024). In the context of copper-mediated cell death, ATOX1 collaborates with ATP7A and ATP7B to transport copper to the Golgi apparatus. Research suggests that modulating ATOX1 expression levels significantly impacts copper metabolism and cuproptosis mechanisms, with important implications for tumor cell growth and survival. ATOX1 is also critical to the cellular antioxidant defense system (Rivera et al., 2023). Through its regulation of copper transport and metabolism, ATOX1 reduces oxidative stress levels, thereby inhibiting tumor cell proliferation.

In a study investigating damage to normal tissue caused by platinum-based chemotherapy drugs for tumors, researchers induced protein overexpression via lentiviral transfection targeting ATOX1. The subsequent biological activity evaluation revealed that overexpression of ATOX1 led to a significant increase in the levels of ATOX1, CTR1, and SOD3 in HEI-OC1 cells. Meanwhile, the expression levels of ATP7A and ATP7B proteins were significantly reduced. Furthermore, it was demonstrated that overexpression of ATOX1 intervention effectively reduced the apoptosis rate and the number of cells in the G2/M phase (Chen et al., 2024b). In another example, ATOX1 promotes double-strand DNA repair by transcriptionally upregulating the expression of a mediator of DNA Damage Checkpoint 1 (MDC1) (Salguero et al., 2019; Itoh et al., 2008). Knockdown of ATOX1 results in a significant reduction in MDC1 expression and delays the phosphorylation of γ-H2AX, indicating that MDC1 activity is blocked after DNA damage. It has been demonstrated that ATOX1 translocates to the nucleus and binds to the MDC1 promoter in a copper-dependent manner to activate its transcription. Furthermore, research indicates that ATOX1 predominantly facilitates DNA damage repair in gemcitabine-treated Patu 8988T cells via the NHEJ-mediated pathway, resulting in drug resistance (Jin et al., 2022). ATOX1 also interacts with other copper transport proteins, including ATP7A and ATP7B, which are vital for copper metabolism and transport (Spiers et al., 2021). These interactions can influence the sensitivity of tumor cells to copper-based therapies.

### 2.5 Ferredoxin 1, FDX1

FDX1 is a crucial mitochondrial iron-sulfur protein essential for cellular electron transfer and metal cofactor biosynthesis (Lei et al., 2023; Tsvetkov et al., 2019). FDX1 is indispensable for Fe-S cluster metabolism, orchestrating electron transfer, cluster assembly, and redox balance (Sheftel et al., 2010; Marelja et al., 2018). Its roles span mitochondrial energy production, cytosolic enzyme activation, and cellular stress response (Sheftel et al., 2010; Marelja et al., 2018). FDX1’s involvement in copper-mediated cell death, known as cuproptosis, has garnered significant attention. FDX1 is critical for copper metabolism, particularly in synthesizing iron-sulfur clusters, which are essential components of the mitochondrial respiratory chain. Copper accumulation can disrupt the synthesis or function of these clusters, leading to mitochondrial dysfunction and the initiation of cuproptosis (Yang L. et al., 2022; Zhang Z. et al., 2022; Yang S. et al., 2024). Impairment of FDX1 may further exacerbate this dysfunction, thereby promoting tumor cell death. Furthermore, FDX1 participates in redox reactions within mitochondria, where it can reduce divalent copper to its monovalent form. In the context of cuproptosis, excessive copper accumulation increases ROS production, damaging mitochondrial integrity and inducing apoptosis or necrosis. Thus, FDX1 plays a critical role in regulating oxidative stress responses during copper-mediated cell death (Quan et al., 2023; Wang et al., 2022).

Given its central role in the mechanisms of cuproptosis, targeting FDX1 holds significant research and clinical potential for anti-tumor resistance strategies. Developing small-molecule inhibitors of FDX1 could enhance copper accumulation in tumor cells, thereby promoting cuproptosis, particularly in malignancies that rely on mitochondrial respiration, such as certain ovarian cancers and clear cell renal cell carcinoma (ccRCC). Research has demonstrated that FDX1 is upregulated in cisplatin-resistant cells. To further investigate its role in platinum-based tumor drug resistance, researchers examined the cisplatin-resistant cell lines A2789cis and OVK18cis (Takahashi et al., 2023). Results indicated that FDX1 may contribute to cisplatin resistance in ovarian cancer cells by inhibiting the increase in mitochondrial membrane potential and cisplatin-induced lipid peroxidation. Furthermore, suppressing FDX1 expression can induce apoptosis in cisplatin-resistant cells (Takahashi et al., 2023). Conversely, the upregulation of adrenomedullin (ADM) was found to inhibit FDX1 expression and mediate sunitinib resistance in ccRCC by facilitating FOXO3 phosphorylation and its nuclear translocation (Wei et al., 2024; Lin Z. et al., 2020; Bing et al., 2021; Wang et al., 2023c). Experimental data suggest that the ADM/FOXO3/FDX1 axis may constitute a crucial pathway in sunitinib resistance in ccRCC (Wang et al., 2023c). In another study, excess copper in triple-negative breast cancer (TNBC) activates AKT1, which is associated with resistance to copper toxicity. Specifically, AKT1 inhibits protein lipoylation by phosphorylating FDX1 while regulating tumor metabolic reprogramming, ultimately suppressing cuproptosis (Sun Z. et al., 2025). Future research should further elucidate the molecular mechanisms of FDX1 in cuproptosis and explore its potential roles in tumor resistance. A deeper understanding of the interplay between FDX1 and cuproptosis may foster the development of more effective and safer anti-tumor therapies, particularly for challenging malignancies where cuproptosis mechanisms could offer novel treatment options.

### 2.6 Other key genes of cuproptosis for tumor resistance

DLAT has been identified as a key mediator of cuproptosis-enhanced sensitivity to docetaxel by inhibiting autophagy and promoting G2/M phase arrest through activation of the mTOR signaling pathway, thereby enhancing docetaxel efficacy both *in vitro* and *in vivo* (Wen et al., 2023; Xia C. et al., 2024). Cyclin Dependent Kinase Inhibitor 2A (CDKN2A) is associated with increased genomic instability and enhanced sensitivity to radiation and chemotherapy (Horn et al., 2018; Wu et al., 2022). Tumor regions expressing CDKN2A exhibit distinct infiltration of SPP1(+) tumor-associated macrophages (TAMs), MMP7 enrichment, and unique signaling interactions with adjacent regions (Wan et al., 2023). Moreover, CDKN2A promotes cuproptosis resistance by modulating glycolysis and copper homeostasis, thereby contributing to a malignant phenotype and a pro-tumor microenvironment (Cheng et al., 2024). Elevated expression of CCAAT/enhancer-binding protein beta (CEBPB) accelerates the malignant progression of colorectal cancer (CRC) by activating the PI3K/AKT/mTOR signaling pathway and decreasing CRC cell sensitivity to cuproptosis in a CRC cuproptosis model (Huang T. et al., 2024). In another study, CEBPB enhances autophagy in prostate cancer cells by promoting autophagosome-lysosome fusion and inducing REDD1 expression, whereas its downregulation increases prostate cancer cell sensitivity to bortezomib (Barakat et al., 2016; Wang et al., 2019). NUDT21 overexpression acts as a key regulator of alternative polyadenylation (APA) and exhibits oncogenic properties in prostate cancer, with its unmethylated 3′UTR shortening identified as a novel mechanism underlying enzalutamide resistance (Zhou et al., 2024; Xiao et al., 2023; Lin et al., 2024). Furthermore, downregulation of the RNA methylation regulator YTHDC2 has been associated with cuproptosis resistance in colorectal cancer, with YTHDC2 knockdown attenuating the anti-tumorigenic effects of elesclomol-Cu (Shen et al., 2023; Zhao et al., 2024) (Table 1).

## 3 Cuproptosis agents related to anti-tumor drug resistance

**FIGURE 4:**
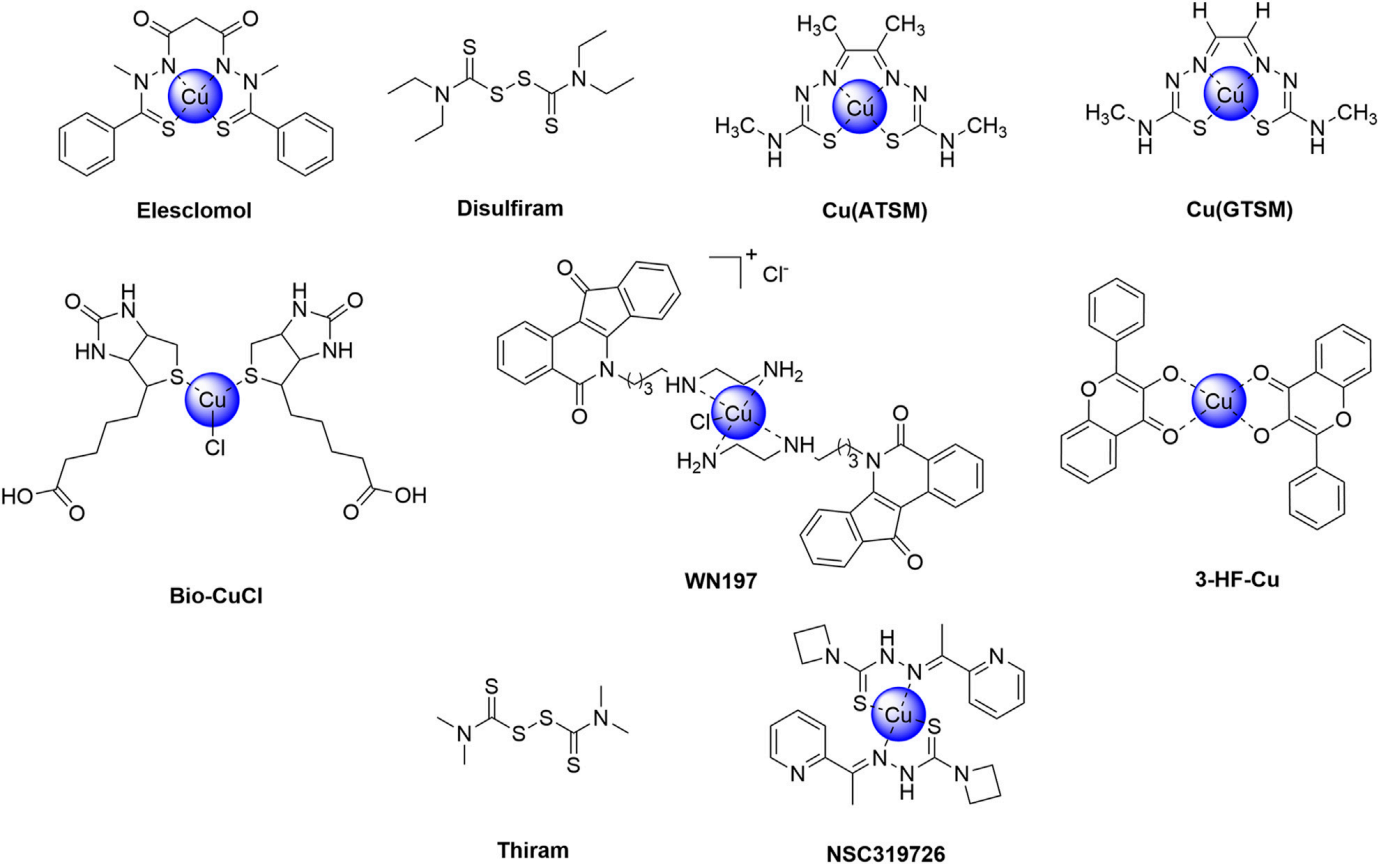
Chemical structures of copper ionophores.

**FIGURE 5:**
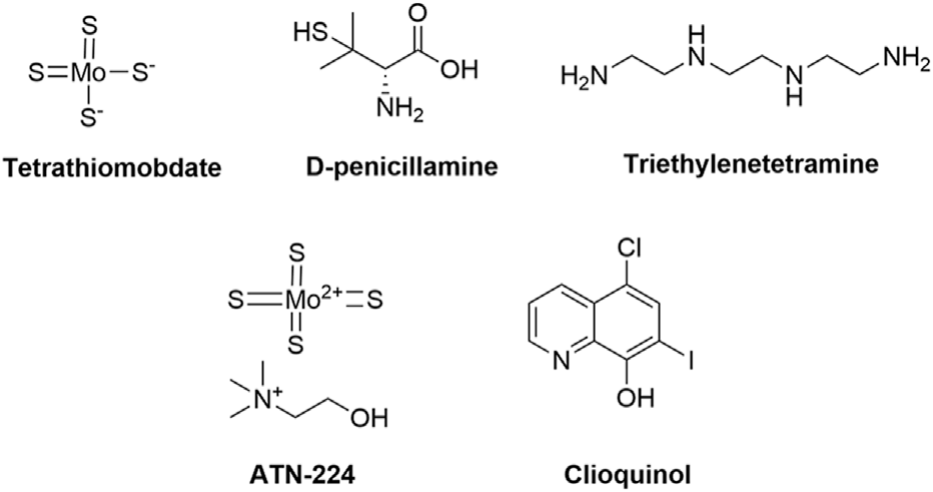
Chemical structures of copper chelators.

Cuproptosis, a newly recognized form of programmed necrosis, is closely associated with tumor cell fate and resistance to anti-tumor drugs. In this process, copper ionophores and copper chelators play crucial and distinct roles, providing novel insights and potential breakthroughs in tumor treatment (Steinbrueck et al., 2020; Li L. et al., 2024; Bian et al., 2023) (Table 2). Copper ionophores specifically facilitate the transport of copper ions into cells, thereby disrupting intracellular copper homeostasis. Studies have demonstrated that copper ionophores exhibit potent sensitization effects in various drug-resistant tumor cell models, both *in vitro* and *in vivo* (Zheng et al., 2022; Feng et al., 2024b). For instance, in platinum-resistant ovarian cancer cell lines, the addition of specific ionophores elevates intracellular copper ion concentrations and activates copper death, thereby inducing significant cell death in previously resistant cells and enhancing chemotherapy sensitivity. In contrast, copper chelators primarily bind to free intracellular copper ions, reducing the concentration of bioactive copper (Ding et al., 2011; Gaál et al., 2018). In the context of tumor drug resistance, cells with abnormal copper metabolism may accumulate excessive copper ions, thereby activating compensatory pathways that facilitate evasion of chemotherapy-induced cell death. Chelators can rectify this overload, inhibit copper ion-mediated abnormal signaling, and restore chemotherapy sensitivity by suppressing related drug resistance proteins, thus reversing drug resistance (Brady et al., 2017). This section provides an overview of current research on copper ionophores and copper chelators in the context of anti-tumor drug resistance. Figures 4, 5 respectively illustrate the structural formulas of common copper ionophores and copper chelators.

**TABLE 2:** Overview of cuproptosis related-agents overcome tumor resistance.

Compound	Tumor type	Drug resistance	Anti-tumor resistance mechanism	Ref
ES/CuCl_2_	PC	Docetaxel	Regulation of the DLAT/mTOR pathway suppressed autophagy and induced G2/M phase arrest, thereby enhancing cellular chemosensitivity to docetaxel	Wen et al. (2023)
DSF/Copper	CC	Cisplatin	DSF/Cu effectively downregulated Bcl-2 and ABCG2 expression, thereby modulating anti-apoptotic and multidrug resistance pathways in both cell lines	Zhang et al. (2024)
DSF/Cisplatin	BC	Cisplatin	DSF potentiates the cytotoxicity of cisplatin by suppressing stemness and overcoming cisplatin resistance in ALDH^+^ stem-like cells	Yang et al. (2019)
DSF/Copper	BC	Paclitaxel	DSF combined with copper induces the generation of ROS and activates downstream apoptosis-related pathways, including c-Jun N-terminal kinase (JNK) and p38 MAPK signaling	Yip et al. (2011)
DSF/Copper	*BRAF^V600E^*-mutated thyroid cancer	Vemurafenib (PLX4032)	DSF/Cu enhances the sensitivity of BRAF-mutant thyroid cancer cells to PLX4032 by inhibiting HER3 and AKT in a ROS-dependent manner, thereby alleviating the feedback activation of the MAPK/ERK and PI3K/AKT pathways	Xie et al. (2023b)
DSF/Copper	AML	Cytarabine, bortezomib	Bortezomib-resistant variants demonstrated cross-resistance to Carfilzomib and MG132 while retaining sensitivity to DSF/Cu^2+^, which selectively induced ubiquitination, apoptosis, and PARP cleavage without affecting chymotrypsin-like proteasome activity	Bista et al. (2017)
DSF/Copper	HCC	Salicylazosulfapyridine, erastin	DSF/Cu inhibited ubiquitination-mediated proteasomal degradation of xCT, leading to its accumulation, and suppressing this compensatory xCT increase enhanced the sensitivity of HCC cells to DSF/Cu, offering a potential strategy to overcome drug resistance and improve tumor therapy	Zhang et al. (2024)
DSF/Copper	Leukemia	Doxorubicin	DSF/Cu complex may re-sensitize HL60/Dox cells to Dox through activating JNK/c-jun as well as inhibiting anti-apoptotic bcl-2 expression	Xu et al. (2011)
DSF/Copper	HCC	Sorafenib	DSF/Cu synergistically enhanced the cytotoxic effects of sorafenib and inhibited tumor growth both *in vitro* and *in vivo* by concurrently suppressing the NRF2 and MAPK signaling pathways	Ren et al. (2021)
DSF/Copper	ESCC	ALDH1-positive	DSF/Cu enhanced radiosensitivity in ALDH1-positive ESCC cells by effectively downregulating the phosphoinositide 3-kinase/Akt signaling pathway	Qian et al. (2023)
DSF/Copper	NSCLC	Normoxia, hypoxia	DSF/Cu selectively enhanced the cytotoxic effects of radiation and chemotherapy on NSCLC cells, particularly under hypoxic conditions, while reducing resistance to these treatments, compared to its effects on HBECs	Falls-Hubert et al. (2020)
DSF/Copper	HCC	Lenvatinib	DSF/copper modulation of FOXO6 enhances the sensitivity of hepatocellular carcinoma to lenvatinib by disrupting choline metabolism	Wu et al. (2024)
DSF/Copper	NSCLC	Taxol	DSF/copper overcomes resistance to microtubule inhibitors in cancer cells by downregulating ALDH2 expression, with copper enhancing the therapeutic efficacy of DSF.	Wang et al. (2018)
DSF/Copper	Glioblastoma	Temozolomide	DSF/copper exhibits limited efficacy in unselected IDH-wildtype glioblastoma and does not appear to notably reinstate sensitivity to temozolomide	Huang et al. (2019a)
CuATSM	DIPGs	Hypoxia	CuATSM selectively induces cytotoxicity in DIPGs through a mechanism involving H_2_O_2_ generation and copper accumulation, exhibiting additive cytotoxic effects when combined with ionizing radiation	Obata et al. (2001)
ES/DSF/enzalutamide	PC	Enzalutamide	enzalutamide significantly enhances copper ionophore-mediated cytotoxicity in enzalutamide-resistant cells	King et al. (2024)
TM/cisplatin	NSCLC	Cisplatin	The combination treatment significantly elevates ROS levels and depletes GSH content, resulting in a marked increase in DNA-bound platinum and enhanced cell apoptosis	Abo El-Maali et al. (2019)
D-penicillamine/platinum	CC	Oxaliplatin	The combined treatment of cisplatin and D-penicillamine intensified the antitumor effect elicited by oxaliplatin in oxaliplatin-resistant S3 xenograft tumors	Yang et al. (2024c)
Clioquinol/Copper	BC	Bodipy prazosin, Ko143	The metal ionophore clioquinol increases BBB abundance of Pgp, an effect associated with increased endothelial cell levels of Cu^2+^	Chen et al. (2015)
ammonium tetrathiomolybdate	Melanoma	BRAF^V600E^ and MEK1/2 Inhibitors	Copper Chelation Inhibits BRAF^V600E^-Driven Melanomagenesis and Counters Resistance to BRAF^V600E^ and MEK1/2 Inhibitors	Brady et al. (2017)

Abbreviation: PC, prostate cancer; BC, breast cancer; AML, acute myeloid leukemia; DIPGs, diffuse intrinsic pontine glioma; HCC, hepatocellular carcinoma; ESCC, esophageal squamous cell carcinoma; NSCLC, non-small cell lung cancer; CC, cervical cancerBcl-2, B-cell lymphoma-2, gene; ABCG2, ATP-binding cassette transporter G2; PARP, poly (ADP-ribose) polymerase; ALDH1, aldehyde dehydrogenase 1; MEK1/2, mitogen-activated protein kinase kinases 1/2; NRF2, nuclear erythroid-related factor-2; Pgp, P-glycoprotein.

### 3.1 Copper ionophores

#### 3.1.1 Elesclomol, ES

ES is a copper ionophore that selectively targets malignant tumors by inducing copper-mediated cell death (Luo et al., 2024). Its mechanism involves promoting the accumulation of copper within cells, thereby triggering a cascade of lethal responses. ES selectively chelates copper ions (Cu^2+^), forming a complex that enters the mitochondria. Through the electron transport chain (ETC.), Cu^2+^ is reduced to Cu^+^, generating ROS in the process (Gao et al., 2023b; Zhang Q. et al., 2022; Nagai et al., 2010). This mechanism relies on the integrity of the functional mitochondrial respiratory chain (Nagai et al., 2010). In cancer cells with active mitochondrial respiration (such as those with high OXPHOS activity), the electron flow through the, ETC., is enhanced, promoting continuous redox cycling of the ES-Cu complex and leading to a significant elevation in ROS levels (Li et al., 2023). The accumulation of ROS induced by ES exceeds the antioxidant defense capabilities of cancer cells (such as the glutathione system), triggering the collapse of mitochondrial membrane potential and ultimately leading to apoptosis (Wen et al., 2023; Tarin et al., 2023; Modica-Napolitano et al., 2019). A critical aspect of ES action is its role in triggering copper-mediated cell death, where excessive copper results in the abnormal aggregation and degradation of cysteine-rich proteins, particularly iron-sulfur cluster proteins (including FDX1), within the mitochondria (Lu X. et al., 2024; Wang W. et al., 2023). This disruption of the mitochondrial respiratory chain and damage to iron-sulfur clusters culminates in cell death. With the emergence and investigation of the concept of cuproptosis, ES has been revisited as a copper ion carrier for clinical trial research (Wang J. et al., 2024).

**FIGURE 6:**
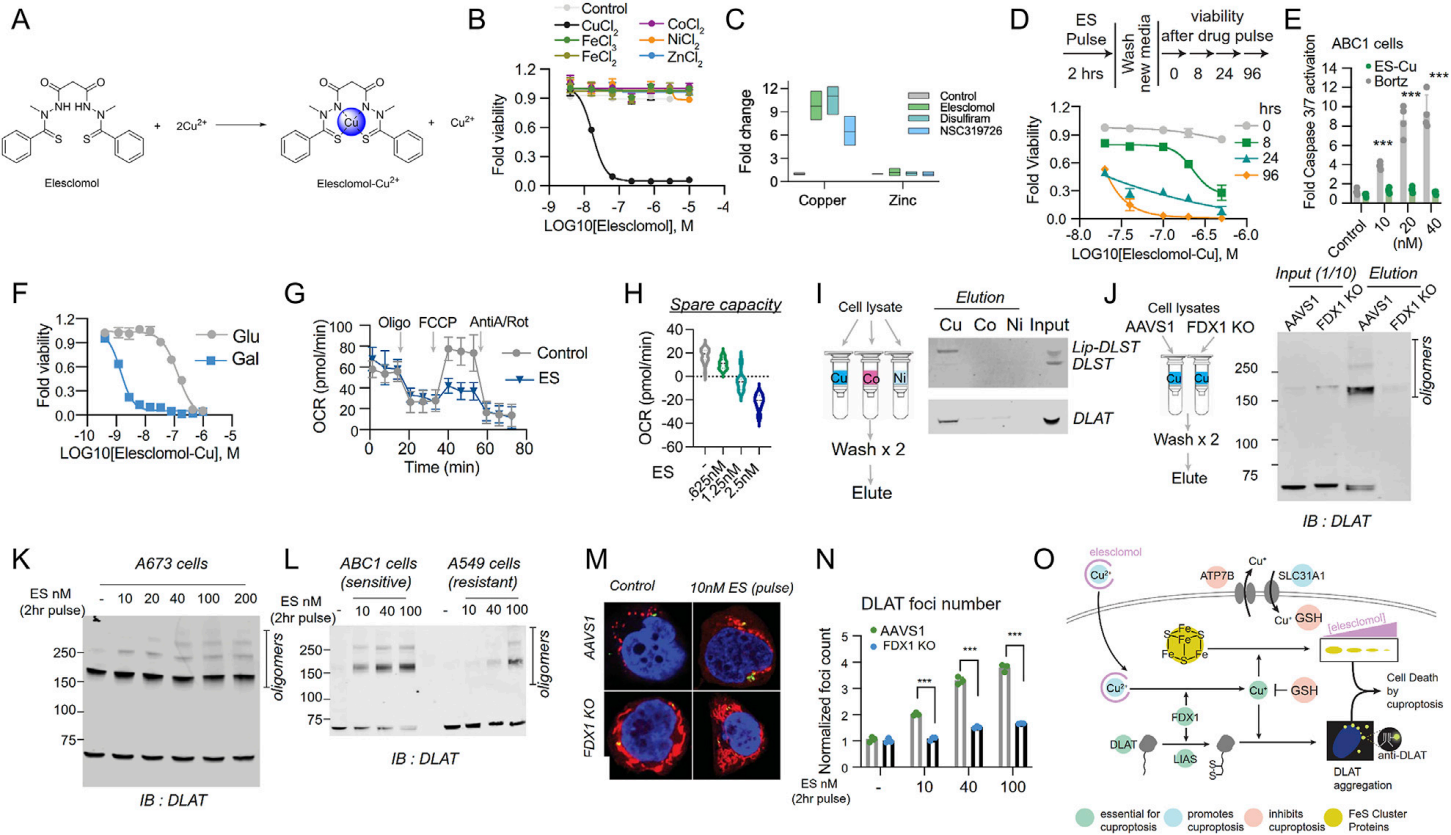
The exploration of the cuproptosis mechanism. **(A)** The chemical structures of elesclomol (ES) and ES-Cu. **(B)** Cell viability (MON) after treatment with ES in the presence or absence of 10 μM of the specified metals was evaluated. **(C)** The levels of copper and zinc were determined by ICP-MS in ABC1 cells following a 2 h pulse treatment with the indicated ionophores or with ES alone, along with supplementation of 1 μM CuCl_2_ in the medium or pre-mixed in a 1:1 ratio with ES. **(D)** The viability of ABC1 cells was examined at the designated times after a pulse treatment of ES-Cu (1:1 ratio) and subsequent growth in fresh media. **(E)** The cleavage of Caspase 3/7 in ABC1 cells 16 h after the indicated treatments (fold change relative to control) was assessed. **(F)** The viability of NCIH2030 cells cultivated in media containing either glucose or galactose and treated with ES-Cu (1:1 ratio) was determined. **(G)** The oxygen consumption rate (OCR) was measured after treating ABC1 cells with 2.5 nM ES (along with 1 μM CuCl_2_ in the media) for 16 h, both before (basal) and after the addition of oligomycin (ATP-linked), the uncoupler FCCP (maximal), or the electron transport inhibitor antimycin A/rotenone (baseline). **(H)** The oxygen consumption rate (OCR) in ABC1 cells was detected following a 16 h treatment with 2.5 nM ES (+1 μM CuCl_2_ in media) or the specified concentrations and before and after the addition of oligomycin (ATP-linked), the uncoupler FCCP (maximal) or the electron transport inhibitor antimycin A - baseline. **(I)** The binding of the designated proteins to copper (Cu), cobalt (Co.), and nickel (Ni) was evaluated through immunoblot analysis of the eluted proteins from the metal-loaded resins as indicated. **(J)** Copper binding was assessed by loading cell lysates from either ABC1 AAVS1 or FDX1 KO cells onto the copper-loaded resin, followed by washing and analysis of the eluted proteins. The input and eluted proteins are presented. IB stands for immunoblot. **(K)** The protein content was analyzed in A673 cells that underwent a 2 h pulse treatment with the indicated concentrations of elesclomol. **(L)** The protein content was analyzed in ABC1 and A549 cells that were pulse-treated with the indicated concentrations of ES. **(M, N)**. Immunofluorescence imaging (green, DLAT–; red, Mitotracker; blue, Hoechst; white, phalloidin). The foci were segmented and quantified under each condition. **(O)** Schematic illustration of the mechanisms that drive copper-induced cell death. (Reprinted with permission from Ref. (Kahlson and Dixon, 2022). Copyright ^©^ 2022 by the authors, some rights reserved; exclusive licensee American Association for the Advancement of Science).

In the exploration of the cuproptosis mechanism, ES, characterized as a potent copper ionophore, has emerged as a principal research focus, with the mechanism underlying copper ion-dependent cell death now elucidated (Kahlson and Dixon, 2022; Guo et al., 2023; Wang H. et al., 2024). As copper in the cell culture medium is derived from serum, cells exhibit resistance to ES in a serum-free environment. However, supplementation with copper at a 1:1 ratio fully restores ES sensitivity (Figure 6A) (Kahlson and Dixon, 2022). Copper supplementation renders cells sensitive to treatment with six structurally distinct copper ionophores. In contrast, supplementation with metals such as iron, cobalt, zinc, and nickel fail to enhance cell death (Figure 6B). Treatment with potent copper ionophores (elesclomol, disulfiram, NSC319726) via pulsed exposure for 2 h resulted in an approximately 5- to 10-fold increase in intracellular copper levels, while zinc levels remained unaltered (Figure 6C). Pulsed treatment with the copper ionophore elesclomol at a concentration as low as 40 nM for 2 h resulted in a 15- to 60-fold increase in intracellular copper levels and induced cell death after 24 h (Figure 6D). Although previous reports have indicated that elesclomol induces ROS-dependent apoptosis, the current study reveals that its induction of cell death does not involve the cleavage or activation of caspase 3, a hallmark of apoptosis (Figure 6E). Cells dependent on mitochondrial respiration exhibit nearly a 1000-fold higher sensitivity to copper ionophores compared to glycolytic cells, providing a crucial clue for the pathway mediating copper ionophore-induced cell death (Figure 6F) (Pérez-Henarejos et al., 2015). Furthermore, the mitochondrial uncoupling agent FCCP has no impact on copper toxicity, suggesting that copper-induced cell death requires mitochondrial respiration rather than ATP production (Figure 6G). Moreover, treatment with copper ionophores does not significantly reduce basal or ATP-linked respiration but markedly diminishes spare respiratory capacity (Figure 6H) (Kahlson and Dixon, 2022).

Researchers have also identified that copper directly binds to lipoylated DLAT and induces its oligomerization (Smirnova et al., 2018; Gateau and Delangle, 2014). Purified DLAT and DLST from cell lysates bind to copper resin but do not bind to cobalt or nickel resins (Figure 6I). Interestingly, copper binding to lipoylated proteins in the TCA cycle promotes lipoylated-dependent DLAT oligomerization, detectable by non-denaturing gel electrophoresis (Figures 6J,K). Treatment of elesclomol-sensitive cells enhances the levels of DLAT oligomers and insoluble DLAT. In contrast, treatment of elesclomol-insensitive cell lines or FDX1 knockout (FDX1 KO) cells results in DLAT oligomerization only at substantially higher concentrations (Figure 6L) (Kahlson and Dixon, 2022). Copper binding to lipoylated proteins in the TCA cycle leads to the acquisition of toxic functions. Immunofluorescence experiments demonstrate that short-pulse treatment with elesclomol significantly induces DLAT lesions, which are diminished in FDX1 KO and lipoylation-deficient cells, suggesting that the enhancement of the toxic function of lipoylated proteins following copper ionophore exposure is at least partially mediated by their abnormal oligomerization (Figures 6M,N). The aforementioned studies confirm that copper-dependent cell death originates from the direct binding of copper to lipoylated components in the TCA cycle, resulting in the aggregation of lipoylated proteins, the loss of iron-sulfur cluster proteins, induction of proteotoxic stress, and ultimately cell death (Figure 6O) (Kahlson and Dixon, 2022). Furthermore, it presents a novel action pathway for overcoming tumor drug resistance mechanisms.

#### 3.1.2 Disulfiram, DSF

Disulfiram is a medication traditionally employed in the treatment of alcohol dependence (Lu et al., 2022). Recent studies have demonstrated its potential as an anti-cancer agent, particularly through the induction of copper-mediated cell death in malignant tumors (Skrott et al., 2017). Its anti-tumor activity is primarily attributed to the regulation of copper levels and the induction of intracellular oxidative stress. Upon entering the body, disulfiram is rapidly converted to its active metabolite, diethyldithiocarbamate (DTC), a potent copper chelator that forms stable complexes with copper, thereby significantly increasing intracellular copper concentrations (Xu et al., 2021; Li et al., 2020). This copper-DTC complex enhances the bioavailability of copper within tumor cells, thereby promoting its toxic effects (Li et al., 2020; Wang et al., 2021). Elevated copper levels result in the generation of excessive ROS, leading to heightened oxidative stress, which is particularly detrimental to tumor cells due to their higher metabolic activity and sensitivity to oxidative stress (Yang et al., 2019; Yip et al., 2011; Xie et al., 2023b). Furthermore, disulfiram’s binding to copper inhibits mitochondrial respiratory chain activity, thereby blocking ATP production and causing energy depletion, which accelerates apoptosis in tumor cells (Yang L. et al., 2024). The combination of DSF and copper may also overcome resistance to conventional therapies, such as chemotherapy and radiotherapy, in specific tumors (acute myeloid leukemia (Bista et al., 2017; Xu et al., 2011), hepatocellular carcinoma (Zhang et al., 2024; Ren et al., 2021; Wu et al., 2024), esophageal squamous cell carcinoma (Qian et al., 2023), NSCLC (Falls-Hubert et al., 2020; Wang et al., 2018), and glioblastoma (Huang J. et al., 2019)) due to its unique mechanism of action and regulation of copper metabolism. In addition, DSF can inhibit the ATPase activity of ATP-binding cassette (ABC) transporters, thereby blocking the efflux of chemotherapy drugs such as paclitaxel and doxorubicin, ultimately increasing the intracellular drug concentration. Experimental data suggest that DSF interferes with the conformation of drug binding sites through covalent modification of the sulfhydryl groups of transporters (Sauna et al., 2004). By reversing the drug resistance mediated by the ABC family, DSF can restore the cytotoxicity of paclitaxel in drug-resistant cancer cells overexpressing P-gp, reducing IC_50_ value by approximately 10-fold. This effect has been validated in breast cancer and ovarian cancer models (Huo et al., 2017; Swetha et al., 2023). Moreover, combining disulfiram with other anti-cancer agents, including platinum-based drugs, has been shown to enhance therapeutic efficacy and reduce the risk of tumor recurrence.

**FIGURE 7:**
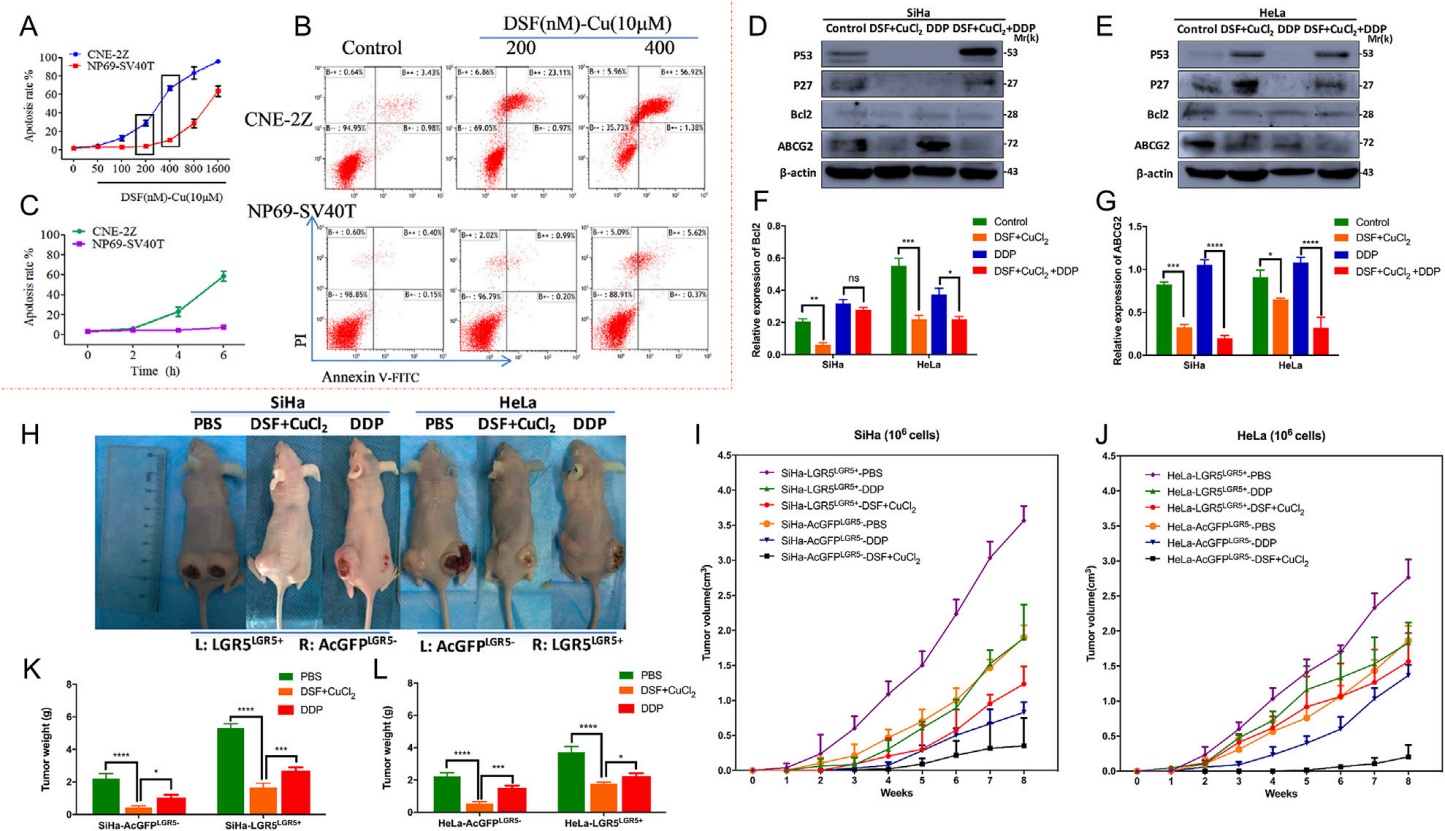
Study on the mechanism of DSF/Cu in anti-tumor drug resistance. **(A)** DSF/Cu complex cytotoxicity was determined using the dose-dependent curves and in the human nasopharyngeal carcinoma CNE-2Z and normal nasopharyngeal epithelial cells NP69-SV40T. Different concentrations of 50, 100, 200, 400, 800, and 1,600 nM DSF chelated with 10 μM CuCl_2_ for 6 h. **(B)** The apoptosis rate of DSF/Cu induced CNE-2Zand NP69-SV40T cells was evaluated using Annexin V-FITC/PI apoptosis detection kit. **(C)** DSF/Cu complex cytotoxicity was determined using the time-dependent curves in CNE-2Z and NP69-SV40T cells. The time-dependent curve was treated with DSF/Cu 400 nM at 2 h, 4 h, and 6 h (Reprinted with permission from Ref. (Yang et al., 2017). Copyright 2017 Elsevier). **(D)** DSF/Cu complex has been demonstrated to trigger apoptosis in cervical cancer cell lines (Siha and Hela cells). Western blotting was employed to detect the expression of apoptosis-related proteins, including p53, p27, and Bcl2, as well as the resistance protein ABCG2. **(E, F)** The ImageJ software was utilized to analyze the relative expression levels of Bcl2 and ABCG2 **(F, G)**. **(H)** *In vivo*, xenograft tumor experiments (using female BALB/c-nude mice) have demonstrated that the DSF/Cu and cisplatin treatment groups can inhibit LGR5-positive cervical cancer cells. **(I, J)** Detection of the volumes of xenograft tumors of LGR5-positive and -negative cervical cancer cells in nude mice under different administration regimens was also carried out. (K and L) Changes in mice body weight during treatments. (Reprinted with permission from Ref. (Cao et al., 2022). Copyright 2022 by the authors).

Research on the anti-tumor properties of the DSF/Cu complex has demonstrated its selective cytotoxicity, exhibiting potent anti-tumor effects on malignant cells while displaying minimal toxicity to normal cells. In a study evaluating the anti-proliferative activity and apoptotic effects of the DSF/Cu complex on nasopharyngeal carcinoma CNE-2Z cells and normal nasopharyngeal epithelial NP69-SV40T cells, the EC_50_ values for DSF were 295.5 nM and 1,354 nM, respectively. When treated with 400 μM DSF/Cu (10 μM), the apoptosis rates in CNE-2Z and NP69-SV40T cells were 58.3% and 5.99%, respectively (Figures 7A,B) (Yang et al., 2017). In contrast to almost no change in the NP69-SV40T cell group, the apoptosis rate of CNE-2Z cells in the DSF/Cu (400 nM)-treated group exhibited a time-dependent increase (Figure 7C). In another investigation, the combination of DSF/Cu and cisplatin was evaluated for its ability to overcome drug resistance in cervical cancer cells (SiHa and HeLa) both *in vitro* and *in vivo* (Cao et al., 2022). Drug resistance genes, including LGR5 and related proteins, were evaluated. SiHa and HeLa cells were exposed to 0.1 μM DSF and 0.01 μM CuCl_2_ (DSF/Cu complex), 10 μM cisplatin (DDP), or a combination of DSF/Cu and DDP (DSF/Cu/DDP) for 48 h. The findings indicated that DSF/Cu/DDP significantly upregulated p53 and p27 protein expression in both cell lines (Figures 7D–G). Notably, DSF/Cu alone induced p53 and p27 expression in HeLa cells. Furthermore, DSF/Cu suppressed the expression of the anti-apoptotic protein Bcl-2 and the multidrug resistance gene ABCG2 in both cell lines. The ABCG2 expression in the DDP-treated group was markedly higher than in the DSF/Cu/DDP-treated group (Figures 7D–G). *In vivo* experiments using xenograft tumor models in nude mice investigated whether DSF/Cu could target LGR5-positive cervical cancer stem-like cells. A modified transplantation model of LGR5-positive and LGR5-negative SiHa and HeLa cells in BALB/c nude mice was utilized (Figure 7H). Tumors derived from LGR5-positive cells exhibited larger volumes and more rapid growth than those from LGR5-negative cells (Figures 7I,J). However, in the DSF/Cu-treated group, tumor growth was significantly slower, with smaller tumor volumes compared to the DDP and PBS control groups. At the end of treatment, tumor weights in the DSF/Cu group were notably lower than those in the DDP and PBS groups (Figures 7K,L) (Cao et al., 2022). DSF exerts substantial cytotoxic effects on malignant tumors by binding to copper and activating the cuproptosis mechanism, offering new avenues for anti-tumor treatment and promising opportunities for repurposing this traditional drug. Additionally, thiram is being studied as a structural analog of DSF in the anti-tumor direction.

#### 3.1.3 CuATSM and CuGTSM

CuATSM (copper (II) diacetyl-bis(4-methyl-3-thiosemicarbazone)) and CuGTSM (copper (II) glyoxal bis(4-methyl-3-thiosemicarbazone)) are ionophores that have attracted considerable attention due to their ability to induce copper-mediated cell death, a mechanism specifically targeting malignant tumors (Andres et al., 2020; Cater et al., 2013). These compounds exert cytotoxic effects through several key mechanisms by selectively delivering copper to cancer cells. CuATSM and CuGTSM possess unique properties that facilitate the selective delivery of copper to tumor regions characterized by hypoxia, or low oxygen levels. Tumors often exhibit hypoxic microenvironments as a result of rapid growth and inadequate blood supply. Under these conditions, CuATSM and CuGTSM undergo reduction, resulting in the preferential release of copper into tumor cells. Once inside cancer cells, the released copper catalyzes ROS production, producing oxidative stress that damages essential cellular components such as DNA, proteins, and lipids, ultimately causing cell death (Xie and Wei, 2022). Additionally, copper disrupts mitochondrial function by interfering with the electron transport chain, resulting in a collapse of the mitochondrial membrane potential, impaired ATP production, and the initiation of apoptotic pathways (Obata et al., 2001). The preferential activity of CuATSM and CuGTSM under hypoxic conditions makes them particularly effective for treating tumors resistant to other therapies due to the hypoxic nature of these tumors. Diffuse intrinsic pontine glioma (DIPG), a primary brainstem tumor occurring in children is characterized by its high aggressiveness and resistance to treatment (Roig-Carles et al., 2021; King et al., 2024). The current study demonstrates that CuATSM exerts its effect dose-dependent (1–3 μM), significantly reducing the clonal cell survival rate of SU-DIPG50 and SU-DIPG36 cells. Notably, it has no substantial impact on the survival of normal human astrocytes (NHA). Moreover, CuATSM demonstrates selective cytotoxicity towards DIPG through mechanisms intricately associated with generating H_2_O_2_ and copper involvement. Additionally, it exhibits an additive cytotoxic effect when combined with ionizing radiation, further highlighting its potential role in treatment strategies for DIPG (King et al., 2024). In contrast to CuATSM, CuGTSM targets mitochondria and releases copper within these organelles, compromising mitochondrial integrity and energy production by disrupting mitochondrial enzymes and proteins (Holland et al., 2009; Ji et al., 2023). Copper release within the mitochondria can induce apoptosis by promoting cytochrome c release and activating caspases. However, the clinical application of CuATSM and CuGTSM requires careful consideration of their delivery mechanisms, potential toxicity, and the specific tumor microenvironment.

#### 3.1.4 Other copper ionophores

Topoisomerase inhibitors are frequently considered potential options for chemotherapy, as they induce the accumulation of DNA breaks and hinder DNA damage repair, ultimately leading to tumor cell death (Madkour et al., 2023; Conilh et al., 2021). WN197 has been explored as a promising topoisomerase I inhibitor against tumor drug resistance. Studies have shown that it also inhibits topoisomerase IIα and IIβ and exhibits DNA intercalation properties. Furthermore, in adenocarcinoma, WN197 exerts specific cytotoxic effects on 3 cell lines (MDA-MB-231, HeLa, and HT-29) at low concentrations (with an IC_50_ value below 0.5 µM), significantly lower than the IC_50_ (1.08 µM) of the human normal breast epithelial cell line MCF-10A. Additionally, a significant upregulation of the DNA damage marker protein γH2AX was observed in the WN197-treated groups (Molinaro et al., 2022). Activating transcription factor 3 (ATF-3), a member of the ATF/CREB transcription factor family, primarily promotes the co-selection of cancer cells by the host and facilitates invasion and metastasis by regulating the host response (Hasim et al., 2018; Chan et al., 2012). Research has demonstrated that the combination of 3-hydroxyflavone (3-HF) and silica nanoparticles downregulates ATF-3 expression in breast cancer cells with bone metastasis. Simultaneously, this combination increases the expression of cytochrome C and caspase 9 while inhibiting the growth of MCF-7 and MDA-MB-231 cancer cells (Abo El-Maali et al., 2019) (Table 2).

### 3.2 Copper chelators

#### 3.2.1 Tetrathiomolybdate, TM

TM exerts its anti-tumor effects primarily through copper chelation, thereby reducing copper availability in tumor cells and influencing various aspects of cancer progression (Gao et al., 2024; Gao F. et al., 2023). Copper serves as a critical cofactor for several enzymes and growth factors involved in angiogenesis, including VEGF and FGF, which tumors exploit to form new blood vessels that supply vital nutrients and oxygen (Heuberger et al., 2019). By binding to copper, TM depletes both intracellular and extracellular copper levels, inhibiting tumor angiogenesis and limiting nutrient supply, thereby suppressing tumor growth (Gao et al., 2024). TM also inhibits copper-dependent enzymes, such as superoxide dismutase (SOD1) and lysyl oxidase (LOX), which play crucial roles in regulating oxidative stress and remodeling the extracellular matrix—key processes in tumor development and metastasis. By reducing intracellular copper levels, TM diminishes the activity of these enzymes (Morisawa et al., 2018; Chan et al., 2017). For example, inhibiting LOX can decrease tumor cell invasiveness, thereby slowing metastasis. Moreover, copper promotes tumor cell proliferation through signaling pathways such as MAPK and NF-κB. In a mouse model of breast cancer, TM markedly decreased the density of new blood vessels within the tumor, suppressing tumor growth and metastasis. Experimental results indicate that tumor volume decreased by approximately 40% after TM treatment (Ren et al., 2024). TM mitigates the activity of these pathways, promoting apoptosis in copper-dependent tumor cells, including those in breast and liver cancers.

With regard to immune regulation, TM enhances anti-tumor immune responses by reducing the number of regulatory T cells (Tregs), enabling immune cells, such as T lymphocytes and natural killer (NK) cells, to more effectively target tumor cells (Yang J. et al., 2024). Furthermore, TM can counteract chemotherapy resistance, which some cancer cells develop through increased expression of copper transport proteins (e.g., ATP7A and ATP7B). By depleting copper levels, TM diminishes the activity of these transporters, thereby restoring tumor cell sensitivity to chemotherapeutic agents and enhancing traditional cancer treatments (Ishida et al., 2010). Preclinical studies have demonstrated TM’s capacity to inhibit the growth of various tumors, including breast cancer, liver cancer, and glioma, in animal models, further confirming its anti-angiogenic properties. Early-phase clinical trials have suggested TM’s safety in patients with metastatic breast cancer and other solid tumors; however, larger studies are required to validate its efficacy. TM represents a promising multifaceted approach to cancer therapy, involving mechanisms such as angiogenesis inhibition, interference with copper-dependent enzyme functions, apoptosis induction, immune modulation, and overcoming chemotherapy resistance. While preclinical results are promising, further large-scale clinical investigations are required to ascertain TM’s true efficacy in cancer treatment (Chisholm et al., 2016). In breast cancer therapy, TM has been demonstrated to enhance the effects of cisplatin. Studies suggest that combining cisplatin with TM delays tumor growth and reduces cancer stem cell accumulation and cell proliferation in breast cancer models (Chisholm et al., 2016; Kim et al., 2015). This combination treatment significantly decreases proliferation and migration in H1299 and A549 lung cancer cells, elevates ROS levels, and reduces GSH content, resulting in enhanced DNA-Pt adduct formation and increased apoptosis rates (Li et al., 2022).

#### 3.2.2 D-penicillamine

D-penicillamine is a well-established copper chelator, primarily used in the treatment of Wilson disease, and recent studies have underscored its potential anti-tumor activity (Tang et al., 2022; Antos et al., 2023). Similar to TM, D-penicillamine reduces the availability of free copper in cells by binding to it, thereby inhibiting factors such as VEGF and FGF. This inhibition impedes the formation of new blood vessels that supply oxygen and nutrients to tumors. Moreover, D-penicillamine induces oxidative stress in tumor cells by chelating copper and generating ROS, which leads to cellular damage and promotes apoptosis. Elevated ROS levels can disrupt proteins, lipids, and DNA in tumor cells, thereby resulting in cell death and inhibiting tumor growth (Gupte and Mumper, 2007). Evidence indicates that copper chelation may also modify the tumor microenvironment, thus improving the immune response against tumors (Mammoto et al., 2013). By reducing copper-dependent factors that facilitate immune evasion, D-penicillamine may enhance the immune system’s ability to recognize and eliminate cancer cells.

Furthermore, some cancer cells develop resistance to chemotherapy through copper transport mechanisms. By depleting copper levels, D-penicillamine may reverse this resistance and restore the efficacy of certain chemotherapeutic agents. It upregulates the copper influx transporter hCtr1 through the activation of Sp1, which subsequently induces the translocation of p53 from the nucleus to the cytoplasm (Chen et al., 2015). This process results in p53 degradation via ubiquitination and inhibits the expression of the copper efflux transporter ATP7A. The combination of cisplatin and D-penicillamine has been demonstrated to enhance the anti-tumor effects of oxaliplatin in oxaliplatin-resistant S3 xenograft tumors, particularly in cases with downregulated hCtr1 expression and overexpressed ATP7A (Koraishy et al., 2013). Despite its potential, the clinical limitations of D-penicillamine primarily stem from its nonspecific metal chelating properties (resulting in nephrotoxicity and hepatotoxicity), immunogenicity (leading to Goodpasture’s syndrome-like manifestations or systemic lupus erythematosus (SLE)-like symptoms in approximately 5%–10% of patients), and the cumulative toxicity of its metabolites. To balance efficacy and safety, individualized dose adjustments, regular monitoring (including urine protein, liver, and kidney function tests), and avoidance of high-risk combination regimens are necessary (Koraishy et al., 2013).

#### 3.2.3 Triethylenetetramine, TETA

TETA (or Trientine) is a copper chelator primarily used for the treatment of Wilson disease by effectively reducing copper levels in the body. Recent studies suggest that TETA also regulates copper metabolism and exhibits potential anti-tumor effects, particularly in tumors with abnormal copper levels (Yoshii et al., 2001). The anti-tumor mechanisms of TETA are multifaceted. It reduces intracellular copper levels in tumor cells, thereby inhibiting their growth and proliferation by depriving them of essential copper, which disrupts critical biological processes (Yoshii et al., 2001). Copper is implicated in tumorigenesis through signaling pathways such as receptor tyrosine kinases (RTKs), autophagy, and Notch signaling. By chelating copper, TETA modulates these pathways, thereby further suppressing tumor proliferation and invasion (Kazakova et al., 2020; Zhao et al., 2022).

Furthermore, TETA has been investigated in combination with chemotherapy agents to augment anti-tumor effects. Research indicates that combining TETA with carboplatin and pegylated liposomal doxorubicin (PLD) is safe and effective for patients with epithelial ovarian cancer (EOC), fallopian tube cancer, and peritoneal cancer, particularly those with disease progression during or shortly after platinum-based chemotherapy (Huang Y. F. et al., 2019). Pharmacokinetic studies revealed enhanced absorption and more rapid elimination of TETA in these patients, with clinical benefit rates of 33.3% in platinum-resistant and 50.0% in partially platinum-sensitive groups (Huang Y. F. et al., 2019). Moreover, TETA promotes ROS generation, thereby inducing apoptosis in tumor cells (Yin et al., 2016). When used in conjunction with immunotherapy agents such as PD-L1 inhibitors, TETA enhances anti-tumor immune responses via copper-dependent cell death. TETA significantly contributes to anti-tumor therapy as a copper chelator, functioning through the reduction of intracellular copper levels, modulation of signaling pathways, enhancement of chemotherapy efficacy, induction of apoptosis, and improvement of anti-tumor immune responses when combined with immunotherapy. Future research should further elucidate the anti-tumor mechanisms of TETA and optimize its clinical applications, thereby offering new strategies for cancer treatment.

#### 3.2.4 Clioquinol, CQ

CQ is a copper chelator initially employed in the treatment of skin infections and amoebic dysentery, but it has recently attracted attention for its potential as an anti-tumor agent (Bareggi and Cornelli, 2012; Lu et al., 2018). In the tumor microenvironment, CQ functions both as a copper ion chelator to deplete intracellular copper ions and to inhibit the activity of copper - dependent enzymes such as SOD1, leading to ROS accumulation and mitochondrial dysfunction, and as a direct inhibitor of 26S proteasome activity, impeding the degradation of ubiquitinated proteins and resulting in the abnormal accumulation of misfolded proteins within cells (Sun H. et al., 2025). Its anti-tumor mechanism primarily entails the regulation of metal ions, particularly copper and zinc (Yap et al., 2021; Katsuyama et al., 2021). Regulation of breast cancer resistance protein (BCRP), an efflux transporter at the blood-brain barrier, may influence the levels of drugs and endogenous substrates in the brain (Yap et al., 2021). Previous studies have demonstrated that the metal chelator CQ can augment the abundance of P-glycoprotein (P-gp) in the blood-brain barrier, which is associated with elevated Cu^2+^ levels in endothelial cells (Yap et al., 2021; Schimmer, 2011). Consequently, researchers sought to determine whether CQ could modulate the abundance and function of BCRP in human cerebral microvascular endothelial cells (hCMEC/D3). The hCMEC/D3 cells were exposed to CQ, Zn^2+^, and Cu^2+^ (collectively referred to as CZC at 0.5 mM, 0.5 mM, and 0.1 mM, respectively) for 24 h. Subsequently, WB and qPCR data were employed to detect the mRNA and protein abundances of BCRP. Following optimization studies, chlorpromazine (BP) and Ko143 were evaluated for their specificity as a BCRP substrate and inhibitor, respectively, and the impact of CZC on BP uptake was assessed. Results indicated that CZC did not increase BCRP mRNA expression but resulted in a 1.8 ± 0.1-fold increase in BCRP abundance, correlating with a 68.1% ± 3.3% reduction in BP accumulation within hCMEC/D3 cells (Yap et al., 2021). Furthermore, it promotes the generation of ROS, inducing oxidative stress that damages DNA, proteins, and lipids in cancer cells, ultimately triggering apoptosis. This effect is particularly pronounced in tumors exhibiting abnormal copper metabolism. CQ also inhibits proteasome activity, a crucial cellular process responsible for degrading damaged proteins. This inhibition leads to the accumulation of damaged proteins, further inducing apoptosis, particularly in cancer cells (Yu et al., 2010). Studies have shown that CQ (20–50 µM) significantly increases intracellular copper levels within 24 h, whereas chloroquine (50 µM) induces the oxidation and subsequent inactivation of ATOX1, thereby impairing copper transport. Moreover, CQ chelates copper to suppress the NF-κB signaling pathway, which regulates inflammation, cell proliferation, and anti-apoptotic processes (Baum and Ng, 2004). Through NF-κB inhibition, CQ enhances apoptosis in cancer cells and effectively suppresses tumor progression. Its anti-tumor efficacy has demonstrated promising results across various cancer models, including prostate, breast, and brain cancers.

### 3.3 Cuproptosis nanodelivery system related to tumor drug resistance

The nanodelivery system holds significant potential in the modulation of cuproptosis. Through the application of various approaches, including copper ion chelators, copper ionophores, mitochondrially targeted drugs, gene therapy vectors, and the development of combined treatment platforms, nanodrug delivery can modulate copper ion homeostasis, mitochondrial metabolic disorders, and associated signaling pathways in the context of cuproptosis (Chen et al., 2022; Kahlson and Dixon, 2022). By modulating the cuproptosis process through various mechanisms, nanodrugs offer novel strategies for treating diverse diseases, particularly those linked to copper ion homeostasis dysregulation, such as tumors and neurodegenerative diseases. This review primarily focuses on summarizing the latest advancements in cuproptosis nanodelivery for tumor treatment, particularly in overcoming tumor drug resistance.

**FIGURE 8:**
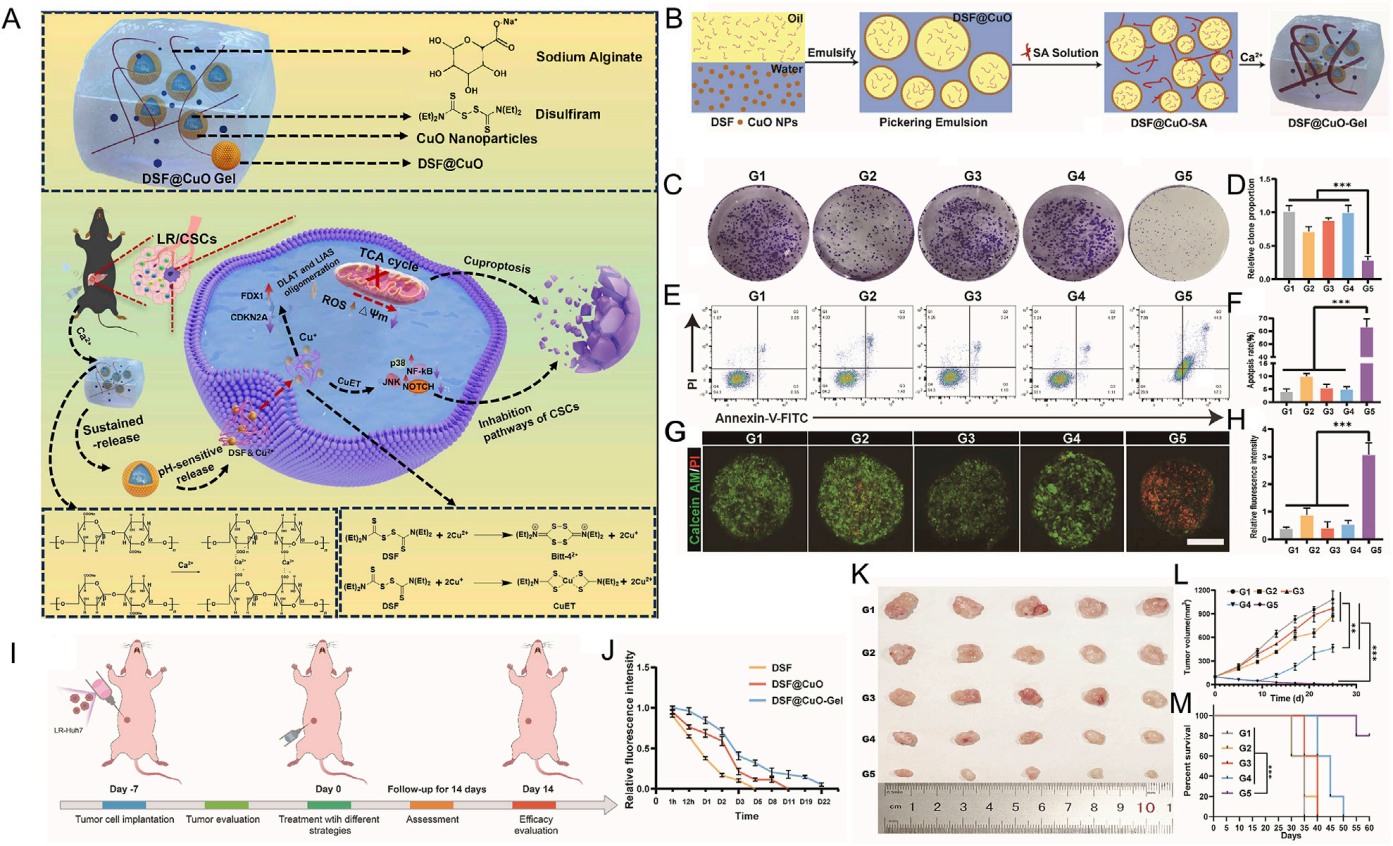
The preparation of DSF@CuO Gel hydrogel injection and evaluation bioactivity in LenR carcinoma. **(A)** Schematic representation of DSF@CuO Gel hydrogel injection for the treatment of lenvatinib-resistant hepatocellular carcinoma (LenR HCC). **(B)** Schematic depiction of the DSF@CuO Gel synthesis process. **(C–F)** Effects of various treatment groups on colony formation assays and apoptosis induction in LenR-Huh7 cells. **(G–H)** Evaluation of the cytotoxic effects of the treatment groups on tumor spheroids derived from LenR-Huh7 cells. Treatment groups include G1 (corn oil), G2 (DSF), G3 (CuO NPs), G4 (Len@CuO), and G5 (DSF@CuO). **(I)** DSF@CuO Gel has been demonstrated to possess the ability to inhibit tumor growth within LenR mouse models. A schematic diagram is provided to illustrate the treatment procedure in detail. **(J)** Quantitative evaluation was conducted to assess the sustained release effects of DSF, DSF@CuO, and DSF@CuO Gel in mouse models. **(K)** Photographs were taken of the dissected mouse tumor tissues after the completion of the treatment. **(L)** The *in vivo* tumor volume curve was plotted to reflect the changes during the treatment process for each group of mice. **(M)** Survival analysis was carried out on mice across different treatment groups. (Reprinted with permission from Ref. (Li X. et al., 2024). Copyright 2021 by the authors).

Lenvatinib resistance (LenR) in hepatocellular carcinoma (HCC) has emerged as a significant and challenging obstacle, contributing to the persistently high global cancer-related mortality rate (Li X. et al., 2024). LenR in HCC primarily arises from the enhanced stemness of cancer cells, which distinguishes it from traditional chemoresistance mechanisms (Li X. et al., 2024; Llovet et al., 2022; Eun et al., 2023). The copper-induced cell death (cuproptosis) mechanism of DSF/Cu holds promise for overcoming LenR in HCC. However, DSF exhibits poor water solubility, and copper ions are linked to systemic toxicity concerns during widespread application. In a specific study, researchers employed a method of co-encapsulating DSF and CuO nanoparticles (NPs) to form an oil-in-water Pickering emulsion (DSF@CuO), thereby increasing the concentrations of DSF and copper ions within the tumor microenvironment (TME) (Figure 8A). Subsequently, DSF@CuO was combined with sodium alginate (SA) to form a DSF@CuO-SA solution, which could undergo *in situ* gelation in the presence of Ca^2+^ within the TME, resulting in the formation of DSF@CuO gel (Figure 8B). This approach not only enhanced the stability of the Pickering emulsion but also ensured the sustained release of DSF and copper ions (Li X. et al., 2024).

In the *in vitro* biological evaluation, the experiment was divided into five groups: G1 (Corn Oil), G2 (DSF), G3 (CuO NPs), G4 (Len@CuO), and G5 (DSF@CuO). The colony formation assay further supported the anti-proliferative effect of DSF@CuO, consistent with the CCK8 assay results, suggesting its anti-tumor potential (Figures 8C,D). The apoptosis rate was measured using Annexin V-FITC and PI double staining. The results revealed that the apoptosis rate of LenR Huh7 cells treated with DSF@CuO was significantly higher compared to the control group (Figures 8E,F). Furthermore, data from the 3D tumor spheroid model demonstrated that the number of dead cells (in red) was greatest, further confirming its therapeutic potential within the tumor microenvironment (Figures 8G,H). Collectively, these results suggest that DSF@CuO exerts a potent cytotoxic effect on LenR HCC cells *in vitro*. Subsequently, a subcutaneous model of LenR HCC was established in nude mice for *in vivo* evaluation. The data revealed that the CuO gel degraded in approximately 22 days, outperforming the 11-day degradation of DSF@CuO emulsion and the 5-day degradation of single-injected DSF, thereby demonstrating excellent drug retention and sustained release properties (Figures 8I,J). Twenty-five mice were randomly assigned to five groups (SA, DSF, CuO NPs, DSF@CuO, and DSF@CuO gel) for evaluation of treatment efficacy. The tumor volume in the DSF@CuO treatment group initially decreased and then increased, while the tumor volume in the DSF@CuO gel group continuously decreased, suggesting that the sustained-release effect of the hydrogel improved treatment efficacy (Figures 8K-M) (Li X. et al., 2024).

**FIGURE 9:**
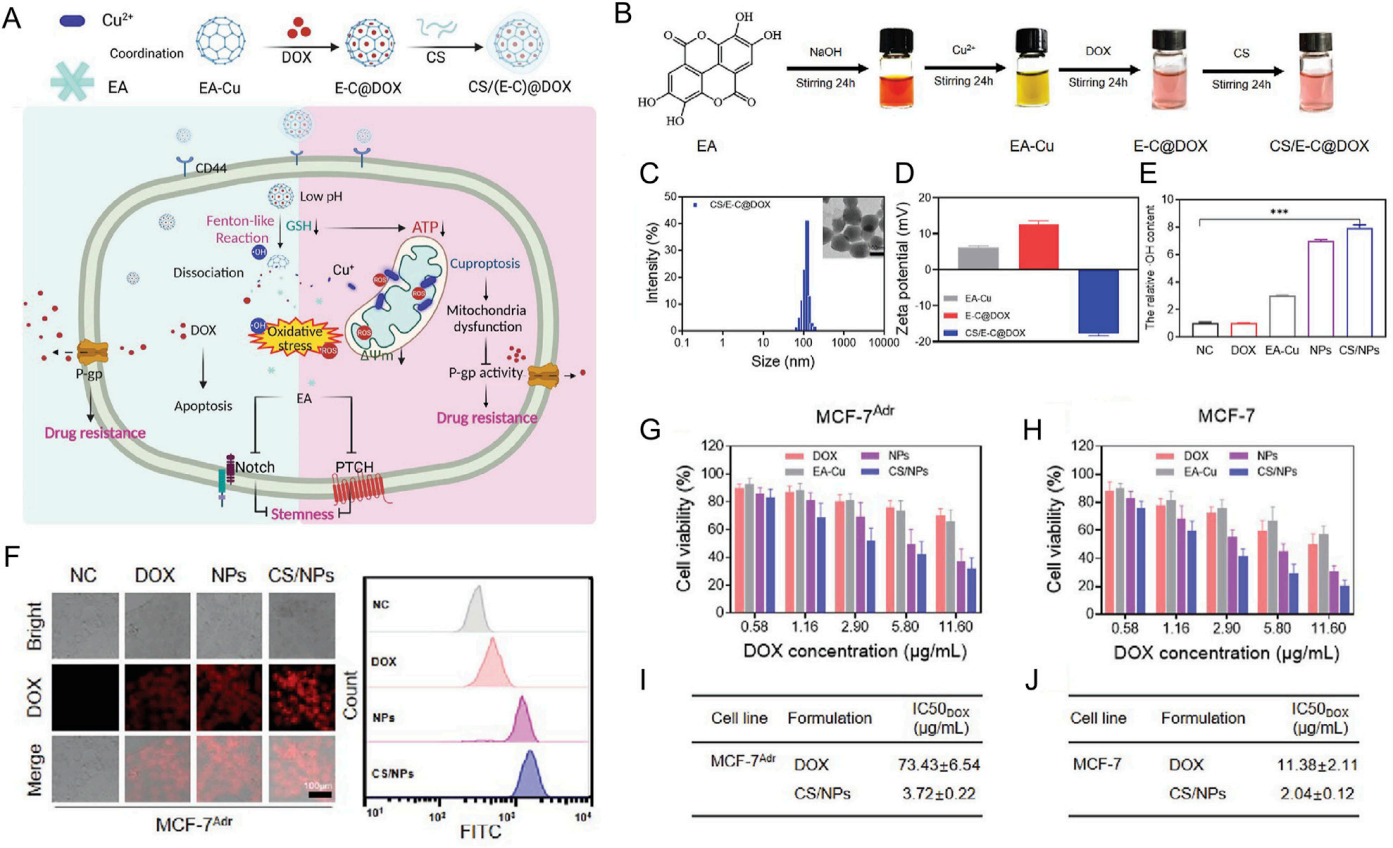
The anti-tumor resistance efficacy of CS/NPs in MCF-7 and doxorubicin-resistant MCF-7^Adr^ cells. **(A)** EA was added to a 0.1 M sodium hydroxide solution, stirred for 24 h, and subsequently mixed with an equimolar copper ion solution. After continuous stirring, the mixture was centrifuged to collect the precipitate. The supernatant was freeze-dried to obtain EA-Cu nanoparticles, which were then combined with DOX at a 1:1 M ratio to form NPs. The resulting NPs were dispersed in a chondroitin sulfate (CS) aqueous solution to generate CS/NPs. **(B)** Schematic representation of CS/NP synthesis. **(C)** TEM images and size distribution analysis of CS/NPs. **(D)** Zeta potential measurements of EA-Cu, NPs, and CS/NPs. **(E)** Detection of hydroxyl radical (·OH) generation using electron spin resonance (ESR) analysis. **(F)** Cellular uptake of DOX, EA-Cu, NPs, and CS/NPs in MCF-7^Adr^ cells following 4 h of incubation. **(G, H)** Cell viability assays for MCF-7^Adr^ and MCF-7 cells treated with the indicated formulations. **(I, J)** Determination of IC_50_ values for DOX across different treatment groups in MCF-7^Adr^ and MCF-7 cells. (Reprinted with permission from Ref. (Lu S. et al., 2024). Copyright 2023 Wiley).

In addition to classical drug efflux transporter-mediated chemoresistance, cancer cells exhibiting stemness characteristics play a critical role in evading the full impact of chemotherapy (Ayob and Ramasamy, 2018). To improve the sensitivity of cancer chemotherapy, researchers adopted a novel approach by coordinating the Hedgehog pathway inhibitor ellagic acid (EA) with Cu^2+^, which was used to develop a nanoscale metal-organic framework (EA-Cu). This framework was then loaded with doxorubicin (DOX) and modified with targeted chondroitin sulfate (CS), resulting in the formation of the CS/E-C@DOX nanoplatform (CS/NPs) (Figure 9A) (Lu S. et al., 2024). EA inhibits stemness maintenance by suppressing the Hedgehog pathway, while Cu^2+^ further diminishes the stemness characteristics of tumor cells through disruption of mitochondrial metabolism, thereby enhancing DOX-mediated chemotherapy. The researchers successfully synthesized CS/NP, as illustrated in Figure 9B. Structural characterization through X-ray diffraction (XRD) revealed that CS/NPs possess an amorphous structure, with an average diameter of 108.2 nm (Figure 9C). As depicted in Figure 9D, the zeta potential of CS/NPs is approximately −19 mV, which helps prevent their aggregation in aqueous solutions. Furthermore, CS/NPs exhibited a high capacity to induce oxidative stress, generating OH as determined by electron spin resonance (ESR), leading to mitochondrial oxidative damage (Figure 9E) (Lu S. et al., 2024). Internalization is crucial for promoting drug accumulation in tumor cells, thereby ensuring effective treatment. To assess the internalization and anti-tumor resistance efficacy of CS/NPs, researchers performed experiments, including immunofluorescence and flow cytometry, using MCF-7 and doxorubicin-resistant MCF-7^Adr^ cells. The data demonstrated that CS/NPs entered tumor cells and achieved maximum intracellular accumulation after 4 h of incubation (Lu S. et al., 2024). Furthermore, CS/NPs exhibited the highest fluorescence intensity in MCF-7, MCF-7Adr, and 4T1 cells compared to other groups, enhancing uptake efficiency in drug-resistant tumor cells (Figure 9F). In evaluating the cytotoxic effect of CS/NPs through cell anti-proliferation experiments, the equivalent dose of CS/NPs exhibited a more pronounced inhibitory effect on cell proliferation in both parental and drug-resistant cancer cells compared to the DOX, EA-Cu, and NPs groups (Figures 9G–J) (Lu S. et al., 2024). Moreover, data from the *in vivo* MCF-7^Adr^ tumor-bearing BALB/c nude mouse model demonstrated that this nanoplatform exhibited multifaceted efficacy, including inhibition of tumor growth, overcoming treatment resistance, alleviating tumor metastasis and recurrence, and exhibiting low toxicity.

In recent years, numerous studies on nanodelivery systems have utilized the mechanism of cuproptosis to address and mitigate drug resistance in malignant tumors. Specifically, in the context of breast cancer treatment, where drug resistance is a significant concern, several nanomaterials—such as Cu/ZIF-8@DSF@GOx/HA (Xia J. et al., 2024), Copper oxide (Cu_4_O_3_) nanoparticles (Abu-Serie and Abdelfattah, 2023), Cu_2_−XTe Nanocubes (Poulose et al., 2016), PWCu nanocapsules (Liao et al., 2024), Cu_9_S_8_ NPs (Guan et al., 2024), and CuPEs@PApt nanoplatforms (Wang S. et al., 2024)—have been investigated (Table 3). These nanomaterials can deliver agents to modulate the concentration of copper ions within cells, thereby inducing cuproptosis. They have demonstrated the ability to reverse or reduce drug resistance to doxorubicin, acquired radiation resistance, and multidrug resistance to some extent. Regarding drug resistance in prostate cancer (Chen et al., 2018), Cu(DDC)_2_ NPs have demonstrated favorable cytotoxic activity against drug-resistant prostate cancer cells, as well as other cancer cells, with an IC_50_ value of approximately 100 nM. Moreover, Cu(DDC)_2_ NPs can induce cell death in paclitaxel-resistant prostate cancer cells (DU145-TXR) via a paraptosis mechanism. In non-small cell lung cancer research on anti-tumor drug resistance (Jiang M. et al., 2022; Jiang Y. et al., 2022), the C4/HSA-C4 NP plays a pivotal role. It targets multiple aspects of the tumor microenvironment (TME), acting on mitochondrial DNA (mtDNA) to kill tumor cells and induce apoptosis. Furthermore, it can polarize M2 macrophages into M1 macrophages and suppress angiogenesis, ultimately overcoming cisplatin resistance.

**TABLE 3:** Overview of cuproptosis related-nanoplatform overcome tumor resistance.

NanoPlatform	Nanomaterials	Cancer type	Anti-tumor resistance mechanism	Ref
DSF@CuO gel	Sodium alginate	HCC	DSF@CuO gel induces mitochondrial dysfunction and triggers cuproptosis in lenvatinib-resistant hepatocellular carcinoma cells through the downregulation of DLAT, LIAS, and CDKN2A, while enhancing the expression of FDX1	Li et al. (2024c)
CS/E-C@DOX nanoplatform (CS/NPs)	Chondroitin sulfate (CS)	BC	CS/NPs exhibit potent anti-tumor activity and effectively reverse doxorubicin resistance	Lu et al. (2024b)
Cu/ZIF-8@DSF@GOx/HA	Zeolitic imidazolate framework	BC and HCC	*In situ*, chelation of Cu^2+^ with DSF generates cytotoxic CuETs, and the concomitant depletion of ATP inhibits P-glycoprotein function, thereby reducing CuET efflux and overcoming multidrug resistance-mediated chemotherapy	Xia et al. (2024b)
Copper oxide (Cu_4_O_3_) nanoparticles, CD NPs	Diethyldithiocarbamate	MBC	CD NPs demonstrate a greater potential to induce apoptosis, inhibit hypoxia-inducible factor genes, and eradicate CD44^+^ cancer stem cells by downregulating key genes associated with stemness, chemoresistance, and metastasis, while also reducing hepatic tumor markers, such as α-fetoprotein	Abu-Serie and Abdelfattah (2023)
Cu(DDC)_2_ NPs	PLA-PEG	PC	Cu(DDC)_2_ nanoparticles (NPs) demonstrate potent anti-cancer activity, inducing paraptosis in drug-resistant prostate cancer cells (DU145-TXR) with an IC_50_ of approximately 100 nM	Chen et al. (2018)
Cu_2−X_Te Nanocubes	Copper telluride	BC	Multifunctional Cu_2-X_Te nanocubes, exhibiting strong near-infrared absorbance, serve as effective chemo-photothermal-photodynamic agents for cancer therapy and are capable of overcoming resistance in hypermethylated cancer cells, while also enabling photoacoustic and X-ray contrast imaging	Poulose et al. (2016)
HSA-C4 NPs	HSA	NSCLC	HSA-C4 NPs effectively inhibit A549cisR tumor growth and metastasis by targeting multiple aspects of TME, including inducing apoptosis through mtDNA damage, reprogramming M2 macrophages to the M1 phenotype, and inhibiting angiogenesis, thereby overcoming cisplatin resistance	Jiang et al. (2022b), Jiang et al. (2022c)
PWCu nanocapsules	CuO_6_ octahedra encapsulated	BC	PWCu nanocapsule releases copper ions in a controlled manner upon ionizing radiation, triggering cuproptosis that overcomes acquired radiation resistance at clinically relevant doses while inducing a robust abscopal effect and achieving a 40% cure rate in both radioresistant and re-irradiation tumor models	Liao et al. (2024)
Cu_9_S_8_ NPs, CAPSH	Cu_9_S_8_	BC	Cu_9_S_8_-based nanoplatform (CAPSH) precisely targets tumor tissues, enhances cuproptosis through ATP7A interference, and integrates thermodynamic therapy with immune modulation	Guan et al. (2024)
CuPEs@PApt	Polyvalent aptamer	BC	CuPEs@PApt efficiently targets tumor cells, inducing cuproptosis and immunogenic cell death while ensuring accurate, safe delivery of cuproptosis-inducing agents to enhance tumor cell destruction	Wang et al. (2024g)

Nanodelivery holds considerable promise for cuproptosis-based cancer treatment. However, several challenges impede its clinical application. Key concerns include biocompatibility and safety, as the long-term effects of many nanomaterials remain unclear, necessitating further investigation to ensure minimal toxicity in humans. Moreover, individual variability in tumor microenvironments can influence the delivery efficiency and therapeutic efficacy of nanocarriers, highlighting the importance of personalized treatment strategies. Furthermore, the development of high-quality, controllable manufacturing processes is essential for large-scale production and clinical use of these nanocarriers, as complex manufacturing techniques may limit their broader application. By encapsulating copper or related drugs within nanocarriers, nanodelivery technology enhances targeting, stability, and minimizes side effects, positioning it as a promising platform for future cancer therapies, particularly for difficult-to-treat tumors. Nonetheless, further clinical research is crucial to overcoming these challenges and facilitating the transition of this technology from the laboratory to clinical practice.

## 4 Conclusion

Copper serves as a crucial cofactor for numerous enzymes involved in redox reactions. For example, cytochrome c oxidase, located in the inner mitochondrial membrane, is a critical component of the electron transport chain, facilitating the final oxidation-reduction step that generates water molecules. Copper-zinc superoxide dismutase (Cu/Zn-SOD) is essential for converting superoxide radicals into oxygen and hydrogen peroxide, functioning as an antioxidant that protects cells from oxidative damage (Feng et al., 2016; Kim et al., 2011). Moreover, copper is integral to iron metabolism, particularly in regulating iron absorption, transport, and storage (Doguer et al., 2018; Tang et al., 2024). Ceruloplasmin, a copper-containing enzyme synthesized in the liver, oxidizes Fe^2+^ to Fe^3+^, facilitating transferrin-mediated iron transport throughout the body. Furthermore, copper is crucial for the proper functioning of the nervous system, influencing neurotransmitter synthesis and release (Schmidt et al., 2018). Copper-dependent enzymes, such as dopamine β-hydroxylase, play a role in converting dopamine into norepinephrine. In the immune system, copper is critical for the proliferation and functionality of immune cells; deficiencies can result in leukopenia and impaired immune responses (Muñoz et al., 2007; Lu J. et al., 2024). Moreover, copper functions as a cofactor for lysyl oxidase, which catalyzes the cross-linking of collagen and elastin, essential for maintaining the structural integrity and elasticity of connective tissues (Kothapalli and Ramamurthi, 2009; Ogen-Shtern et al., 2020). Copper is also essential to the antioxidant enzyme system, where it helps neutralize free radicals and reduce oxidative stress, thereby protecting cellular integrity. However, maintaining copper homeostasis is crucial, as abnormal copper metabolism can result in various diseases. For example, Wilson disease is a rare genetic disorder characterized by disrupted copper metabolism in the liver and other tissues, leading to elevated copper levels and subsequent accumulation in organs, causing severe pathological changes. Moreover, abnormal copper metabolism is associated with conditions such as Menkes disease.

Cuproptosis, an emerging form of cell death, has gained significant attention in recent years, particularly in cancer research (Han et al., 2024; Teschke and Eickhoff, 2024; Kahlson and Dixon, 2022). Copper is an essential trace element that plays a crucial role in numerous biological processes, including mitochondrial respiration, antioxidant defense, and enzyme catalysis. However, dysregulation of copper homeostasis can result in pathological conditions such as neurodegenerative diseases and cancer. The discovery that excessive accumulation of copper can induce cell death through a distinct pathway has opened new avenues for therapeutic intervention. Unlike traditional apoptosis or necrosis, cuproptosis involves distinct mechanisms, including the direct binding of copper to fatty acyl components of the TCA cycle, resulting in protein aggregation, mitochondrial dysfunction, and subsequent cell death (Kahlson and Dixon, 2022). This understanding has stimulated the development of copper-based therapeutic strategies, including copper ionophores, chelators, and complexes, aimed at exploiting the vulnerability of cancer cells to copper-induced cytotoxicity. Furthermore, the complex interactions between copper metabolism and various signaling pathways offer potential for combination therapies, enhancing the efficacy of existing cancer treatments. As research advances, the focus is shifting towards optimizing the selectivity and delivery of copper-based formulations to maximize therapeutic efficacy while minimizing off-target effects. Promising preclinical results emphasize the need for further investigation to translate these findings into clinical applications, paving the way for innovative and effective cancer therapies. Several preclinical studies and early-phase clinical trials of cuproptosis inducers are currently underway, primarily focusing on evaluating the safety, efficacy, and synergistic effects of cuproptosis inducers in combination with other treatment modalities.

This review provides a comprehensive overview of the latest advancements in cuproptosis research and its significance in tumor resistance therapy. This review systematically summarizes the research progress regarding copper ionophores, copper chelators related to cuproptosis, and nanodelivery systems in the field of anti-tumor drug resistance. Current *in vitro* and *in vivo* studies have clearly demonstrated that copper ionophores (ES, DSF, CuATSM, and WN197) can enhance anti-tumor activity by regulating the intracellular copper ion concentration and inhibiting the functions of DNA damage repair proteins, thereby providing a novel approach to overcome tumor drug resistance. On the other hand, copper chelators (TM, D-penicillamine, TETA, and CQ) are capable of regulating the abnormal overload state of copper ions, consequently restoring the sensitivity of tumor cells to chemotherapy drugs. Meanwhile, nanodelivery systems have exhibited remarkable advantages in improving the tumor targeting ability of these substances and optimizing their pharmacokinetic properties. Moreover, various combination application strategies have also enhanced the anti-tumor drug resistance effect to a certain extent, laying a solid foundation for further in-depth exploration. A deeper understanding of cuproptosis and its therapeutic potential may lead to more effective and safer cancer treatments.

## 5 Future directions

Cuproptosis, an emerging form of cell death, has demonstrated promising potential in evaluating anti-tumor drug resistance, as evidenced by numerous studies. However, several challenges remain in understanding the mechanism of cuproptosis. As an emerging tumor treatment modality, the clinical application of copper death may be limited by delivery efficiency, drug resistance mechanisms, and the absence of biomarkers. For example, the mechanism of cuproptosis involves a complex array of molecular regulatory networks and intricate copper ion metabolism processes within cells. Moreover, developing copper ionophores and chelators that specifically target tumor cells while maintaining robust safety profiles remains a significant challenge. Furthermore, the goal of nanodelivery systems is to facilitate the precise targeting of drugs to tumor sites, enabling on-demand release. In addition, constructing multifunctional nanodelivery platforms capable of combining multiple anti-tumor drugs to exert synergistic effects remains a challenge. Despite these challenges, copper ionophores, copper chelators associated with cuproptosis, and nanodelivery systems offer promising prospects in the field of anti-tumor drug resistance. Through interdisciplinary collaboration, the integration of multiple technologies, and the close combination of basic research with clinical applications, substantial breakthroughs are expected in overcoming tumor drug resistance, ultimately offering more effective treatment options for cancer patients. Systematic approaches such as CRISPR screening will be necessary to analyze drug resistance driver genes, and strategies can be optimized through the integration of nanotechnology, metabolic intervention, and immunotherapy. For instance, by utilizing a whole-genome CRISPR knockout library combined with copper death inducers, key genes regulating copper sensitivity (such as copper transporter genes and mitochondrial metabolism-related genes) can be identified. Additionally, integrating the IC_50_ data of cell lines to copper death inducers with gene editing results can verify the impact of candidate genes on drug sensitivity (Cheng et al., 2024; Liu and Wang, 2024). Focusing on multi-target combination therapies, personalized precision treatments, innovative carrier development, and in-depth mechanistic studies can significantly enhance the effectiveness of copper-based anti-tumor therapies, providing safer, more effective options for patients. Moreover, investigating the potential synergistic anti-tumor effects of inhibiting mitochondrial respiration and glycolysis warrants further exploration.

## References

[B1] Abo El-Maali N. Badr G. Sayed D. Adam R. Abd El Wahab G. (2019). Enhanced susceptibility to apoptosis and growth arrest of human breast carcinoma cells treated with silica nanoparticles loaded with monohydroxy flavone compounds. Biochem. Cell Biol. 97 (5), 513–525. 10.1139/bcb-2018-0133 30640511

[B2] Abu-Serie M. M. Abdelfattah E. Z. A. (2023). A comparative study of smart nanoformulations of diethyldithiocarbamate with Cu_4_O_3_ nanoparticles or zinc oxide nanoparticles for efficient eradication of metastatic breast cancer. Sci. Rep. 13 (1), 3529. 10.1038/s41598-023-30553-8 36864097 PMC9981580

[B3] Aida T. Takebayashi Y. Shimizu T. Okamura C. Higasimoto M. Kanzaki A. (2005). Expression of copper-transporting P-type adenosine triphosphatase (ATP7B) as a prognostic factor in human endometrial carcinoma. Gynecol. Oncol. 97 (1), 41–45. 10.1016/j.ygyno.2004.12.042 15790435

[B4] Andres S. A. Bajaj K. Vishnosky N. S. Peterson M. A. Mashuta M. S. Buchanan R. M. (2020). Synthesis, characterization, and biological activity of Hybrid thiosemicarbazone-Alkylthiocarbamate metal complexes. Inorg. Chem. 59, 4924–4935. 10.1021/acs.inorgchem.0c00182 32159342

[B5] Antos A. Członkowska A. Bembenek J. Kurkowska-Jastrzębska I. Litwin T. (2023). D-Penicillamine-Induced Myasthenia Gravis-A Probable Complication of Wilson's disease treatment-A case report and systematic review of the Literature. Life (Basel) 13 (8), 1715. 10.3390/life13081715 37629572 PMC10455431

[B6] Arnesano F. Natile G. (2021). Interference between copper transport systems and platinum drugs. Semin. Cancer Biol. 76, 173–188. 10.1016/j.semcancer.2021.05.023 34058339

[B7] Ayob A. Z. Ramasamy T. S. (2018). Cancer stem cells as key drivers of tumour progression. J. Biomed. Sci. 25 (1), 20. 10.1186/s12929-018-0426-4 29506506 PMC5838954

[B8] Barakat D. J. Mendonca J. Barberi T. Zhang J. Kachhap S. K. Paz-Priel I. (2016). C/EBPβ regulates sensitivity to bortezomib in prostate cancer cells by inducing REDD1 and autophagosome-lysosome fusion. Cancer Lett. 375 (1), 152–161. 10.1016/j.canlet.2016.03.005 26968249 PMC4818955

[B9] Bareggi S. R. Cornelli U. (2012). Clioquinol: review of its mechanisms of action and clinical uses in neurodegenerative disorders. CNS Neurosci. Ther. 18, 41–46. 10.1111/j.1755-5949.2010.00231.x 21199452 PMC6493473

[B10] Batzios S. Tal G. DiStasio A. T. Peng Y. Charalambous C. Nicolaides P. (2022). Newly identified disorder of copper metabolism caused by variants in CTR1, a high-affinity copper transporter. Hum. Mol. Genet. 31, 4121–4130. 10.1093/hmg/ddac156 35913762 PMC9759326

[B11] Baum L. Ng A. (2004). Curcumin interaction with copper and iron suggests one possible mechanism of action in Alzheimer's disease animal models. J. Alzheimers Dis. 6, 367–449. 10.3233/jad-2004-6403 15345806

[B12] Bian C. Zheng Z. Su J. Chang S. Yu H. Bao J. (2023). Copper homeostasis and cuproptosis in tumor pathogenesis and therapeutic strategies. Front. Pharmacol. 14, 1271613. 10.3389/fphar.2023.1271613 37767404 PMC10520736

[B13] Bing Z. Han J. Zheng Z. Liang N. (2021). FOXO3-induced oncogenic lncRNA CASC9 enhances gefitinib resistance of non-small-cell lung cancer through feedback loop. Life Sci. 287, 120012. 10.1016/j.lfs.2021.120012 34619168

[B14] Bista R. Lee D. W. Pepper O. B. Azorsa D. O. Arceci R. J. Aleem E. (2017). Disulfiram overcomes bortezomib and cytarabine resistance in Down-syndrome-associated acute myeloid leukemia cells. J. Exp. Clin. Cancer Res. CR 36 (1), 22. 10.1186/s13046-017-0493-5 28143565 PMC5286849

[B15] Boufraqech M. Patel D. Nilubol N. Powers A. King T. Shell J. (2019). Lysyl oxidase is a key player in BRAF/MAPK pathway-driven thyroid cancer aggressiveness. Thyroid 29 (1), 79–92. 10.1089/thy.2018.0424 30398411 PMC6352555

[B16] Brady D. C. Crowe M. S. Greenberg D. N. Counter C. M. (2017). Copper chelation inhibits BRAFV600E-driven Melanomagenesis and Counters resistance to BRAFV600E and MEK1/2 inhibitors. Cancer Res. 77 (22), 6240–6252. 10.1158/0008-5472.CAN-16-1190 28986383 PMC5690876

[B17] Branch A. H. Stoudenmire J. L. Seib K. L. Cornelissen C. N. (2022). Acclimation to nutritional immunity and metal Intoxication requires zinc, Manganese, and copper homeostasis in the pathogenic Neisseriae. Front. Cell. Infect. Microbiol. 12, 909888. 10.3389/fcimb.2022.909888 35846739 PMC9280163

[B18] Brischigliaro M. Zeviani M. (2021). Cytochrome c oxidase deficiency. Biochim. Biophys. Acta Bioenerg. 1862 (1), 148335. 10.1016/j.bbabio.2020.148335 33171185

[B19] Cai H. Wu J. S. Muzik O. Hsieh J. T. Lee R. J. Peng F. (2014). Reduced 64Cu uptake and tumor growth inhibition by knockdown of human copper transporter 1 in xenograft mouse model of prostate cancer. J. Nucl. Med. 55 (4), 622–628. 10.2967/jnumed.113.126979 24639459 PMC4600613

[B20] Cao H. Z. Yang W. T. Zheng P. S. (2022). Cytotoxic effect of disulfiram/copper on human cervical cancer cell lines and LGR5-positive cancer stem-like cells. BMC Cancer 22 (1), 521. 10.1186/s12885-022-09574-5 35534815 PMC9082913

[B21] Cater M. A. Pearson H. B. Wolyniec K. Klaver P. Bilandzic M. Paterson B. M. (2013). Increasing intracellular bioavailable copper selectively targets prostate cancer cells. ACS Chem. Biol. 8, 1621–1631. 10.1021/cb400198p 23656859

[B22] Chan N. Willis A. Kornhauser N. Ward M. M. Lee S. B. Nackos E. (2017). Influencing the tumor microenvironment: a phase II study of copper depletion using tetrathiomolybdate in patients with breast cancer at high risk for recurrence and in preclinical models of lung Metastases. Clin. Cancer Res. 23, 666–676. 10.1158/1078-0432.CCR-16-1326 27769988

[B23] Chan P. Chen Y. C. Lin L. J. Cheng T. H. Anzai K. Chen Y. H. (2012). Tanshinone IIA Attenuates H_2_O_2_ -induced injury in human umbilical vein endothelial cells. Am. J. Chin. Med. 40 (6), 1307–1319. 10.1142/S0192415X12500966 23227799

[B24] Chen J. Jiang Y. Shi H. Peng Y. Fan X. Li C. (2020). The molecular mechanisms of copper metabolism and its roles in human diseases. Pflugers. Arch. 472, 1415–1429. 10.1007/s00424-020-02412-2 32506322

[B25] Chen L. Min J. Wang F. (2022). Copper homeostasis and cuproptosis in health and disease. Signal. Transduct. Target Ther. 7, 378. 10.1038/s41392-022-01229-y 36414625 PMC9681860

[B26] Chen S. J. Kuo C. C. Pan H. Y. Tsou T. C. Yeh S. C. Chang J. Y. (2015). Mechanistic basis of a combination D-penicillamine and platinum drugs synergistically inhibits tumor growth in oxaliplatin-resistant human cervical cancer cells *in vitro* and *in vivo* . Biochem. Pharmacol. 95, 28–37. 10.1016/j.bcp.2015.03.006 25801007

[B27] Chen W. Yang W. Chen P. Huang Y. Li F. (2018). Disulfiram copper nanoparticles prepared with a stabilized metal ion Ligand complex method for treating drug-resistant prostate cancers. ACS Appl. Mater. Interfaces 10 (48), 41118–41128. 10.1021/acsami.8b14940 30444340

[B28] Chen X. Li K. Xiao Y. Wu W. Lin H. Qing X. (2024a). SP1/CTR1-mediated oxidative stress-induced cuproptosis in intervertebral disc degeneration. BioFactors 50 (5), 1009–1023. 10.1002/biof.2052 38599595

[B29] Chen X. Xiang W. Li L. Xu K. (2024b). Copper chaperone Atox1 protected the Cochlea from cisplatin by regulating the copper transport family and cell cycle. Int. J. Toxicol. 43 (2), 134–145. 10.1177/10915818231206665 37859596

[B30] Cheng J. Luo Z. Chen G. H. Wei C. C. Zhuo M. Q. (2017). Identification of eight copper (Cu) uptake related genes from yellow catfish Pelteobagrus fulvidraco, and their tissue expression and transcriptional responses to dietborne Cu exposure. J. Trace Elem. Med. Biol. 44, 256–265. 10.1016/j.jtemb.2017.09.004 28965584

[B31] Cheng X. Yang F. Li Y. Cao Y. Zhang M. Ji J. (2024). The crosstalk role of CDKN2A between tumor progression and cuproptosis resistance in colorectal cancer. Aging 16 (12), 10512–10538. 10.18632/aging.205945 38888512 PMC11236303

[B32] Chisholm C. L. Wang H. Wong A. H. Vazquez-Ortiz G. Chen W. Xu X. (2016). Ammonium tetrathiomolybdate treatment targets the copper transporter ATP7A and enhances sensitivity of breast cancer to cisplatin. Oncotarget 7 (51), 84439–84452. 10.18632/oncotarget.12992 27806319 PMC5341295

[B33] Conilh L. Fournet G. Fourmaux E. Murcia A. Matera E. L. Joseph B. (2021). Exatecan Antibody drug Conjugates based on a Hydrophilic Polysarcosine drug-Linker platform. Pharm. (Basel) 14 (3), 247. 10.3390/ph14030247 PMC8000490 33803327

[B34] Das A. Ash D. Fouda A. Y. Sudhahar V. Kim Y. M. Hou Y. (2022). Cysteine oxidation of copper transporter CTR1 drives VEGFR2 signalling and angiogenesis. Nat. Cell Biol. 24 (1), 35–50. 10.1038/s41556-021-00822-7 35027734 PMC8851982

[B35] Da Silva D. A. de Luca A. Squitti R. Rongioletti M. Rossi L. Machado C. M. L. (2022). Copper in tumors and the use of copper-based compounds in cancer treatment. J. Inorg. Biochem. 226, 111634. 10.1016/j.jinorgbio.2021.111634 34740035

[B36] Ding X. Xie H. Kang Y. J. (2011). The significance of copper chelators in clinical and experimental application. J. Nutr. Biochem. 22, 301–310. 10.1016/j.jnutbio.2010.06.010 21109416

[B37] Doguer C. Ha J. H. Collins J. F. (2018). Intersection of iron and copper metabolism in the mammalian intestine and liver. Compr. Physiol. 8 (4), 1433–1461. 10.1002/cphy.c170045 30215866 PMC6460475

[B38] Dolgova N. V. Olson D. Lutsenko S. Dmitriev O. Y. (2009). The soluble metal-binding domain of the copper transporter ATP7B binds and detoxifies cisplatin. Biochem. J. 419 (1), 51–56. 10.1042/BJ20081359 19173677 PMC2825889

[B39] Ejaz H. W. Wang W. Lang M. (2020). Copper toxicity links to pathogenesis of Alzheimer's disease and therapeutics approaches. Int. J. Mol. Sci. 21, 7660. 10.3390/ijms21207660 33081348 PMC7589751

[B40] Eun J. W. Yoon J. H. Ahn H. R. Kim S. Kim Y. B. Lim S. B. (2023). Cancer-associated fibroblast-derived secreted phosphoprotein 1 contributes to resistance of hepatocellular carcinoma to sorafenib and lenvatinib. Cancer Commun. 43 (4), 455–479. 10.1002/cac2.12414 PMC10091107 36919193

[B41] Falls-Hubert K. C. Butler A. L. Gui K. Anderson M. Li M. Stolwijk J. M. (2020). Disulfiram causes selective hypoxic cancer cell toxicity and radio-chemo-sensitization via redox cycling of copper. Free Radic. Biol. Med. 150, 1–11. 10.1016/j.freeradbiomed.2020.01.186 32032663 PMC7299833

[B42] Fang T. Chen W. Sheng Y. Yuan S. Tang Q. Li G. (2019). Tetrathiomolybdate induces dimerization of the metal-binding domain of ATPase and inhibits platination of the protein. Nat. Commun. 10 (1), 186. 10.1038/s41467-018-08102-z 30643139 PMC6331642

[B43] Feng X. Chen F. Liu W. Thu M. K. Zhang Z. Chen Y. (2016). Molecular characterization of MaCCS, a novel copper chaperone gene involved in Abiotic and hormonal stress responses in Musa acuminata cv. Tianbaojiao. Int. J. Mol. Sci. 17, 441. 10.3390/ijms17040441 27023517 PMC4848897

[B44] Feng Y. Huang Z. Song L. Li N. Li X. Shi H. (2024b). PDE3B regulates KRT6B and increases the sensitivity of bladder cancer cells to copper ionophores. Naunyn Schmiedeb. Arch. Pharmacol. 397 (7), 4911–4925. 10.1007/s00210-023-02928-1 38165426

[B45] Feng Y. Yang Z. Wang J. Zhao H. (2024a). Cuproptosis: unveiling a new frontier in cancer biology and therapeutics. Cell Commun. Signal. 22 (1), 249. 10.1186/s12964-024-01625-7 38693584 PMC11064406

[B46] Fujimoto M. Kito H. Kajikuri J. Ohya S. (2018). Transcriptional repression of human epidermal growth factor receptor 2 by ClC-3 Cl^-^/H^+^ transporter inhibition in human breast cancer cells. Cancer Sci. 109 (9), 2781–2791. 10.1111/cas.13715 29949674 PMC6125433

[B47] Fung K. L. Tepede A. K. Pluchino K. M. Pouliot L. M. Pixley J. N. Hall M. D. (2014). Uptake of compounds that selectively kill multidrug-resistant cells: the copper transporter SLC31A1 (CTR1) increases cellular accumulation of the thiosemicarbazone NSC73306. Mol. Pharm. 11 (8), 2692–2702. 10.1021/mp500114e 24800945 PMC4137994

[B48] Gaál A. Orgován G. Mihucz V. G. Pape I. Ingerle D. Streli C. (2018). Metal transport capabilities of anticancer copper chelators. J. Trace. Elem. Med. Biol. 47, 79–88. 10.1016/j.jtemb.2018.01.011 29544811

[B49] Gale J. Aizenman E. (2024). The physiological and pathophysiological roles of copper in the nervous system. Eur. J. Neurosci. 60, 3505–3543. 10.1111/ejn.16370 38747014 PMC11491124

[B50] Gao F. Yuan Y. Ding Y. Li P. Y. Chang Y. He X. X. (2023c). DLAT as a cuproptosis promoter and a molecular target of elesclomol in hepatocellular carcinoma. Curr. Med. Sci. 43, 526–538. 10.1007/s11596-023-2755-0 37286711

[B51] Gao J. Wang Q. Tang Y. D. Zhai J. Hu W. Zheng C. (2023a). When ferroptosis meets pathogenic infections. Trends Microbiol. 31 (5), 468–479. 10.1016/j.tim.2022.11.006 36496309

[B52] Gao J. Wu X. Huang S. Zhao Z. He W. Song M. (2023b). Novel insights into anticancer mechanisms of elesclomol: more than a prooxidant drug. Redox Biol. 67, 102891. 10.1016/j.redox.2023.102891 37734229 PMC10518591

[B53] Gao X. Zhao H. Liu J. Wang M. Dai Z. Hao W. (2024). Enzalutamide sensitizes Castration-resistant prostate cancer to copper-mediated cell death. Adv. Sci. 11, e2401396. 10.1002/advs.202401396 PMC11321675 38859590

[B54] Garza N. M. Griffin A. T. Zulkifli M. Qiu C. Kaplan C. D. Gohil V. M. (2021). A genome-wide copper-sensitized screen identifies novel regulators of mitochondrial cytochrome c oxidase activity. J. Biol. Chem. 296, 100485. 10.1016/j.jbc.2021.100485 33662401 PMC8027276

[B55] Garza N. M. Swaminathan A. B. Maremanda K. P. Zulkifli M. Gohil V. M. (2023). Mitochondrial copper in human genetic disorders. Trends Endocrinol. Metab. 34 (1), 21–33. 10.1016/j.tem.2022.11.001 36435678 PMC9780195

[B56] Gateau C. Delangle P. (2014). Design of intrahepatocyte copper(I) chelators as drug candidates for Wilson's disease. Ann. N. Y. Acad. Sci. 1315, 30–36. 10.1111/nyas.12379 24611802

[B57] Geng R. Ke N. Wang Z. Mou Y. Xiang B. Zhang Z. (2023). Copper deprivation enhances the chemosensitivity of pancreatic cancer to rapamycin by mTORC1/2 inhibition. Chem. Biol. Interact. 382, 110546. 10.1016/j.cbi.2023.110546 37290678

[B58] Gou R. Li X. Dong H. Hu Y. Liu O. Liu J. (2022). RAD21 confers poor prognosis and Affects ovarian cancer sensitivity to poly(ADP-Ribose)Polymerase inhibitors through DNA damage repair. Front. Oncol. 12, 936550. 10.3389/fonc.2022.936550 35860572 PMC9289200

[B59] Guan M. Cheng K. Xie X. T. Li Y. Ma M. W. Zhang B. (2024). Regulating copper homeostasis of tumor cells to promote cuproptosis for enhancing breast cancer immunotherapy. Nat. Commun. 15 (1), 10060. 10.1038/s41467-024-54469-7 39567558 PMC11579316

[B60] Guo B. Yang F. Zhang L. Zhao Q. Wang W. Yin L. (2023). Cuproptosis induced by ROS responsive nanoparticles with elesclomol and copper combined with αPD-L1 for enhanced cancer immunotherapy. Adv. Mater 35 (22), e2212267. 10.1002/adma.202212267 36916030

[B61] Guo J. Cheng J. Zheng N. Zhang X. Dai X. Zhang L. (2021). Copper promotes tumorigenesis by activating the PDK1-AKT oncogenic pathway in a copper transporter 1 dependent manner. Adv. Sci. 8, e2004303. 10.1002/advs.202004303 PMC8456201 34278744

[B62] Gupta P. Gao H. L. Ashar Y. V. Karadkhelkar N. M. Yoganathan S. Chen Z. S. (2019). Ciprofloxacin enhances the chemosensitivity of cancer cells to ABCB1 substrates. Int. J. Mol. Sci. 20 (2), 268. 10.3390/ijms20020268 30641875 PMC6358874

[B63] Gupte A. Mumper R. J. (2007). Copper chelation by D-penicillamine generates reactive oxygen species that are cytotoxic to human leukemia and breast cancer cells. Free. Radic. Biol. Med. 43, 1271–1278. 10.1016/j.freeradbiomed.2007.07.003 17893040

[B64] Han I. W. Jang J. Y. Kwon W. Park T. Kim Y. Lee K. B. (2017). Ceruloplasmin as a prognostic marker in patients with bile duct cancer. Oncotarget 8 (17), 29028–29037. 10.18632/oncotarget.15995 28423673 PMC5438709

[B65] Han J. Luo J. Wang C. Kapilevich L. Zhang X. A. (2024). Roles and mechanisms of copper homeostasis and cuproptosis in osteoarticular diseases. Biomed. Pharmacother. 174, 116570. 10.1016/j.biopha.2024.116570 38599063

[B66] Hasim M. S. Nessim C. Villeneuve P. J. Vanderhyden B. C. Dimitroulakos J. (2018). Activating transcription factor 3 as a novel regulator of chemotherapy response in breast cancer. Transl. Oncol. 11 (4), 988–998. 10.1016/j.tranon.2018.06.001 29940414 PMC6039300

[B67] Hatori Y. Inouye S. Akagi R. (2017). Thiol-based copper handling by the copper chaperone Atox1. IUBMB life 69 (4), 246–254. 10.1002/iub.1620 28294521

[B68] Heuberger D. M. Harankhedkar S. Morgan T. Wolint P. Calcagni M. Lai B. (2019). High-affinity Cu(I) chelator PSP-2 as potential anti-angiogenic agent. Sci. Rep. 9 (1), 14055. 10.1038/s41598-019-50494-5 31575910 PMC6773859

[B69] Holland J. P. Giansiracusa J. H. Bell S. G. Wong L. L. Dilworth J. R. (2009). *In vitro* kinetic studies on the mechanism of oxygen-dependent cellular uptake of copper radiopharmaceuticals. Phys. Med. Biol. 54, 2103–2119. 10.1088/0031-9155/54/7/017 19287086

[B70] Horn S. Leonardelli S. Sucker A. Schadendorf D. Griewank K. G. Paschen A. (2018). Tumor CDKN2A-associated JAK2 loss and susceptibility to immunotherapy resistance. J. Natl. Cancer Inst. 110 (6), 677–681. 10.1093/jnci/djx271 29917141

[B71] Huang D. Chen L. Ji Q. Xiang Y. Zhou Q. Chen K. (2024b). Lead aggravates Alzheimer's disease pathology via mitochondrial copper accumulation regulated by COX17. Redox Biol. 69, 102990. 10.1016/j.redox.2023.102990 38091880 PMC10716782

[B72] Huang J. Chaudhary R. Cohen A. L. Fink K. Goldlust S. Boockvar J. (2019a). A multicenter phase II study of temozolomide plus disulfiram and copper for recurrent temozolomide-resistant glioblastoma. J. Neurooncol. 142 (3), 537–544. 10.1007/s11060-019-03125-y 30771200

[B73] Huang M. Zhang Y. Liu X. (2024a). The mechanism of cuproptosis in Parkinson's disease. Ageing. Res. Rev. 95, 102214. 10.1016/j.arr.2024.102214 38311254

[B74] Huang P. Wu G. Huang M. Deng Y. Chen X. Ye G. (2025). Copper-coordinated nanomedicine for the concurrent treatment of lung cancer through the induction of cuproptosis and apoptosis. Eur. J. Pharm. Sci. 204, 106942. 10.1016/j.ejps.2024.106942 39437977

[B75] Huang T. Zhang Y. Wu Y. Han X. Li L. Guo Z. (2024c). CEBPB dampens the cuproptosis sensitivity of colorectal cancer cells by facilitating the PI3K/AKT/mTOR signaling pathway. Saudi. J. Gastroenterol. 30 (6), 381–388. 10.4103/sjg.sjg_169_24 39246119 PMC11630481

[B76] Huang X. D. (2023). A Concise review on oxidative stress-mediated ferroptosis and cuproptosis in Alzheimer's disease. Cells 12, 1369. 10.3390/cells12101369 37408203 PMC10216514

[B77] Huang Y. F. Kuo M. T. Liu Y. S. Cheng Y. M. Wu P. Y. Chou C. Y. (2019b). A dose Escalation study of trientine plus carboplatin and pegylated liposomal doxorubicin in Women with a First Relapse of epithelial ovarian, tubal, and peritoneal cancer within 12 Months after platinum-based chemotherapy. Front. Oncol. 9, 437. 10.3389/fonc.2019.00437 31179244 PMC6544081

[B78] Huo Q. Zhu J. Niu Y. Shi H. Gong Y. Li Y. (2017). pH-triggered surface charge-switchable polymer micelles for the co-delivery of paclitaxel/disulfiram and overcoming multidrug resistance in cancer. Int. J. Nanomedicine 12, 8631–8647. 10.2147/IJN.S144452 29270012 PMC5720040

[B79] Huo S. Wang Q. Shi W. Peng L. Jiang Y. Zhu M. (2023). ATF3/SPI1/SLC31A1 signaling promotes cuproptosis induced by advanced Glycosylation end products in Diabetic Myocardial injury. Int. J. Mol. Sci. 24, 1667. 10.3390/ijms24021667 36675183 PMC9862315

[B80] Ishida S. Andreux P. Poitry-Yamate C. Auwerx J. Hanahan D. (2013). Bioavailable copper modulates oxidative phosphorylation and growth of tumors. Proc. Natl. Acad. Sci. U. S. A. 110 (48), 19507–19512. 10.1073/pnas.1318431110 24218578 PMC3845132

[B81] Ishida S. McCormick F. Smith-McCune K. Hanahan D. (2010). Enhancing tumor-specific uptake of the anticancer drug cisplatin with a copper chelator. Cancer cell 17 (6), 574–583. 10.1016/j.ccr.2010.04.011 20541702 PMC2902369

[B82] Ishigami I. Sierra R. G. Su Z. Peck A. Wang C. Poitevin F. (2023). Structural insights into functional properties of the oxidized form of cytochrome c oxidase. Nat. Commun. 14, 5752. 10.1038/s41467-023-41533-x 37717031 PMC10505203

[B83] Itoh S. Kim H. W. Nakagawa O. Ozumi K. Lessner S. M. Aoki H. (2008). Novel role of antioxidant-1 (Atox1) as a copper-dependent transcription factor involved in cell proliferation. J. Biol. Chem. 283 (14), 9157–9167. 10.1074/jbc.M709463200 18245776 PMC2431038

[B84] Jandial D. D. Farshchi-Heydari S. Larson C. A. Elliott G. I. Wrasidlo W. J. Howell S. B. (2009). Enhanced delivery of cisplatin to intraperitoneal ovarian carcinomas mediated by the effects of bortezomib on the human copper transporter 1. Clin. Cancer Res. 15 (2), 553–560. 10.1158/1078-0432.CCR-08-2081 19147760 PMC2707998

[B85] Ji Q. X. Zeng F. Y. Zhou J. Wu W. B. Wang X. J. Zhang Z. (2023). Ferroptotic stress facilitates smooth muscle cell dedifferentiation in arterial remodelling by disrupting mitochondrial homeostasis. Cell Death Differ. 30, 457–474. 10.1038/s41418-022-01099-5 36477078 PMC9950429

[B86] Jiang F. Du L. Chen Z. J. Wang X. Ge D. Liu N. (2023). LNP-miR-155 cy5 inhibitor regulates the copper transporter via the β-Catenin/TCF4/SLC31A1 signal for colorectal cancer therapy. Mol. Pharm. 20 (8), 4138–4152. 10.1021/acs.molpharmaceut.3c00276 37358225

[B87] Jiang M. Zhang Z. Li W. Man X. Sun H. Liang H. (2022b). Developing a copper(II) agent based on His-146 and His-242 residues of human serum Albumin nanoparticles: integration to overcome cisplatin resistance and inhibit the metastasis of Nonsmall cell lung cancer. J. Med. Chem. 65 (13), 9447–9458. 10.1021/acs.jmedchem.2c00698 35786921

[B88] Jiang Y. Liu Y. Wang M. Li Z. Su L. Xu X. (2022c). siRNA-based carrier-free system for synergistic chemo/chemodynamic/RNAi therapy of drug-resistant tumors. ACS Appl. Mater. Interfaces 14 (1), 361–372. 10.1021/acsami.1c20898 34931793

[B89] Jiang Y. C. Huo Z. Y. Qi X. L. Zuo T. M. Wu Z. H. (2022a). Copper-induced tumor cell death mechanisms and antitumor theragnostic applications of copper complexes. Nanomedicine 17 (5), 303–324. 10.2217/nnm-2021-0374 35060391

[B90] Jin J. Ma M. Shi S. Wang J. Xiao P. Yu H. F. (2022). Copper enhances genotoxic drug resistance via ATOX1 activated DNA damage repair. Cancer Lett. 536, 215651. 10.1016/j.canlet.2022.215651 35315340

[B91] Kahlson M. A. Dixon S. J. (2022). Copper-induced cell death. Science 375, 1231–1232. 10.1126/science.abo3959 35298241

[B92] Katsuyama M. Kimura E. Ibi M. Iwata K. Matsumoto M. Asaoka N. (2021). Clioquinol inhibits dopamine-β-hydroxylase secretion and noradrenaline synthesis by affecting the redox status of ATOX1 and copper transport in human neuroblastoma SH-SY5Y cells. Arch. Toxikol. 95, 135–148. 10.1007/s00204-020-02894-0 33034664

[B93] Kazakova O. B. Giniyatullina G. V. Mustafin A. G. Babkov D. A. Sokolova E. V. Spasov A. A. (2020). Evaluation of cytotoxicity and α-Glucosidase inhibitory activity of Amide and Polyamino-Derivatives of Lupane Triterpenoids. Molecules 25 (20), 4833. 10.3390/molecules25204833 33092246 PMC7587962

[B94] Kim B. M. Rhee J. S. Park G. S. Lee J. Lee Y. M. Lee J. S. (2011). Cu/Zn- and Mn-superoxide dismutase (SOD) from the copepod Tigriopus japonicus: molecular cloning and expression in response to environmental pollutants. Chemosphere 84 (10), 1467–1475. 10.1016/j.chemosphere.2011.04.043 21550634

[B95] Kim E. S. Tang X. Peterson D. R. Kilari D. Chow C. W. Fujimoto J. (2014). Copper transporter CTR1 expression and tissue platinum concentration in non-small cell lung cancer. Lung Cancer 85 (1), 88–93. 10.1016/j.lungcan.2014.04.005 24792335 PMC4090351

[B96] Kim K. K. Han A. Yano N. Ribeiro J. R. Lokich E. Singh R. K. (2015). Tetrathiomolybdate mediates cisplatin-induced p38 signaling and EGFR degradation and enhances response to cisplatin therapy in gynecologic cancers. Sci. Rep. 5, 15911. 10.1038/srep15911 26568478 PMC4644948

[B97] King S. A. Solst S. R. Graham C. H. Fiore L. Z. Rheem R. Tomanek-Chalkley A. (2024). Additive effects of Cu-ATSM and radiation on survival of diffuse intrinsic pontine glioma cells. Radiat. Res. 203 (1), 10–17. 10.1667/RADE-24-00076.1 PMC11815956 39492578

[B98] Koraishy F. M. Cohen R. A. Israel G. M. Dahl N. K. (2013). Cystic kidney disease in a patient with systemic toxicity from long-term D-penicillamine use. Am. J. Kidney Dis. 62, 806–809. 10.1053/j.ajkd.2013.04.017 23796907

[B99] Kothapalli C. R. Ramamurthi A. (2009). Biomimetic regeneration of elastin matrices using hyaluronan and copper ion cues. Tissue Eng. Part. A 15, 103–113. 10.1089/ten.tea.2007.0390 18847363 PMC2792045

[B100] Krupanidhi S. Sreekumar A. Sanjeevi C. B. (2008). Copper and biological health. Indian J. Med. Res. 128, 448–461. 19106440

[B101] Kuo M. T. Huang Y. F. Chou C. Y. Chen H. H. W. (2021). Targeting the copper transport system to improve treatment Efficacies of platinum-containing drugs in cancer chemotherapy. Pharmaceuticals 14 (6), 549. 10.3390/ph14060549 34201235 PMC8227247

[B102] Lei G. Tang L. Yu Y. Bian W. Yu L. Zhou J. (2023). The potential of targeting cuproptosis in the treatment of kidney renal clear cell carcinoma. Biomed. Pharmacother. 167, 115522. 10.1016/j.biopha.2023.115522 37757497

[B103] Levy A. R. Turgeman M. Gevorkyan-Aiapetov L. Ruthstein S. (2017). The structural flexibility of the human copper chaperone Atox1: insights from combined pulsed EPR studies and computations. Protein Sci. 26, 1609–1618. 10.1002/pro.3197 28543811 PMC5521546

[B104] Li H. Wang J. Wu C. Wang L. Chen Z. S. Cui W. (2020). The combination of disulfiram and copper for cancer treatment. Drug Discov. Today 25, 1099–1108. 10.1016/j.drudis.2020.04.003 32320854

[B105] Li L. Zhou H. Zhang C. (2024b). Cuproptosis in cancer: biological implications and therapeutic opportunities. Cell. Mol. Biol. Lett. 29 (1), 91. 10.1186/s11658-024-00608-3 38918694 PMC11201306

[B106] Li P. Sun Q. Bai S. Wang H. Zhao L. (2024a). Combination of the cuproptosis inducer disulfiram and anti-PD-L1 abolishes NSCLC resistance by ATP7B to regulate the HIF-1 signaling pathway. Int. J. Mol. Med. 53, 19. 10.3892/ijmm.2023.5343 38186308 PMC10781418

[B107] Li R. Huang Y. J. Liu H. R. Dilger J. P. Lin J. (2018a). Abstract 2162: Comparing volatile and intravenous anesthetics in a mouse model of breast cancer metastasis. Cancer Res. 78 (13), 2162. 10.1158/1538-7445.AM2018-2162

[B108] Li X. Tang C. Ye H. Fang C. (2024c). Injectable hydrogel-encapsulating Pickering emulsion for overcoming lenvatinib-resistant hepatocellular carcinoma via cuproptosis induction and stemness inhibition. Polymers 16 (17), 2418. 10.3390/polym16172418 39274051 PMC11397159

[B109] Li Y. Fang M. Xu Z. Li X. (2022). Tetrathiomolybdate as an old drug in a new use: as a chemotherapeutic sensitizer for non-small cell lung cancer. J. Inorg. Biochem. 233, 111865. 10.1016/j.jinorgbio.2022.111865 35623139

[B110] Li Y. Q. Yin J. Y. Liu Z. Q. Li X. P. (2018b). Copper efflux transporters ATP7A and ATP7B: novel biomarkers for platinum drug resistance and targets for therapy. IUBMB life 70 (3), 183–191. 10.1002/iub.1722 29394468

[B111] Li Z. Zhou H. Zhai X. Gao L. Yang M. An B. (2023). MELK promotes HCC carcinogenesis through modulating cuproptosis-related gene DLAT-mediated mitochondrial function. Cell death Dis. 14 (11), 733. 10.1038/s41419-023-06264-3 37949877 PMC10638394

[B112] Liao Y. Wang D. Gu C. Wang X. Zhu S. Zheng Z. (2024). A cuproptosis nanocapsule for cancer radiotherapy. Nat. Nanotechnol. 19 (12), 1892–1902. 10.1038/s41565-024-01784-1 39300223

[B113] Lin P. H. Chen M. Tsai L. W. Lo C. Yen T. C. Huang T. Y. (2020a). Using next-generation sequencing to redefine BRCAness in triple-negative breast cancer. Cancer Sci. 111 (4), 1375–1384. 10.1111/cas.14313 31958182 PMC7156820

[B114] Lin S. C. Tsai Y. C. Chen Y. L. Lin H. K. Huang Y. C. Lin Y. S. (2024). Un-methylation of NUDT21 represses docosahexaenoic acid biosynthesis contributing to enzalutamide resistance in prostate cancer. Drug resist. updat. 77, 101144. 10.1016/j.drup.2024.101144 39208673

[B115] Lin Z. Niu Y. Wan A. Chen D. Liang H. Chen X. (2020b). RNA m6 A methylation regulates sorafenib resistance in liver cancer through FOXO3-mediated autophagy. EMBO J. 39 (12), e103181. 10.15252/embj.2019103181 32368828 PMC7298296

[B116] Liu H. (2023). Expression and potential immune involvement of cuproptosis in kidney renal clear cell carcinoma. Cancer genet. 274-275, 21–25. 10.1016/j.cancergen.2023.03.002 36963335

[B117] Liu H. Bao M. Liu M. Deng F. Wen X. Wan P. (2024). The association between serum copper and bone Mineral density among Adolescents aged 12 to 19 in the United States. Nutrients 16, 453. 10.3390/nu16030453 38337737 PMC10857197

[B118] Liu H. Tang T. (2022a). Pan-cancer genetic analysis of cuproptosis and copper metabolism-related gene set. Front. Oncol. 12, 952290. 10.3389/fonc.2022.952290 36276096 PMC9582932

[B119] Liu H. Tang T. (2022b). Pan-cancer genetic analysis of cuproptosis and copper metabolism-related gene set. Am. J. Cancer Res. 12 (8), 4074–4081. 10.3389/fonc.2022.952290 36276096 PMC9582932

[B120] Liu H. Tang T. (2023). Pan-cancer genetic analysis of disulfidptosis-related gene set. Cancer genet. 278-279, 91–103. 10.1016/j.cancergen.2023.10.001 37879141

[B121] Liu H. Wang P. (2024). CRISPR screening and cell line IC_50_ data reveal novel key genes for trametinib resistance. Clin. Exp. Med. 25 (1), 21. 10.1007/s10238-024-01538-2 39708249 PMC11663197

[B122] Liu X. Luo B. Wu X. Tang Z. (2023). Cuproptosis and cuproptosis-related genes: emerging potential therapeutic targets in breast cancer. Biochim. Biophys. Acta Rev. Cancer 1878 (6), 189013. 10.1016/j.bbcan.2023.189013 37918452

[B123] Llovet J. M. Castet F. Heikenwalder M. Maini M. K. Mazzaferro V. Pinato D. J. (2022). Immunotherapies for hepatocellular carcinoma. Nat. Rev. Clin. Oncol. 19 (3), 151–172. 10.1038/s41571-021-00573-2 34764464

[B124] Lu J. Liu X. Li X. Li H. Shi L. Xia X. (2024c). Copper regulates the host innate immune response against bacterial infection via activation of ALPK1 kinase. Proc. Natl. Acad. Sci. U. S. A. 121, e2311630121. 10.1073/pnas.2311630121 38232278 PMC10823219

[B125] Lu S. Ke Y. Wu C. Zhong Y. Xie C. Zhou Y. (2018). Radiosensitization of clioquinol and zinc in human cancer cell lines. BMC Cancer 18, 448. 10.1186/s12885-018-4264-2 29678153 PMC5910585

[B126] Lu S. Tian H. Li B. Li L. Jiang H. Gao Y. (2024b). An ellagic acid coordinated copper-based nanoplatform for Efficiently overcoming cancer chemoresistance by cuproptosis and synergistic inhibition of cancer cell stemness. Small 20 (17), e2309215. 10.1002/smll.202309215 38044295

[B127] Lu X. Chen X. Lin C. Yi Y. Zhao S. Zhu B. (2024a). Elesclomol loaded copper oxide nanoplatform triggers cuproptosis to enhance antitumor immunotherapy. Adv. Sci. 11 (18), e2309984. 10.1002/advs.202309984 PMC11095170 38430531

[B128] Lu Y. Pan Q. Gao W. Pu Y. Luo K. He B. (2022). Leveraging disulfiram to treat cancer: mechanisms of action, delivery strategies, and treatment regimens. Biomaterials 281, 121335. 10.1016/j.biomaterials.2021.121335 34979419

[B129] Lukanović D. Herzog M. Kobal B. Černe K. (2020). The contribution of copper efflux transporters ATP7A and ATP7B to chemoresistance and personalized medicine in ovarian cancer. Biomed. Pharmacother. 129, 110401. 10.1016/j.biopha.2020.110401 32570116

[B130] Luo Y. Luo X. Ru Y. Zhou X. Liu D. Huang Q. (2024). Copper(II)-Based Nano-regulator correlates cuproptosis Burst and Sequential immunogenic cell death for synergistic cancer immunotherapy. Biomater. Res. 28, 0039. 10.34133/bmr.0039 38938647 PMC11208873

[B131] Madkour M. M. Ramadan W. S. Saleh E. El-Awady R. (2023). Epigenetic modulations in cancer: predictive biomarkers and potential targets for overcoming the resistance to topoisomerase I inhibitors. Ann. Med. 55 (1), 2203946. 10.1080/07853890.2023.2203946 37092854 PMC10128461

[B132] Mammoto T. Jiang A. Jiang E. Panigrahy D. Kieran M. W. Mammoto A. (2013). Role of collagen matrix in tumor angiogenesis and glioblastoma multiforme progression. Am. J. Pathol. 183, 1293–1305. 10.1016/j.ajpath.2013.06.026 23928381 PMC3791684

[B133] Marelja Z. Leimkühler S. Missirlis F. (2018). Iron sulfur and Molybdenum cofactor enzymes regulate the Drosophila life cycle by controlling cell metabolism. Front. Physiol. 9, 50. 10.3389/fphys.2018.00050 29491838 PMC5817353

[B134] Mehta A. K. Cheney E. M. Hartl C. A. Pantelidou C. Oliwa M. Castrillon J. A. (2021). Targeting immunosuppressive macrophages overcomes PARP inhibitor resistance in BRCA1-associated triple-negative breast cancer. Nat. Cancer 2 (1), 66–82. 10.1038/s43018-020-00148-7 33738458 PMC7963404

[B135] Mi X. Li Z. Yan J. Li Y. Zheng J. Zhuang Z. (2020). Activation of HIF-1 signaling ameliorates liver steatosis in zebrafish atp7b deficiency (Wilson's disease) models. Biochim. Biophys. Acta. Mol. Basis Dis. 1866 (10), 165842. 10.1016/j.bbadis.2020.165842 32446740

[B136] Mirman Z. Sasi N. K. King A. Chapman J. R. de Lange T. (2022). 53BP1-shieldin-dependent DSB processing in BRCA1-deficient cells requires CST-Polα-primase fill-in synthesis. Nat. cell Biol. 24 (1), 51–61. 10.1038/s41556-021-00812-9 35027730 PMC8849574

[B137] Modica-Napolitano J. S. Bharath L. P. Hanlon A. J. Hurley L. D. (2019). The anticancer agent elesclomol has direct effects on mitochondrial Bioenergetic function in Isolated mammalian mitochondria. Biomolecules 9 (8), 298. 10.3390/biom9080298 31344923 PMC6724019

[B138] Molinaro C. Wambang N. Bousquet T. Vercoutter-Edouart A. S. Pélinski L. Cailliau K. (2022). A novel copper(II) Indenoisoquinoline complex inhibits topoisomerase I, induces G2 phase arrest, and autophagy in three adenocarcinomas. Front. Oncol. 12, 837373. 10.3389/fonc.2022.837373 35280788 PMC8908320

[B139] Morisawa A. Okui T. Shimo T. Ibaragi S. Okusha Y. Ono M. (2018). Ammonium tetrathiomolybdate enhances the antitumor effects of cetuximab via the suppression of osteoclastogenesis in head and neck squamous carcinoma. Int. J. Oncol. 52, 989–999. 10.3892/ijo.2018.4242 29328370

[B140] Muñoz C. Rios E. Olivos J. Brunser O. Olivares M. (2007). Iron, copper and immunocompetence. Br. J. Nutr. 98 (Suppl. 1), S24–S28. 10.1017/S0007114507833046 17922954

[B141] Nagai M. Blackman R. K. Rao P. E. Wada Y. Koya K. (2010). Abstract 4545: anticancer activity of elesclomol correlates with low ldh levels and active mitochondrial respiration. Cancer Res. 70 (8 Suppl. ment), 4545. 10.1158/1538-7445.AM10-4545

[B142] Nath A. Cosgrove P. A. Chang J. T. Bild A. H. (2022). Predicting clinical response to everolimus in ER+ breast cancers using machine-learning. Front. Mol. Biosci. 9, 981962. 10.3389/fmolb.2022.981962 36304922 PMC9592823

[B143] Obata A. Yoshimi E. Waki A. Lewis J. S. Oyama N. Welch M. J. (2001). Retention mechanism of hypoxia selective nuclear imaging/radiotherapeutic agent cu-diacetyl-bis(N4-methylthiosemicarbazone) (Cu-ATSM) in tumor cells. Ann. Nucl. Med. 15, 499–504. 10.1007/BF02988502 11831397

[B144] Ogen-Shtern N. Chumin K. Cohen G. Borkow G. (2020). Increased pro-collagen 1, elastin, and TGF-β1 expression by copper ions in an *ex-vivo* human skin model. J. Cosmet. Dermatol. 19, 1522–1527. 10.1111/jocd.13186 31603269

[B145] Öhrvik H. Logeman B. Turk B. Reinheckel T. Thiele D. J. (2016). Cathepsin Protease controls copper and cisplatin accumulation via cleavage of the Ctr1 metal-binding Ectodomain. J. Biol. Chem. 291, 13905–13916. 10.1074/jbc.M116.731281 27143361 PMC4933151

[B146] Öhrvik H. Thiele D. J. (2015). The role of Ctr1 and Ctr2 in mammalian copper homeostasis and platinum-based chemotherapy. J. Trace. Elem. Med. Biol. 31, 178–182. 10.1016/j.jtemb.2014.03.006 24703712 PMC4175275

[B147] Panda S. Setia M. Kaur N. Shepal V. Arora V. Singh D. K. (2018). Noncoding RNA Ginir functions as an oncogene by associating with centrosomal proteins. PLoS Biol. 16 (10), e2004204. 10.1371/journal.pbio.2004204 30296263 PMC6193740

[B148] Parmar A. Pascali G. Voli F. Lerra L. Yee E. Ahmed-Cox A. (2018). *In vivo* [^64^ Cu]CuCl_2_ PET imaging reveals activity of Dextran-Catechin on tumor copper homeostasis. Theranostics 8 (20), 5645–5659. 10.7150/thno.29840 30555570 PMC6276294

[B149] Percival S. S. (1998). Copper and immunity. Am. J. Clin. Nutr. 67, 1064S–1068S. 10.1093/ajcn/67.5.1064S 9587153

[B150] Pérez-Henarejos S. A. Alcaraz L. A. Donaire A. (2015). Blue Copper Proteins: a rigid machine for efficient electron transfer, a flexible device for metal uptake. Arch. Biochem. Biophys. 584, 134–148. 10.1016/j.abb.2015.08.020 26334718

[B151] Petruzzelli R. Mariniello M. De Cegli R. Catalano F. Guida F. Di Schiavi E. (2022). TFEB regulates ATP7B expression to promote platinum chemoresistance in human ovarian cancer cells. Cells 11 (2), 219. 10.3390/cells11020219 35053335 PMC8774088

[B152] Poulose A. C. Veeranarayanan S. Mohamed M. S. Aburto R. R. Mitcham T. Bouchard R. R. (2016). Multifunctional Cu_2-x_Te Nanocubes mediated combination therapy for multi-drug resistant MDA MB 453. Sci. Rep. 6, 35961. 10.1038/srep35961 27775048 PMC5075932

[B153] Qian L. Murakami K. Toyozumi T. Matsumoto Y. Otsuka R. Sekino N. (2023). An anti-alcoholism drug, disulfiram and copper complex improves radio-resistance of tumor-initiating cells in esophageal squamous cell carcinoma. Esophagus 20 (1), 134–142. 10.1007/s10388-022-00948-z 36121574

[B154] Qin Y. Liu Y. Xiang X. Long X. Chen Z. Huang X. (2023). Cuproptosis correlates with immunosuppressive tumor microenvironment based on pan-cancer multiomics and single-cell sequencing analysis. Mol. Cancer 22, 59. 10.1186/s12943-023-01752-8 36959665 PMC10037895

[B155] Quan Y. Li W. Yan R. Cheng J. Xu H. Chen L. (2023). Tumor cuproptosis and immune infiltration improve survival of patients with hepatocellular carcinoma with a high expression of ferredoxin 1. Front. Oncol. 13, 1168769. 10.3389/fonc.2023.1168769 37361595 PMC10285401

[B156] Rebollo-Hernanz M. Aguilera Y. Martin-Cabrejas M. A. de Mejia E. (2022). Phytochemicals from the Cocoa Shell modulate mitochondrial function, lipid and glucose metabolism in Hepatocytes via activation of FGF21/ERK, AKT, and mTOR pathways. Antioxidants (Basel) 11 (1), 136. 10.3390/antiox11010136 35052640 PMC8772970

[B157] Ren X. Li Y. Zhou Y. Hu W. Yang C. Jing Q. (2021). Overcoming the compensatory elevation of NRF2 renders hepatocellular carcinoma cells more vulnerable to disulfiram/copper-induced ferroptosis. Redox Biol. 46, 102122. 10.1016/j.redox.2021.102122 34482117 PMC8416961

[B158] Ren X. Luo X. Wang F. Wan L. Wang X. Xiong J. (2024). Recent advances in copper homeostasis-involved tumor theranostics. Asian J. Pharm. Sci. 19 (5), 100948. 10.1016/j.ajps.2024.100948 39474127 PMC11513462

[B159] Rihel J. (2018). Copper on the brain. Nat. Chem. Biol. 14, 638–639. 10.1038/s41589-018-0089-1 29915234

[B160] Rivera P. L. Li W. T. Bhogal S. Mandell J. B. Belayneh R. Hankins M. L. (2023). Antioxidant 1 copper chaperone gene expression and copper levels in dog osteosarcoma patients. Vet. Comp. Oncol. 21, 559–564. 10.1111/vco.12903 37148200 PMC11231990

[B161] Roig-Carles D. Jackson H. Loveson K. F. Mackay A. Mather R. L. Waters E. (2021). The long non-Coding RNA H19 drives the proliferation of diffuse intrinsic pontine glioma with H3K27 mutation. Int. J. Mol. Sci. 22 (17), 9165. 10.3390/ijms22179165 34502082 PMC8431314

[B162] Ruiz L. M. Libedinsky A. Elorza A. A. (2021). Role of copper on mitochondrial function and metabolism. Front. Mol. Biosci. 8, 711227. 10.3389/fmolb.2021.711227 34504870 PMC8421569

[B163] Ryumon S. Okui T. Kunisada Y. Kishimoto K. Shimo T. Hasegawa K. (2019). Ammonium tetrathiomolybdate enhances the antitumor effect of cisplatin via the suppression of ATPase copper transporting beta in head and neck squamous cell carcinoma. Oncol. Rep. 42 (6), 2611–2621. 10.3892/or.2019.7367 31638244 PMC6826331

[B164] Safi R. Nelson E. R. Chitneni S. K. Franz K. J. George D. J. Zalutsky M. R. (2014). Copper signaling axis as a target for prostate cancer therapeutics. Cancer Res. 74 (20), 5819–5831. 10.1158/0008-5472.CAN-13-3527 25320179 PMC4203427

[B165] Salguero I. Belotserkovskaya R. Coates J. Sczaniecka-Clift M. Demir M. Jhujh S. (2019). MDC1 PST-repeat region promotes histone H2AX-independent chromatin association and DNA damage tolerance. Nat. Commun. 10 (1), 5191. 10.1038/s41467-019-12929-5 31729360 PMC6858307

[B166] Samimi G. Safaei R. Katano K. Holzer A. K. Rochdi M. Tomioka M. (2004). Increased expression of the copper efflux transporter ATP7A mediates resistance to cisplatin, carboplatin, and oxaliplatin in ovarian cancer cells. Clin. Cancer Res. 10 (14), 4661–4669. 10.1158/1078-0432.CCR-04-0137 15269138

[B167] Sauna Z. E. Peng X. H. Nandigama K. Tekle S. Ambudkar S. V. (2004). The molecular basis of the action of disulfiram as a modulator of the multidrug resistance-linked ATP binding cassette transporters MDR1 (ABCB1) and MRP1 (ABCC1). Mol. Pharmacol. 65 (3), 675–684. 10.1124/mol.65.3.675 14978246

[B168] Schimmer A. D. (2011). Clioquinol-a novel copper-dependent and independent proteasome inhibitor. Curr. Cancer Drug Targets 11, 325–331. 10.2174/156800911794519770 21247386

[B169] Schmidt K. Ralle M. Schaffer T. Jayakanthan S. Bari B. Muchenditsi A. (2018). ATP7A and ATP7B copper transporters have distinct functions in the regulation of neuronal dopamine-β-hydroxylase. J. Biol. Chem. 293 (52), 20085–20098. 10.1074/jbc.RA118.004889 30341172 PMC6311498

[B170] Schulz V. Basu S. Freibert S. A. Webert H. Boss L. Mühlenhoff U. (2023). Functional spectrum and specificity of mitochondrial ferredoxins FDX1 and FDX2. Nat. Chem. Biol. 19, 206–217. 10.1038/s41589-022-01159-4 36280795 PMC10873809

[B171] Shanbhag V. Jasmer-McDonald K. Zhu S. Martin A. L. Gudekar N. Khan A. (2019). ATP7A delivers copper to the lysyl oxidase family of enzymes and promotes tumorigenesis and metastasis. Proc. Natl. Acad. Sci. U. S. A. 116 (14), 6836–6841. 10.1073/pnas.1817473116 30890638 PMC6452744

[B172] Shao K. Shen H. Chen X. Shao Z. Liu Y. Wang Y. (2023). Copper transporter gene ATP7A: a predictive biomarker for immunotherapy and targeted therapy in hepatocellular carcinoma. Int. Immunopharmacol. 114, 109518. 10.1016/j.intimp.2022.109518 36502594

[B173] Sheftel A. D. Stehling O. Pierik A. J. Elsässer H. P. Mühlenhoff U. Webert H. (2010). Humans possess two mitochondrial ferredoxins, Fdx1 and Fdx2, with distinct roles in steroidogenesis, heme, and Fe/S cluster biosynthesis. Proc. Natl. Acad. Sci. U. S. A. 107 (26), 11775–11780. 10.1073/pnas.1004250107 20547883 PMC2900682

[B174] Shen D. Lin J. Xie Y. Zhuang Z. Xu G. Peng S. (2023). RNA demethylase ALKBH5 promotes colorectal cancer progression by posttranscriptional activation of RAB5A in an m6A-YTHDF2-dependent manner. Clin. Transl. Med. 13 (5), e1279. 10.1002/ctm2.1279 37203239 PMC10196219

[B175] Siegel R. L. Giaquinto A. N. Jemal A. (2024). Cancer statistics, 2024. CA Cancer J. Clin. 74 (1), 12–49. 10.3322/caac.21820 38230766

[B176] Singla A. Chen Q. Suzuki K. Song J. Fedoseienko A. Wijers M. (2021). Regulation of murine copper homeostasis by members of the COMMD protein family. Dis. Model Mech. 14 (1), dmm045963. 10.1242/dmm.045963 33262129 PMC7803461

[B177] Skowron M. A. Melnikova M. van Roermund J. G. H. Romano A. Albers P. Thomale J. (2018). Multifaceted mechanisms of cisplatin resistance in long-term treated urothelial carcinoma cell lines. Int. J. Mol. Sci. 19 (2), 590. 10.3390/ijms19020590 29462944 PMC5855812

[B178] Skrott Z. Mistrik M. Andersen K. K. Friis S. Majera D. Gursky J. (2017). Alcohol-abuse drug disulfiram targets cancer via p97 segregase adaptor NPL4. Nature 552 (7684), 194–199. 10.1038/nature25016 29211715 PMC5730499

[B179] Smirnova J. Kabin E. Järving I. Bragina O. Tõugu V. Plitz T. (2018). Copper(I)-binding properties of de-coppering drugs for the treatment of Wilson disease. α-Lipoic acid as a potential anti-copper agent. Sci. Rep. 8 (1), 1463. 10.1038/s41598-018-19873-2 29362485 PMC5780470

[B180] Song L. Nguyen V. Xie J. Jia S. Chang C. J. Uchio E. (2024). ATPase copper transporting beta (ATP7B) is a novel target for improving the therapeutic efficacy of docetaxel by disulfiram/copper in human prostate cancer. Mol. Cancer Ther. 23 (6), 854–863. 10.1158/1535-7163.MCT-23-0876 38417139 PMC11150099

[B181] Sonkin D. Thomas A. Teicher B. A. (2024). Cancer treatments: past, present, and future. Cancer Genet. 286-287, 18–24. 10.1016/j.cancergen.2024.06.002 38909530 PMC11338712

[B182] Sotgia F. Fiorillo M. Lisanti M. P. (2017). Mitochondrial markers predict recurrence, metastasis and tamoxifen-resistance in breast cancer patients: early detection of treatment failure with companion diagnostics. Oncotarget 8 (40), 68730–68745. 10.18632/oncotarget.19612 28978152 PMC5620292

[B183] Spiers J. G. Tan L. S. Anderson S. T. Hill A. F. Lavidis N. A. Chen H. C. (2021). Hepatic homeostasis of metal ions following acute repeated stress exposure in Rats. Antioxidants (Basel) 11 (1), 85. 10.3390/antiox11010085 35052588 PMC8773239

[B184] Steinbrueck A. Sedgwick A. C. Brewster J. T. Yan K. C. Shang Y. Knoll D. M. (2020). Transition metal chelators, pro-chelators, and ionophores as small molecule cancer chemotherapeutic agents. Chem. Soc. Rev. 49, 3726–3747. 10.1039/c9cs00373h 32525153

[B185] Sudhahar V. Shi Y. Kaplan J. H. Ushio-Fukai M. Fukai T. (2022). Whole-transcriptome sequencing Analyses of nuclear Antixoxidant-1 in endothelial cells: role in inflammation and Atherosclerosis. Cells 11 (18), 2919. 10.3390/cells11182919 36139494 PMC9496719

[B186] Sun H. Zou Y. Chen Z. He Y. Ye K. Liu H. (2025b). Nanodrug-engineered Exosomes Achieve a Jointly dual-pathway inhibition on cuproptosis. Adv. Sci. 12 (4), e2413408. 10.1002/advs.202413408 PMC11775538 39639737

[B187] Sun Z. Xu H. Lu G. Yang C. Gao X. Zhang J. (2025a). AKT1 phosphorylates FDX1 to promote cuproptosis resistance in triple-negative breast cancer. Adv. Sci., e2408106. 10.1002/advs.202408106 PMC12061301 39976173

[B188] Swaminathan A. B. Gohil V. M. (2022). The role of COA6 in the mitochondrial copper delivery pathway to cytochrome c oxidase. Biomolecules 12 (1), 125. 10.3390/biom12010125 35053273 PMC8773535

[B189] Swetha K. L. Paul M. Maravajjala K. S. Kumbham S. Biswas S. Roy A. (2023). Overcoming drug resistance with a docetaxel and disulfiram loaded pH-sensitive nanoparticle. J. Control. Release 356, 93–114. 10.1016/j.jconrel.2023.02.023 36841286

[B190] Tadini-Buoninsegni F. Bartolommei G. Moncelli M. R. Inesi G. Galliani A. Sinisi M. (2014). Translocation of platinum anticancer drugs by human copper ATPases ATP7A and ATP7B. Angew. Chem. Int. Ed. Engl. 53 (5), 1297–1301. 10.1002/anie.201307718 24375922 PMC3937162

[B191] Takahashi R. Kamizaki K. Yamanaka K. Terai Y. Minami Y. (2023). Expression of Ferredoxin1 in cisplatin-resistant ovarian cancer cells confers their resistance against ferroptosis induced by cisplatin. Oncol. Rep. 49 (6), 124. 10.3892/or.2023.8561 37144519 PMC10196713

[B192] Tang D. Kroemer G. Kang R. (2024). Targeting cuproplasia and cuproptosis in cancer. Nat. Rev. Clin. Oncol. 21 (5), 370–388. 10.1038/s41571-024-00876-0 38486054

[B193] Tang S. Bai L. Hou W. Hu Z. Chen X. Zhao J. (2022). Comparison of the effectiveness and safety of d-penicillamine and zinc Salt treatment for Symptomatic Wilson disease: a systematic review and Meta-analysis. Front. Pharmacol. 13, 847436. 10.3389/fphar.2022.847436 35370752 PMC8975209

[B194] Tarin M. Babaie M. Eshghi H. Matin M. M. Saljooghi A. S. (2023). Elesclomol, a copper-transporting therapeutic agent targeting mitochondria: from discovery to its novel applications. J. Transl. Med. 21, 745. 10.1186/s12967-023-04533-5 37864163 PMC10589935

[B195] Teschke R. Eickhoff A. (2024). Wilson disease: copper-mediated cuproptosis, iron-related ferroptosis, and clinical highlights, with comprehensive and critical analysis Update. Int. J. Mol. Sci. 25, 4753. 10.3390/ijms25094753 38731973 PMC11084815

[B196] Tian Z. Jiang S. Zhou J. Zhang W. (2023). Copper homeostasis and cuproptosis in mitochondria. Life Sci. 334, 122223. 10.1016/j.lfs.2023.122223 38084674

[B197] Tong X. Tang R. Xiao M. Xu J. Wang W. Zhang B. (2022). Targeting cell death pathways for cancer therapy: recent developments in necroptosis, pyroptosis, ferroptosis, and cuproptosis research. Haematologica 15, 174. 10.1186/s13045-022-01392-3 PMC9733270 36482419

[B198] Tsai C. Y. Finley J. C. Ali S. S. Patel H. H. Howell S. B. (2012). Copper influx transporter 1 is required for FGF, PDGF and EGF-induced MAPK signaling. Biochem. Pharmacol. 84, 1007–1013. 10.1016/j.bcp.2012.07.014 22842628 PMC3464187

[B199] Tsimberidou A. M. Said R. Culotta K. Wistuba I. Jelinek J. Fu S. (2015). Phase I study of azacitidine and oxaliplatin in patients with advanced cancers that have relapsed or are refractory to any platinum therapy. Clin. Epigenetics. 7, 29. 10.1186/s13148-015-0065-5 25806091 PMC4371799

[B200] Tsvetkov P. Coy S. Petrova B. Dreishpoon M. Verma A. Abdusamad M. (2022). Copper induces cell death by targeting lipoylated TCA cycle proteins. Science 375, 1254–1261. 10.1126/science.abf0529 35298263 PMC9273333

[B201] Tsvetkov P. Detappe A. Cai K. Keys H. R. Brune Z. Ying W. (2019). Mitochondrial metabolism promotes adaptation to proteotoxic stress. Nat. Chem. Biol. 15 (7), 681–689. 10.1038/s41589-019-0291-9 31133756 PMC8183600

[B202] Vanišová M. Burská D. Křížová J. Daňhelovská T. Dosoudilová Ž. Zeman J. (2019). Stable COX17 downregulation leads to alterations in mitochondrial Ultrastructure, decreased copper content and impaired cytochrome c oxidase Biogenesis in HEK293 cells. Folia Biol. (Praha) 65, 181–187. 10.14712/fb2019065040181 31903891

[B203] Wan H. Yang X. Sang G. Ruan Z. Ling Z. Zhang M. (2023). CDKN2A was a cuproptosis-related gene in regulating chemotherapy resistance by the MAGE-A family in breast cancer: based on artificial intelligence (AI)-constructed pan-cancer risk model. Aging 15 (20), 11244–11267. 10.18632/aging.205125 37857018 PMC10637804

[B204] Wang D. Yang L. Yu W. Wu Q. Lian J. Li F. (2019). Colorectal cancer cell-derived CCL20 recruits regulatory T cells to promote chemoresistance via FOXO1/CEBPB/NF-κB signaling. J. Immunother. Cancer 7 (1), 215. 10.1186/s40425-019-0701-2 31395078 PMC6688336

[B205] Wang H. Hu Q. Chen Y. Huang X. Feng Y. Shi Y. (2024f). Ferritinophagy mediates adaptive resistance to EGFR tyrosine kinase inhibitors in non-small cell lung cancer. Nat. Commun. 15 (1), 4195. 10.1038/s41467-024-48433-8 38760351 PMC11101634

[B206] Wang J. Li J. Liu J. Chan K. Y. Lee H. S. Lin K. N. (2024e). Interplay of ferroptosis and cuproptosis in cancer: Dissecting metal-driven mechanisms for therapeutic potentials. Cancers 16 (3), 512. 10.3390/cancers16030512 38339263 PMC10854932

[B207] Wang K. Michelakos T. Wang B. Shang Z. DeLeo A. B. Duan Z. (2021). Targeting cancer stem cells by disulfiram and copper sensitizes radioresistant chondrosarcoma to radiation. Cancer Lett. 505, 37–48. 10.1016/j.canlet.2021.02.002 33582212 PMC8969896

[B208] Wang L. Wang X. Zhu X. Zhong L. Jiang Q. Wang Y. (2024b). Drug resistance in ovarian cancer: from mechanism to clinical trial. Mol. Cancer 23 (1), 66. 10.1186/s12943-024-01967-3 38539161 PMC10976737

[B209] Wang M. Zheng L. Ma S. Lin R. Li J. Yang S. (2023a). Cuproptosis: emerging biomarkers and potential therapeutics in cancers. Front. Oncol. 13, 1288504. 10.3389/fonc.2023.1288504 38023234 PMC10662309

[B210] Wang N. N. Wang L. H. Li Y. Fu S. Y. Xue X. Jia L. N. (2018). Targeting ALDH2 with disulfiram/copper reverses the resistance of cancer cells to microtubule inhibitors. Exp. Cell Res. 362 (1), 72–82. 10.1016/j.yexcr.2017.11.004 29155365

[B211] Wang Q. Huang C. H. Wibowo F. S. Amin R. Shen J. Li F. (2024d). Elesclomol-copper nanoparticles overcome multidrug resistance in cancer cells. ACS Appl. Mater. Interfaces 16 (11), 13509–13524. 10.1021/acsami.3c17792 38466024

[B212] Wang S. Liu X. Wei D. Zhou H. Zhu J. Yu Q. (2024g). Polyvalent Aptamer nanodrug Conjugates enable efficient tumor cuproptosis therapy through copper overload and glutathione depletion. J. Am. Chem. Soc. 146 (44), 30033–30045. 10.1021/jacs.4c06338 39463177

[B213] Wang T. Liu Y. Li Q. Luo Y. Liu D. Li B. (2022). Cuproptosis-related gene FDX1 expression correlates with the prognosis and tumor immune microenvironment in clear cell renal cell carcinoma. Front. Immunol. 13, 999823. 10.3389/fimmu.2022.999823 36225932 PMC9549781

[B214] Wang W. Lu K. Jiang X. Wei Q. Zhu L. Wang X. (2023d). Ferroptosis inducers enhanced cuproptosis induced by copper ionophores in primary liver cancer. J. Exp. Clin. Canc. Res. 42, 142. 10.1186/s13046-023-02720-2 PMC10242978 37277863

[B215] Wang X. Chen D. Shi Y. Luo J. Zhang Y. Yuan X. (2023b). Copper and cuproptosis-related genes in hepatocellular carcinoma: therapeutic biomarkers targeting tumor immune microenvironment and immune checkpoints. Front. Immunol. 14, 1123231. 10.3389/fimmu.2023.1123231 37153542 PMC10157396

[B216] Wang X. Jia J. H. Zhang M. Meng Q. S. Yan B. W. Ma Z. Y. (2023c). Adrenomedullin/FOXO3 enhances sunitinib resistance in clear cell renal cell carcinoma by inhibiting FDX1 expression and cuproptosis. FASEB J. 37 (10), e23143. 10.1096/fj.202300474R 37698353

[B217] Wang X. Zhu J. Li L. Zhao Q. Huang Y. Wen C. (2024a). Utility of patient-derived xenografts to evaluate drug sensitivity and select optimal treatments for individual non-small-cell lung cancer patients. Mol. Med. 30 (1), 209. 10.1186/s10020-024-00934-4 39528952 PMC11556205

[B218] Wang Y. Chen Y. Zhang J. Yang Y. Fleishman J. S. Wang Y. (2024c). Cuproptosis: a novel therapeutic target for overcoming cancer drug resistance. Drug resist. updat. 72, 101018. 10.1016/j.drup.2023.101018 37979442

[B219] Wee N. K. Weinstein D. C. Fraser S. T. Assinder S. J. (2013). The mammalian copper transporters CTR1 and CTR2 and their roles in development and disease. Int. J. Biochem. Cell Biol. 45, 960–963. 10.1016/j.biocel.2013.01.018 23391749

[B220] Wei X. Xiong X. Wang P. Zhang S. Peng D. (2024). SIRT1-mediated deacetylation of FOXO3 enhances mitophagy and drives hormone resistance in endometrial cancer. Mol. Med. 30 (1), 147. 10.1186/s10020-024-00915-7 39266959 PMC11391609

[B221] Weishaupt A. K. Lamann K. Tallarek E. Pezacki A. T. Matier C. D. Schwerdtle T. (2024). Dysfunction in atox-1 and ceruloplasmin alters labile Cu levels and consequently Cu homeostasis in *C. elegans* . Front. Mol. Biosci. 11, 1354627. 10.3389/fmolb.2024.1354627 38389896 PMC10882093

[B222] Wen H. Qu C. Wang Z. Gao H. Liu W. Wang H. (2023). Cuproptosis enhances docetaxel chemosensitivity by inhibiting autophagy via the DLAT/mTOR pathway in prostate cancer. FASEB J. 37 (9), e23145. 10.1096/fj.202300980R 37584654

[B223] Wu S. Weng J. Pan Y. Wen Z. Zeng J. Lou Y. (2024). Disulfiram/Cu targeting FOXO6 modulates sensitivity of hepatocellular carcinoma to lenvatinib via disrupt choline metabolic. Cell. Signal. 127, 111563. 10.1016/j.cellsig.2024.111563 39694126

[B224] Wu S. J. Yang Y. Li F. P. Huang L. F. Han Z. H. Wang G. F. (2018). Chelerythrine induced cell death through ROS-dependent ER stress in human prostate cancer cells. Onco Targets Ther. 11, 2593–2601. 10.2147/OTT.S157707 29780252 PMC5951218

[B225] Wu W. Dong J. Lv Y. Chang D. (2022). Cuproptosis-Related genes in the prognosis of colorectal cancer and their correlation with the tumor microenvironment. Front. Genet. 13, 984158. 10.3389/fgene.2022.984158 36246586 PMC9554006

[B226] Xia C. Chen Y. Zhu Y. Chen D. Sun H. Shen T. (2024a). Identification of DLAT as a potential therapeutic target via a novel cuproptosis-related gene signature for the prediction of liver cancer prognosis. J. Gastrointest. Oncol. 15 (5), 2230–2251. 10.21037/jgo-24-609 39554575 PMC11565118

[B227] Xia J. Liu G. Wang C. Liu Z. Liu F. Li H. (2024b). One stone, three birds: Construction of Cu/ZIF-8@DSF@GOx/HA nanoplatform for synergistic starvation therapy enhanced chemo-/chemodynamic therapy. Nanomedicine 63, 102799. 10.1016/j.nano.2024.102799 39613128

[B228] Xiao Q. Ge G. (2012). Lysyl oxidase, extracellular matrix remodeling and cancer metastasis. Cancer Microenviron. 5 (3), 261–273. 10.1007/s12307-012-0105-z 22528876 PMC3460045

[B229] Xiao S. Gu H. Deng L. Yang X. Qiao D. Zhang X. (2023). Relationship between NUDT21 mediated alternative polyadenylation process and tumor. Front. Oncol. 13, 1052012. 10.3389/fonc.2023.1052012 36816917 PMC9933127

[B230] Xie F. Wei W. (2022). [^64^Cu]Cu-ATSM: an emerging theranostic agent for cancer and neuroinflammation. Eur. J. Nucl. Med. Mol. Imaging 49, 3964–3972. 10.1007/s00259-022-05887-6 35918492

[B231] Xie J. Liu J. Zhao M. Li X. Wang Y. Zhao Y. (2023b). Disulfiram/Cu kills and sensitizes BRAF-Mutant thyroid cancer cells to BRAF kinase inhibitor by ROS-Dependently Relieving feedback activation of MAPK/ERK and PI3K/AKT pathways. Int. J. Mol. Sci. 24, 3418. 10.3390/ijms24043418 36834830 PMC9968072

[B232] Xie J. Yang Y. Gao Y. He J. (2023a). Cuproptosis: mechanisms and links with cancers. Mol. Cancer 22, 46. 10.1186/s12943-023-01732-y 36882769 PMC9990368

[B233] Xu B. Shi P. Fombon I. S. Zhang Y. Huang F. Wang W. (2011). Disulfiram/copper complex activated JNK/c-jun pathway and sensitized cytotoxicity of doxorubicin in doxorubicin resistant leukemia HL60 cells. Blood Cells Mol. Dis. 47 (4), 264–269. 10.1016/j.bcmd.2011.08.004 21911303

[B234] Xu R. Zhang K. Liang J. Gao F. Li J. Guan F. (2021). Hyaluronic acid/polyethyleneimine nanoparticles loaded with copper ion and disulfiram for esophageal cancer. Carbohydr. Polym. 261, 117846. 10.1016/j.carbpol.2021.117846 33766342

[B235] Xue Q. Kang R. Klionsky D. J. Tang D. Liu J. Chen X. (2023). Copper metabolism in cell death and autophagy. Autophagy 19 (8), 2175–2195. 10.1080/15548627.2023.2200554 37055935 PMC10351475

[B236] Xue Y. Li P. D. Tang X. M. Yan Z. H. Xia S. S. Tian H. P. (2020). Cytochrome C oxidase assembly factor 1 Homolog Predicts poor prognosis and promotes cell proliferation in colorectal cancer by regulating PI3K/AKT signaling. Onco. Targets Ther. 13, 11505–11516. 10.2147/OTT.S279024 33204105 PMC7667209

[B237] Yang D. Xiao P. Qiu B. Yu H. F. Teng C. B. (2023). Copper chaperone antioxidant 1: multiple roles and a potential therapeutic target. J. Mol. Med. 101, 527–542. 10.1007/s00109-023-02311-w 37017692

[B238] Yang J. Chen N. Zhao P. Yang X. Li Y. Fu Z. (2024c). Diminished expression of gls in cd4 + t cells serves as a prognostic indicator associated with cuproptosis in septic patients. Shock 62 (1), 51–62. 10.1097/SHK.0000000000002370 38662604

[B239] Yang L. Yao C. Su Z. Fang Y. Pandey N. K. Amador E. (2024b). Combination of disulfiram and Copper-Cysteamine nanoparticles induces mitochondria damage and promotes apoptosis in endometrial cancer. Bioact. Mater. 36, 96–111. 10.1016/j.bioactmat.2024.02.009 38440322 PMC10911931

[B240] Yang L. Zhang Y. Wang Y. Jiang P. Liu F. Feng N. (2022b). Ferredoxin 1 is a cuproptosis-key gene responsible for tumor immunity and drug sensitivity: a pan-cancer analysis. Front. Pharmacol. 13, 938134. 10.3389/fphar.2022.938134 36210836 PMC9532935

[B241] Yang S. Li X. Yan J. Jiang F. Fan X. Jin J. (2024a). Disulfiram downregulates ferredoxin 1 to maintain copper homeostasis and inhibit inflammation in cerebral ischemia/reperfusion injury. Sci. Rep. 14, 15175. 10.1038/s41598-024-64981-x 38956251 PMC11219760

[B242] Yang Y. Li M. Sun X. Zhou C. Wang Y. Wang L. (2017). The selective cytotoxicity of DSF-Cu attributes to the biomechanical properties and cytoskeleton rearrangements in the normal and cancerous nasopharyngeal epithelial cells. Int. J. Biochem. Cell Biol. 84, 96–108. 10.1016/j.biocel.2017.01.007 28111334

[B243] Yang Y. Liang S. Geng H. Xiong M. Li M. Su Q. (2022a). Proteomics revealed the crosstalk between copper stress and cuproptosis, and explored the feasibility of curcumin as anticancer copper ionophore. Free. Radic. Biol. Med. 193, 638–647. 10.1016/j.freeradbiomed.2022.11.023 36395954

[B244] Yang Z. Guo F. Albers A. E. Sehouli J. Kaufmann A. M. (2019). Disulfiram modulates ROS accumulation and overcomes synergistically cisplatin resistance in breast cancer cell lines. Biomed. Pharmacothe. 113, 108727. 10.1016/j.biopha.2019.108727 30870721

[B245] Yap C. Short J. L. Nicolazzo J. A. (2021). A combination of clioquinol, zinc and copper increases the abundance and function of breast cancer resistance protein in human brain microvascular endothelial cells. J. Pharm. Sci. 110, 338–346. 10.1016/j.xphs.2020.04.010 32339529

[B246] Yin J. M. Sun L. B. Zheng J. S. Wang X. X. Chen D. X. Li N. (2016). Copper chelation by trientine dihydrochloride inhibits liver RFA-induced inflammatory responses *in vivo* . Inflamm. Res. 65, 1009–1020. 10.1007/s00011-016-0986-2 27613237

[B247] Yip N. C. Fombon I. S. Liu P. Brown S. Kannappan V. Armesilla A. L. (2011). Disulfiram modulated ROS-MAPK and NFκB pathways and targeted breast cancer cells with cancer stem cell-like properties. Br. J. Cancer 104, 1564–1574. 10.1038/bjc.2011.126 21487404 PMC3101904

[B248] Yoshii J. Yoshiji H. Kuriyama S. Ikenaka Y. Noguchi R. Okuda H. (2001). The copper-chelating agent, trientine, suppresses tumor development and angiogenesis in the murine hepatocellular carcinoma cells. Int. J. Cancer 94, 768–773. 10.1002/ijc.1537 11745476

[B249] Yu C. H. Dolgova N. V. Dmitriev O. Y. (2017). Dynamics of the metal binding domains and regulation of the human copper transporters ATP7B and ATP7A. IUBMB Life 69 (4), 226–235. 10.1002/iub.1611 28271598

[B250] Yu H. Lou J. R. Ding W. Q. (2010). Clioquinol independently targets NF-kappaB and lysosome pathways in human cancer cells. Anticancer Res. 30, 2087–2092. 20651355

[B251] Yu Z. Cao W. Ren Y. Zhang Q. Liu J. (2020). ATPase copper transporter A, negatively regulated by miR-148a-3p, contributes to cisplatin resistance in breast cancer cells. Clin. Transl. Med. 10 (1), 57–73. 10.1002/ctm2.19 32508020 PMC7240853

[B252] Zhang P. Li B. Chen Q. Wang H. Feng Q. (2022a). Glucose restriction induces ROS-AMPK-mediated CTR1 expression and increases cisplatin efficiency in NSCLC. Cancer Lett. 543, 215793. 10.1016/j.canlet.2022.215793 35716782

[B253] Zhang P. Zhou C. Ren X. Jing Q. Gao Y. Yang C. (2024). Inhibiting the compensatory elevation of xCT collaborates with disulfiram/copper-induced GSH consumption for cascade ferroptosis and cuproptosis. Redox Biol. 69, 103007. 10.1016/j.redox.2023.103007 38150993 PMC10788306

[B254] Zhang Q. Ma L. Zhou H. Zhou Y. Liu S. Li Q. (2022c). A prognostic signature of cuproptosis and TCA-related genes for hepatocellular carcinoma. Front. Oncol. 12, 1040736. 10.3389/fonc.2022.1040736 36324575 PMC9619237

[B255] Zhang X. Jiang Q. Su Y. Bu L. Sun Z. Wu X. (2023a). AMPK phosphorylates and stabilises copper transporter 1 to synergise metformin and copper chelator for breast cancer therapy. Br. J. Cancer 128 (8), 1452–1465. 10.1038/s41416-022-02127-4 36807336 PMC10070418

[B256] Zhang Y. Xie W. Li J. Liang Z. Zhou X. Tan Z. (2025). Precision targeted melanoma therapy via cuproptosis/chemodynamic and chemotherapy: an engineering MCHS-CuMOF nanodelivery system. Biomater. Adv. 171, 214228. 10.1016/j.bioadv.2025.214228 39983499

[B257] Zhang Y. Zhou Q. Lu L. Su Y. Shi W. Zhang H. (2023b). Copper induces Cognitive Impairment in mice via modulation of cuproptosis and CREB signaling. Nutrients 15, 972. 10.3390/nu15040972 36839332 PMC9958748

[B258] Zhang Z. Zeng X. Wu Y. Liu Y. Zhang X. Song Z. (2022b). Cuproptosis-related risk Score Predicts prognosis and Characterizes the tumor microenvironment in hepatocellular carcinoma. Front. Immunol. 13, 925618. 10.3389/fimmu.2022.925618 35898502 PMC9311491

[B259] Zhao X. Chen J. Yin S. Shi J. Zheng M. He C. (2022). The expression of cuproptosis-related genes in hepatocellular carcinoma and their relationships with prognosis. Front. Oncol. 12, 992468. 10.3389/fonc.2022.992468 36313717 PMC9614267

[B260] Zhao Z. Miao Z. Hou Y. Zhong Y. Zhang X. Fang X. (2024). A novel signature constructed by cuproptosis-related RNA methylation regulators suggesting downregulation of YTHDC2 may induce cuproptosis resistance in colorectal cancer. Int. Immunopharmacol. 139, 112691. 10.1016/j.intimp.2024.112691 39029230

[B261] Zheng P. Zhou C. Lu L. Liu B. Ding Y. (2022). Elesclomol: a copper ionophore targeting mitochondrial metabolism for cancer therapy. J. Exp. Clin. Cancer Res. 41 (1), 271. 10.1186/s13046-022-02485-0 36089608 PMC9465867

[B262] Zhou Y. Yang J. Huang L. Liu C. Yu M. Chen R. (2024). Nudt21-mediated alternative polyadenylation of MZT1 3'UTR contributes to pancreatic cancer progression. iScience 27 (2), 108822. 10.1016/j.isci.2024.108822 38303721 PMC10831950

[B263] Zhu S. Y. Zhou W. Q. Niu Y. Y. Zheng C. Liu X. Zhang Y. Y. (2023). COX17 restricts renal fibrosis development by maintaining mitochondrial copper homeostasis and restoring complex IV activity. Acta Pharmacol. Sin. 44, 2091–2102. 10.1038/s41401-023-01098-3 37217601 PMC10545728

[B264] Zulkifli M. Spelbring A. N. Zhang Y. Soma S. Chen S. Li L. (2023). FDX1-dependent and independent mechanisms of elesclomol-mediated intracellular copper delivery. Proc. Natl. Acad. Sci. U. S. A. 120, e2216722120. 10.1073/pnas.2216722120 36848556 PMC10013847

